# Transcranial Electrical Stimulation in Treatment of Depression: A Systematic Review and Meta-Analysis

**DOI:** 10.1001/jamanetworkopen.2025.16459

**Published:** 2025-06-18

**Authors:** Caili Ren, Sandeep R. Pagali, Zhen Wang, Simon Kung, Renu Bhargavi Boyapati, Karimul Islam, John W. Li, K. Maureen Shelton, Anne Waniger, Ann M. Rydberg, Leslie C. Hassett, Paul E. Croarkin, Brian N. Lundstrom, Alvaro Pascual-Leone, Maria I. Lapid

**Affiliations:** 1 Department of Psychiatry and Psychology, Mayo Clinic, Rochester, Minnesota; 2 Department of Medicine, Mayo Clinic, Rochester, Minnesota; 3 Department of Health Sciences Research, Mayo Clinic, Rochester, Minnesota; 4 Mayo Clinic Office of Postdoctoral Affairs and Research Training, Mayo Clinic, Rochester, Minnesota; 5 Department of Neurology, Mayo Clinic, Rochester, Minnesota; 6 Mayo Clinic Libraries, Mayo Clinic, Rochester, Minnesota; 7 Department of Rehabilitation Medicine, Wuxi Central Rehabilitation Hospital, The Affiliated Mental Health Center of Jiangnan University, Wuxi, Jiangsu, China; 8 Baton Rouge General Hospital, Baton Rouge, Louisiana; 9 Hinda and Arthur Marcus Institute for Aging Research, Deanna and Sidney Wolk Center for Memory Health, Hebrew SeniorLife,; 10 Department of Neurology, Harvard Medical School, Boston, Massachusetts

## Abstract

**Importance:**

The role and safety of transcranial electrical stimulation (tES) for treating depressive disorders remain under evaluation.

**Objective:**

To evaluate tES treatment in patients with major depressive disorder (MDD) and comorbid depressive conditions.

**Data Sources:**

A search of MEDLINE, Embase, Cochrane, APA PsycINFO, and Scopus databases was conducted from inception to September 17, 2024.

**Study Selection:**

Randomized clinical trials (RCTs) of adults with MDD, depression with psychiatric comorbidities (DPC), or depression with medical comorbidities (DMC), treated with transcranial direct current stimulation (tDCS), transcranial alternating current stimulation (tACS), or transcranial random noise stimulation (tRNS), compared with sham or other treatments were included.

**Data Extraction and Synthesis:**

Independent reviewers extracted data in accordance with Preferred Reporting Items for Systematic Reviews and Meta-Analyses 2020 guidelines, with random-effects meta-analysis used for pooling.

**Main Outcomes and Measures:**

Primary outcomes were depression severity, response and remission rates, and adverse events. Standardized mean differences (SMDs) were reported for continuous outcomes, and odds ratios (ORs) were reported for categorical outcomes. Quality of evidence (QOE) was assessed using the Grading of Recommendations Assessment, Development, and Evaluation criteria.

**Results:**

The meta-analysis included 5522 participants from 114 study groups from 88 RCTs (3198 female [58.9%]; mean [range] age, 43.1 [19.4-76.9] years). Most studies (104 study groups from 79 RCTs [91.2%]) evaluated tDCS, while 7 study groups from 6 RCTs (6.1%) evaluated tACS, and 3 study groups from 3 RCTs (2.7%) evaluated tRNS. tES was associated with reduced depressive symptoms (SMD = −0.59; 95% CI, −0.83 to −0.35; low QOE) and improvement in DMC (SMD = −1.05; 95% CI, −1.67 to −0.43; low QOE) and DPC (SMD = −0.78; 95% CI, −1.27 to −0.29; low QOE) compared with MDD (SMD = −0.22; 95% CI, −0.44 to 0.01; low QOE). tDCS was associated with reduced depression in DMC (SMD = −1.05; 95% CI, −1.70 to −0.40; very low QOE) and DPC (SMD = −0.88; 95% CI, −1.40 to −0.36; low QOE) but not MDD. tACS was associated with improved MDD symptoms (SMD = −0.58; 95% CI, −0.96 to −0.20; high QOE) and response rates (OR, 2.07; 95% CI, 1.34 to 3.19; high QOE). Combined tDCS and medication was associated with reduced symptoms (SMD = −0.51; 95% CI, −0.90 to −0.13; moderate QOE) and increased response (OR, 2.25; 95% CI, 1.08 to 4.65; high QOE) in MDD. tDCS combined with psychotherapy was not associated with improvement. Subgroup analysis showed that anodal left dorsolateral prefrontal cortex DCS was associated with improved outcomes. Mild to moderate adverse events were more frequent in tES groups.

**Conclusions and Relevance:**

In this systematic review and meta-analysis, tDCS was associated with improvement in depression among patients with DMC and DPC (with smaller benefits in MDD), tACS was associated with improved MDD outcomes (while tRNS had insufficient evidence) in smaller samples, and combined tDCS and medication was associated with improvement in depression. These findings suggest that tES is well-tolerated overall, with only mild to moderate adverse events, and that future research should optimize stimulation parameters and individualize tES interventions.

## Key Points

**Question:**

What is the role of transcranial electrical stimulation (tES) in depression treatment in individuals with major depressive disorder (MDD), depression with psychiatric comorbidities (DPC), and depression with medical comorbidities (DMC)?

**Findings:**

This systematic review and meta-analysis of 88 randomized clinical trials (5522 participants) found transcranial direct current stimulation and transcranial alternating current stimulation was associated with positive outcomes among patients with MDD and DPC or DMC, while transcranial random noise stimulation had insufficient evidence. Transcranial direct current stimulation combined with medication showed larger effect sizes in DMC and DPC, with smaller benefits in MDD, while transcranial alternating current stimulation was associated with improved depressive symptoms and response rates in MDD.

**Meaning:**

These findings suggest that tES is well-tolerated overall, with only mild to moderate adverse events; future studies should explore how to individualize tES interventions in patients with depression.

## Teaser

This systematic review and meta-analysis evaluates outcomes of various transcranial electrical stimulation treatments in patients with major depressive disorder and comorbid depressive conditions.

## Introduction

Depression, including major depressive disorder (MDD) and depression with psychiatric comorbidities (DPC) or depression with medical comorbidities (DMC), is a prevalent mental health condition that imposes a substantial global health burden. In the US, an estimated 8.8% of adults experience a major depressive episode annually, and many individuals face depression alongside other medical or psychiatric conditions, complicating treatment and exacerbating symptoms.^1^

Transcranial electrical stimulation (tES), encompassing transcranial direct current stimulation (tDCS), transcranial alternating current stimulation (tACS), and transcranial random noise stimulation (tRNS), has emerged as a promising noninvasive approach for treating depressive symptoms across depressive disorders.^2,3^ While previous meta-analyses suggest that tES modalities, particularly tDCS and tACS, correlate with improvement in treating MDD,^4,5,6,7^ these findings are preliminary, and no US Food and Drug Administration (FDA)–approved tES device or protocol exists. Several tES stimulation parameters (electrode size, location, current intensity, duration of treatment, and number of sessions) have been studied that limit 1 standardized protocol. In contrast, established brain stimulation methods such as electroconvulsive therapy and repetitive transcranial magnetic stimulation have several devices that are FDA-cleared for depression treatment. Furthermore, the evidence for tDCS, while promising, has primarily focused on MDD, with limited meta-analyses evaluating its role across a wider range of depressive disorders, particularly those with comorbid psychiatric or medical conditions.

Given the complexity of comorbid depression, where treatment response can differ from isolated MDD, we conducted a systematic review and meta-analysis to evaluate the role and safety of tES across these conditions. This study aims to inform clinical applications and guide future research on tES application in diverse depressive populations.

## Methods

This systematic review and meta-analysis was reported following the Preferred Reporting Items for Systematic Reviews and Meta-Analyses (PRISMA) 2020 guidelines.^8^ The study was registered with PROSPERO (CRD42023488253).

### Search Strategy and Selection Criteria

We performed a comprehensive search of several databases, including MEDLINE, Embase, Cochrane Central Register of Controlled Trials, Cochrane Database of Systematic Reviews, APA PsycINFO, and Scopus via Elsevier. The search was designed by a medical librarian and covered database inception to September 17, 2024 (eAppendix 1 in Supplement 1).

Inclusion criteria were (1) individuals with primary depression or comorbid depression, (2) tES modalities (tDCS, tACS, and tRNS) as independent interventions or in comparison with sham or other modalities, (3) reported depression assessment methods and outcomes, (4) randomized clinical trial (RCT) study design, and (5) studies published in English. Exclusion criteria were (1) participants younger than 18 years and (2) conference abstracts, secondary analyses, and nonhuman studies.

Articles were processed in Covidence. Duplicates were removed, and 2 reviewers (M.L. and S.K.) independently screened each article title and abstract. Full texts were reviewed separately by independent reviewers (R.B. and C.R.) and disagreements were resolved by a third reviewer (S.P.).

### Data Extraction and Quality Assessment

Data including patient characteristics, intervention details, stimulation parameters, depressive outcomes, adverse events (AEs), and follow-up information were collected. We evaluated risk of bias using the Cochrane Collaboration Risk of Bias 2 (ROB-2) tool^9^ for RCTs. Independent reviewers (R.B., C.R., and S.P.) conducted data extraction and quality assessment.

### Outcome Measures

The primary outcomes were depression severity, antidepressant response, and remission. Acceptability and tolerability were evaluated by dropout rate, dropout due to AEs, and AEs reported. Depression severity was measured using a standardized assessment tool. For studies using multiple depression measures, the most frequently used depression measure was used (eAppendix 2 in Supplement 1). Clinical response was defined as a reduction of 50% or more in depression scores from baseline. Remission was defined as Hamilton Depression Rating Scale score of 7 or less, Montgomery–Åsberg Depression Rating Scale score of 10 or less, or a Calgary Depression Scale for Schizophrenia score of 6 or less. We categorized AEs as mild to moderate or serious based on the classifications reported in the included studies. Serious AEs were defined as those resulting in psychiatric hospitalization, new-onset mania or hypomania, or attempted or completed suicides. Stimulation parameters provided in the included studies were also extracted and analyzed to identify associations with depression outcomes.

### Statistical Analyses

Statistical analyses were performed using the meta and metafor packages in R version 4.4.1 (R Project for Statistical Computing). Standardized mean differences (SMDs) were calculated using the Hedge g statistic for continuous outcomes, with effect sizes of 0.8, 0.5, and 0.2 considered large, moderate, and small, respectively.^10^ Odds ratios (ORs) were analyzed for categorical outcomes. Heterogeneity was assessed using the *I*^2^ statistic and categorized as low (0%-29%), moderate (30%-49%), substantial (50%-74%), or considerable (75%-100%).^11^ The DerSimonian and Laird random-effects model with Hartung-Knapp-Sidik-Jonkman variance adjustment was applied,^12^ and results were presented with 95% CIs and forest plots.

To explore sources of heterogeneity, univariate meta-regression models were performed to investigate the influence of baseline score and tES stimulation parameters on depression severity. Subgroup analysis for tDCS studies comparing electrode locations (frontal lobe [F]3 vs other regions), electrode size (25 cm^2^ vs 35 cm^2^), and length of follow-up (<3 months vs >3 months) were performed. Given the small number of comparison studies, subgroup analysis was not performed for tACS and tRNS studies.

To further test our findings, sensitivity analyses were conducted by excluding outlier studies (Hedges g ≤−1.5 or OR >5). Publication bias was evaluated with funnel plots and Egger regression tests, when more than 10 studies were included in the meta-analysis. For the Egger test, a 2-sided *P* < .05 was considered statistically significant.

The quality of evidence (QOE) was assessed using the Grading of Recommendations Assessment, Development, and Evaluation (GRADE) framework. GRADE categorizes evidence quality into 4 levels: high, moderate, low, and very low QOE.

## Results

**Figure 1.**
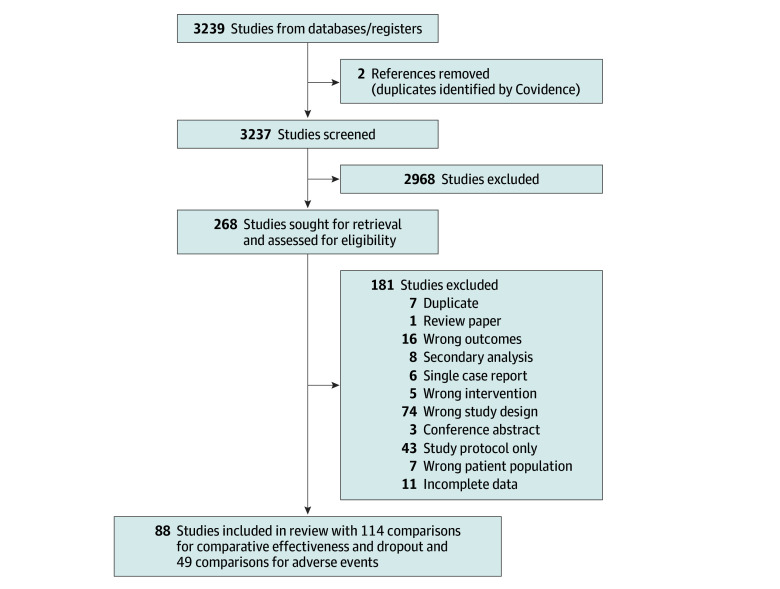
Flowchart of Studies

A total of 3237 abstracts were screened, of which 269 articles were identified for full-text review. Of these, 88 RCTs^13,14,15,16,17,18,19,20,21,22,23,24,25,26,27,28,29,30,31,32,33,34,35,36,37,38,39,40,41,42,43,44,45,46,47,48,49,50,51,52,53,54,55,56,57,58,59,60,61,62,63,64,65,66,67,68,69,70,71,72,73,74,75,76,77,78,79,80,81,82,83,84,85,86,87,88,89,90,91,92,93,94,95,96,97,98,99,100^ were included in the systematic review and meta-analysis.(Figure 1).

### Characteristics of Included RCTs

Detailed characteristics of the 88 RCTs^13,14,15,16,17,18,19,20,21,22,23,24,25,26,27,28,29,30,31,32,33,34,35,36,37,38,39,40,41,42,43,44,45,46,47,48,49,50,51,52,53,54,55,56,57,58,59,60,61,62,63,64,65,66,67,68,69,70,71,72,73,74,75,76,77,78,79,80,81,82,83,84,85,86,87,88,89,90,91,92,93,94,95,96,97,98,99,100^ were summarized (eTables 1-3 in Supplement 1). Of the 88 RCTs^13,14,15,16,17,18,19,20,21,22,23,24,25,26,27,28,29,30,31,32,33,34,35,36,37,38,39,40,41,42,43,44,45,46,47,48,49,50,51,52,53,54,55,56,57,58,59,60,61,62,63,64,65,66,67,68,69,70,71,72,73,74,75,76,77,78,79,80,81,82,83,84,85,86,87,88,89,90,91,92,93,94,95,96,97,98,99,100^ included, 25 RCTs^13,16,17,24,25,28,29,32,34,36,40,47,50,55,64,66,67,74,75,83,84,87,88,96,98^ had multiple treatment groups, yielding a total of 114 independent study groups analyzed. A total of 5522 patients were included; the mean (range) age was 43.1 (19.4-76.9) years, and 3198 (60.2%) were female. There were 104 study groups from 79 RCTs^13,15,16,17,18,19,20,21,22,23,24,25,26,27,28,29,30,31,32,34,35,36,38,39,40,41,42,43,44,45,46,47,48,49,50,51,52,53,54,55,56,57,58,59,60,61,62,63,64,65,66,67,69,70,71,72,73,74,75,77,78,79,80,81,83,84,85,86,87,88,89,90,91,92,94,95,96,97,100^ (91.2%) that evaluated tDCS, 7 study groups from 6 RCTs ^14,37,76,93,98,99^ (6.1%) that evaluated tACS, and 3 study groups from 3 RCTs^33,68,82^ (2.7%) that assessed tRNS. Of all RCTs, there were 61 study groups from 44 RCTs^14,17,19,21,22,23,24,25,27,28,29,30,32,34,37,39,40,45,47,48,50,54,55,56,60,61,62,67,68,69,70,72,73,75,79,82,83,85,87,91,93,95,99,100^ (53.5%) on MDD, 29 study groups from 23 RCTs^13,20,35,36,42,49,51,52,58,64,65,66,71,76,80,81,84,88,90,92,94,97,98^ (24.8%) on DPC, and 24 study groups from 21 RCTs^15,16,18,26,31,33,38,41,43,44,46,53,57,59,63,74,77,78,86,89,96^ (20.5%) on DMC. The conditions of DPC and DMC are listed in eTable 2 in Supplement 1. The most frequently used tES stimulation parameters were anodal F3 electrode placement (85 study groups [74.6%]), 35 cm^2^ electrode size (54 study groups [45.6%]), 2 mA current intensity (99 study groups [86.8%]), 20 minutes session duration (54 study groups [47.4%]), and a total number of 10 sessions (44 study groups [38.6%]).

### Risk of Bias

The risk of bias assessment demonstrated some variability across different domains (eFigure in Supplement 1). Potential publication bias was observed for tES vs sham or no treatment (t_71_ = −3.24; *P* = .002) or tDCS vs sham or no treatment (t_61_ = −3.59; *P* < .001) across all patients in studies of patients with DMC (tES vs sham or no treatment: t_18_ = −3.11; *P* = .006; tDCS vs sham or no treatment: t_18_ = −3.11; *P* = .007), and for response rate in tES vs sham among MDD studies (t_20_ = −2.37; *P* = .03) (eTables 4-5 in Supplement 1). No publication bias was detected for other response rates or remission rates across diagnostic groups.

### tES Compared With Sham or No Treatment Group

**Figure 2.**
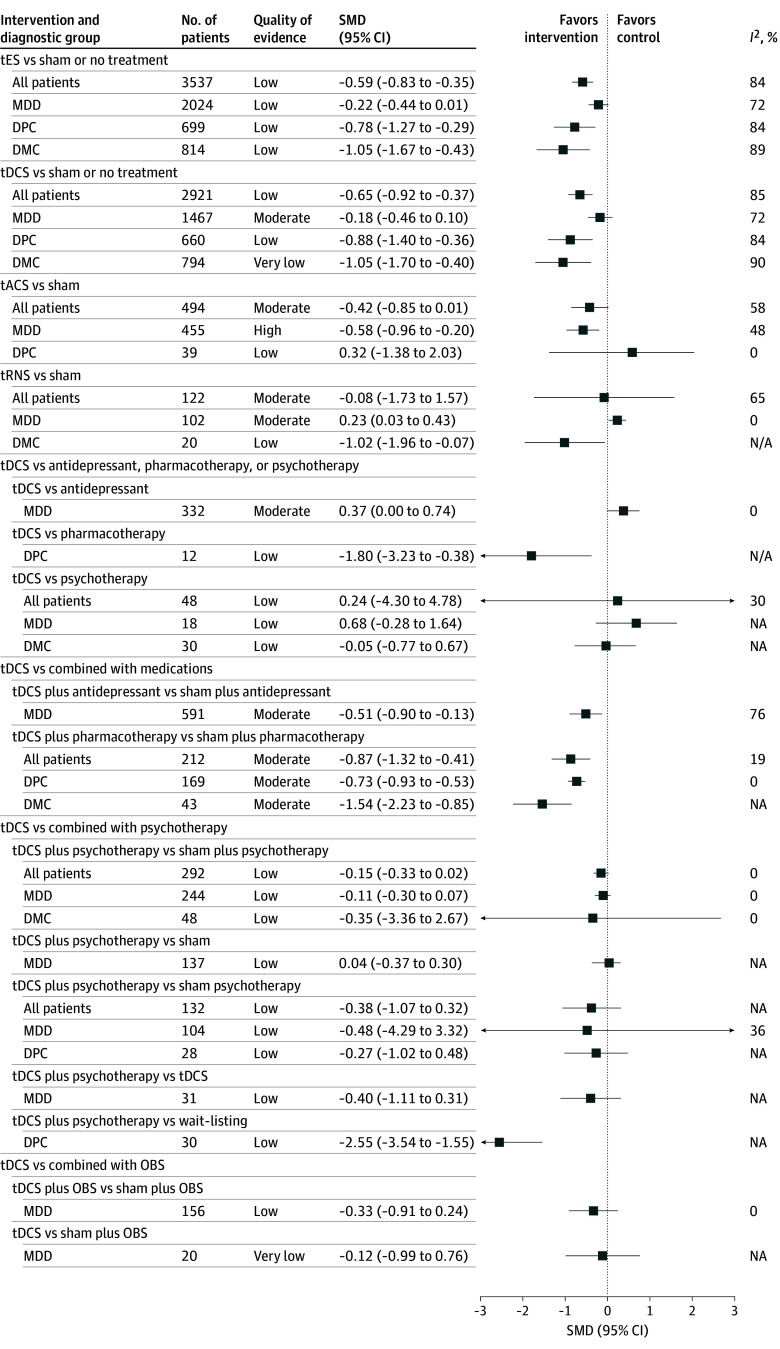
Pooled Effect Size and Quality of Evidence for Primary Depression Symptoms No studies were available for the following comparisons: (1) transcranial alternating current stimulation (tACS) vs sham in the depression with medical comorbidities (DMC) group and (2) transcranial random noise stimulation (tRNS) vs sham in the depression with psychiatric comorbidities (DPC) group. Studies included are listed in eTable 4 in Supplement 1. Other brain stimulation (OBS) techniques include electroconvulsive therapy, cranial electrotherapy stimulation, and repetitive transcranial magnetic stimulation. Pharmacotherapy indicates treatment involving additional medications (eg, methadone or naltrexone). MDD indicates major depressive disorder; NA, not applicable; SMD, standard mean difference; tDCS, transcranial direct current stimulation; tES, transcranial electrical stimulation.

**Figure 3.**
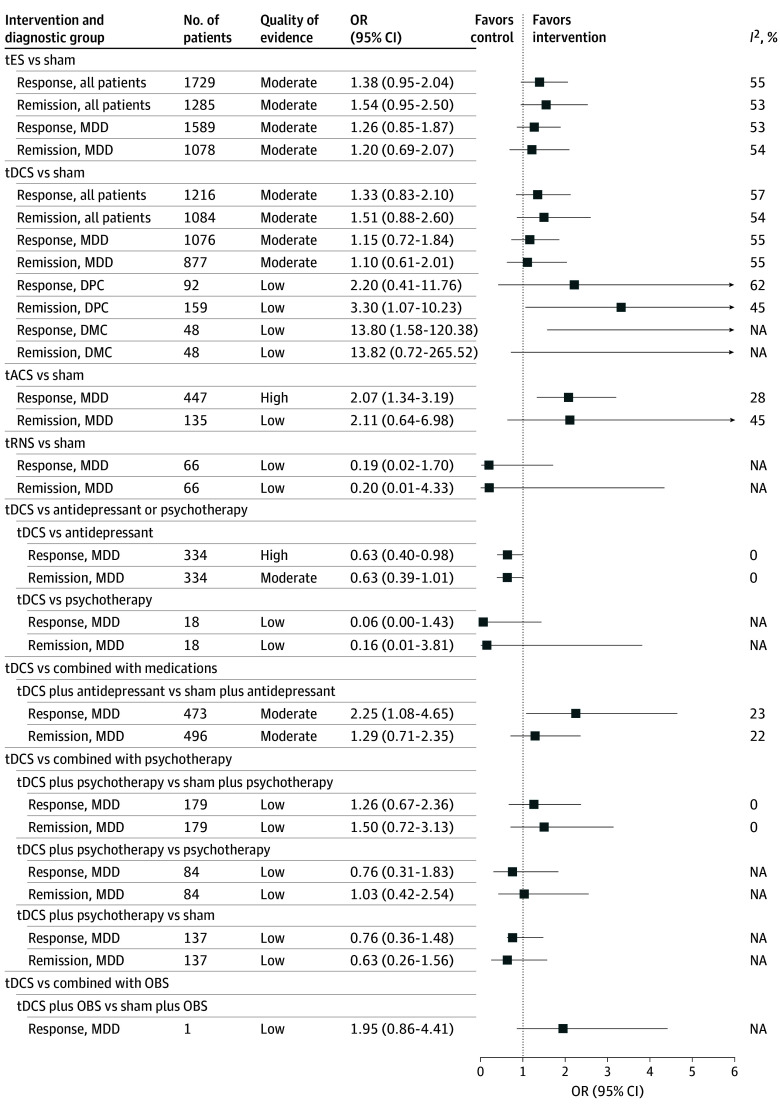
Pooled Effect Size and Quality of Evidence for Treatment Response and Remission Studies included are listed in eTable 5 in Supplement 1. Other brain stimulation (OBS) techniques include electroconvulsive therapy, cranial electrotherapy stimulation, and repetitive transcranial magnetic stimulation. DMC indicates depression with medical comorbidities; DPC, depression with psychiatric comorbidities; MDD, major depressive disorder; NA, not applicable; OR, odds ratio; tACS, transcranial alternating current stimulation; tDCS, transcranial direct current stimulation; tES, transcranial electrical stimulation; tRNS, transcranial random noise stimulation.

**Figure 4.**
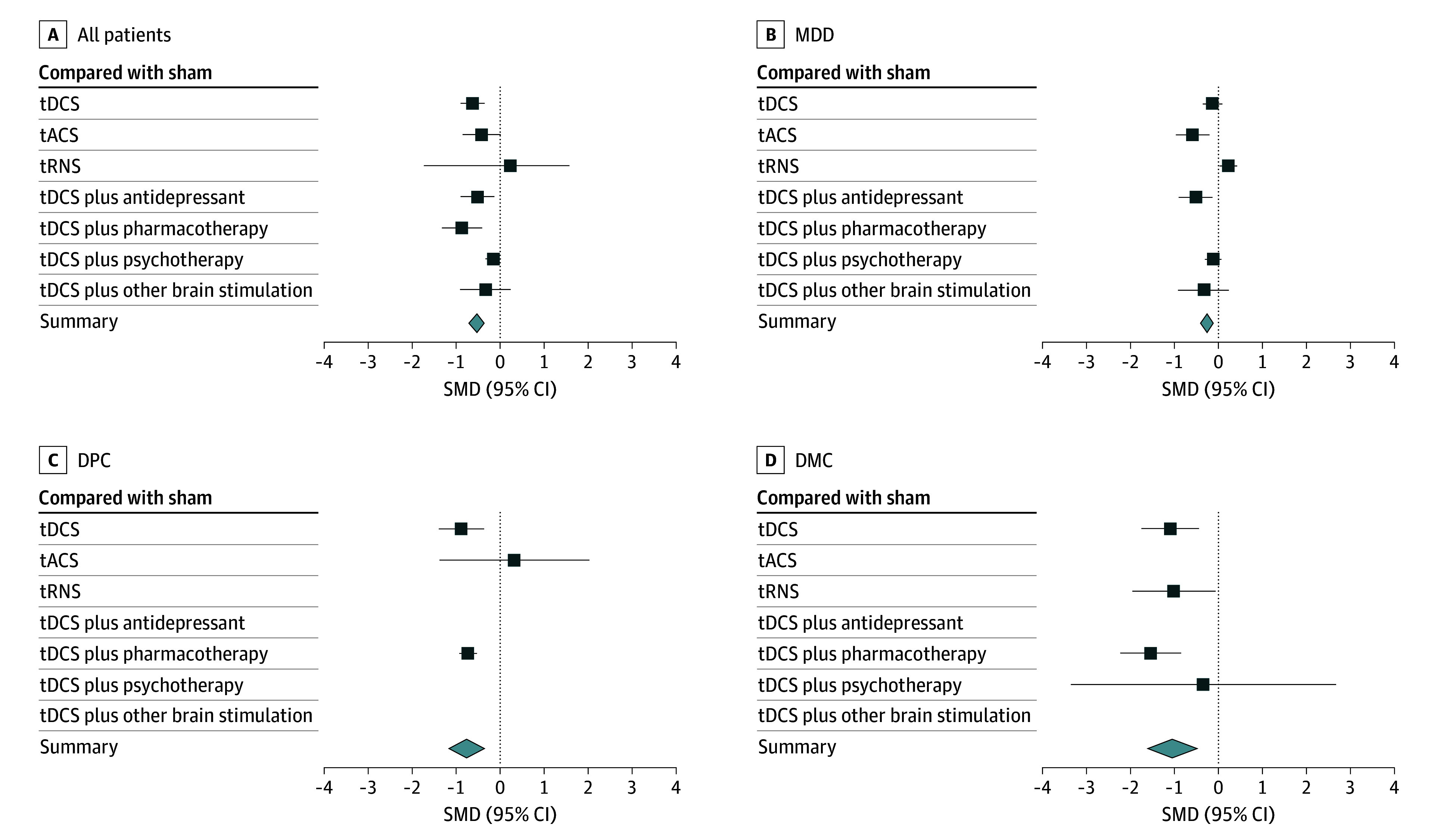
Transcranial Electrical Stimulation Compared With Sham Across Diagnostic Groups Standardized mean differences (SMDs) and 95% CIs are reported in eTable 4 in Supplement 1. DMC indicates depression with medical comorbidities; DPC, depression with psychiatric comorbidities; MDD, major depressive disorder; tACS, transcranial alternating current stimulation; tDCS, transcranial direct current stimulation; tRNS, transcranial random noise stimulation.

The pooled effect size and QOE for depression (Figure 2) and treatment response and remission (Figure 3) were summarized. Across all diagnostic groups, tES was associated with a moderate pooled effect size for depressive symptoms (SMD = −0.59; 95% CI: −0.83 to −0.35; *I*^2^ = 84%; low QOE). There was a modest, although not statistically significant, increase in response rates (OR, 1.38; 95% CI, 0.95 to 2.04; *I*^2^ = 55%; moderate QOE) compared with sham or no treatment. The tES treatment was associated with a higher improvement in depressive symptoms among patients with DMC (SMD = −1.05; 95% CI, −1.67 to −0.43; *I*^2^ = 89%; low QOE) and DPC (SMD = −0.78; 95% CI, −1.27 to −0.29; *I*^2^ = 84%; low QOE) compared with MDD (SMD = −0.22; 95% CI, −0.44 to 0.01; *I*^2^ = 72%; low QOE) (Figure 2). Similarly, a small but significant effect size was observed for MDD compared with the effect size among DMC and DPC. (Figure 4). No significant differences were observed in remission rates across diagnostic groups.

### tDCS, tACS, and tRNS vs Sham or No Treatment, Medication, or Psychotherapy

Among tES modalities, tDCS was associated with a significant improvement in depressive symptoms among patients with DMC (SMD = −1.05; 95% CI, −1.70 to −0.40; *I*^2^ = 90%; very low QOE) and DPC (SMD = −0.88, 95% CI, −1.40 to −0.36; *I*^2^ = 84%, low QOE). However, no significant improvement was observed in patients with MDD (SMD = −0.18; 95% CI, −0.46 to 0.10; *I*^2^ = 72%; moderate QOE) (Figure 2). tACS was associated with a higher reduction of depressive symptoms in patients with MDD (SMD = −0.58; 95% CI, −0.96 to −0.20; *I*^2^ = 49%; high QOE) and a higher likelihood of increased response (OR, 2.07; 95% CI, 1.34 to 3.19; *I*^2^ = 28%; high QOE). However, tRNS did not demonstrate significant improvements in depression, response rates, or remission rates across diagnostic groups (Figure 2, Figure 3).

Antidepressant treatments alone were associated with higher improvements in depressive outcomes (SMD = 0.37; 95% CI, 0.00 to 0.74) and response rates (OR, 0.63; 95% CI, 0.40 to 0.98) compared with tDCS for MDD. However, tDCS was associated with a significant reduction in depressive symptoms among patients with DPC when compared with medications alone (SMD = −1.80; 95% CI, −3.23 to −0.38). tDCS did not have significant effects when compared with psychotherapy (Figure 2 and Figure 3).

### tDCS Combined With Medication

tDCS combined with antidepressant was associated with a significant reduction in depressive symptoms among patients with MDD (SMD = −0.51; 95% CI, −0.90 to −0.13; *I*^2^ = 76%; moderate QOE) and improved response rate (OR, 2.25; 95% CI, 1.08 to 4.65; *I*^2^ = 23%; high QOE), while tDCS alone did not yield a significant improvement. tDCS combined with pharmacotherapy also showed large improvement in depressive symptoms among both DMC and DPC groups (Figure 2 and Figure 3).

### tDCS Combined With Psychotherapy

tDCS combined with psychotherapy did not reveal any improvements across all patients when compared with sham tDCS combined with psychotherapy, sham tDCS, psychotherapy alone, or tDCS alone. However, in patients with DPC, this combination showed a large effect size (SMD = −2.55; 95% CI, −3.54 to −1.55; low QOE) compared with a waitlisting control group.

### tDCS Combined With Other Brain Stimulations

There were 3 RCTs^34,50,60^ comprising a total of 156 patients. No significant difference was observed between tDCS combined with other brain stimulations (electroconvulsive therapy, repetitive transcranial magnetic stimulation, or cranial electrotherapy stimulation) and sham tDCS combined with other brain stimulations.

### Acceptability and AEs

The overall dropout rates for the active (2831 participants) and sham groups (2733 participants) were 9.4% (266 participants) and 9.0% (245 participants), respectively, with dropout due to AEs being 0.6% (16 participants) in the active group and 0.4% (11 participants) in the sham group. The difference in dropout rates and dropout due to AEs between the 2 groups was not statistically significant (eTable 6 in Supplement 1). Symptoms leading to withdrawals due to AEs included transient hypomania,^22,29,54^ suicidal ideation,^29^ prolonged headaches,^40^ and scalp discomfort.^23^

AEs were reported in 47 RCTs.^13,14,17,19,20,22,23,25,26,28,29,30,32,34,35,37,40,41,43,47,48,49,50,53,54,55,56,63,67,68,70,71,72,73,74,75,77,78,81,87,89,92,93,95,96,97,99^ Mild to moderate AEs were more frequent with tES, specifically in tDCS, tACS, and tRNS compared with sham (eTable 6 in Supplement 1). Serious AEs were observed more frequently in tDCS than sham (OR, 1.91; 95% CI, 1.25-2.93; *I*^2^* *= 0%). Among serious AEs, hypomania or mania were most common, with 15 episodes in treatment groups compared with 3 episodes in control groups. No studies reported serious AEs as seizures. Frequently reported mild to moderate AEs included skin redness, headaches, itching, tingling, burning sensations at the electrode site, dizziness, nausea, and sleep disturbances (eTable 6 in Supplement 1).

### tDCS Treatment Stimulation Parameters

Following the evaluation of the overall role and safety of tES, we analyzed the stimulation parameters. The univariate meta-regression analyses identified electrode size and electrode placement, for MDD and DPC groups, as significant factors associated with depressive outcomes in tDCS treatment (Table). Baseline depression score, stimulation duration, number of sessions, and current intensity were significant factors associated with depressive outcomes in DMC group undergoing tDCS treatment (Table).

**Table.** Univariate Meta-Regression Results of Transcranial Direct Current Stimulation in Diagnostic Groups

Variable	Major depressive disorder		Depression with psychiatric comorbidities		Depression with medical comorbidities	
	Coefficient (SE)	*P* value	Coefficient (SE)	*P* value	Coefficient (SE)	*P* value
Baseline score	−0.00 (0.01)	.73	−0.02 (0.02)	.29	−0.07 (0.03)	.05
Electrode size	0.04 (0.01)	.01	0.07 (0.02)	.005	0.01 (0.05)	.80
Current intensity	0.16 (0.22)	.47	2.86 (1.92)	.15	−0.96 (0.26)	.001
Stimulation duration	0.01 (0.01)	.26	0.02 (0.04)	.57	−0.14 (0.05)	.01
No. of sessions	0.02 (0.01)	.18	0.02 (0.03)	.48	−0.12 (0.04)	.01
Electrode placements (F3 or other regions)	0.78(0.35)	.03	0.08 (0.60)	.90	0.95 (0.50)	.07
Specific electrode placements^a^						
Anode F3, cathode F4	−0.07 (0.61)	.91	1.13 (0.63)	.09	0.82 (1.45)	.58
Anode F3, cathode F8	−0.13 (0.63)	.83	2.28 (0.83)	.01	NA	NA
Anode F3, cathode Fp2	−0.01 (0.61)	.99	1.59 (0.67)	.03	−1.27 (1.54)	.42
Anode Fp1, cathode Fp2	0.75 (0.80)	.36	NA	NA	NA	NA
Anode Fp2, cathode F4	1.29 (0.82)	.13	NA	NA	NA	NA
Anode F3, cathode extracephalic regions	NA	NA	0.79	.45	NA	NA
Anode F3, cathode T3	NA	NA	NA	NA	NA	NA
Anode F3, cathode Fp1, Fz, C3, and F7	NA	NA	−2.86 (0.95)	.007	NA	NA
Anode F4, cathode Fp1	NA	NA	1.09 (0.94)	.26	NA	NA
Anode C3, cathode F4	NA	NA	NA	NA	NA	NA
Anode C3, cathode Fp2	NA	NA	NA	NA	−0.05 (1.50)	.97
Anode C3, cathode extracephalic regions	NA	NA	NA	NA	−0.45 (1.37)	.75
Anode Fz, cathode Iz	NA	NA	NA	NA	0.31 (1.95)	.87
Anode other regions (occipital or temporal)	−0.10 (0.89)	.91	NA	NA	−0.28 (1.92)	.88
Cathode F3	NA	NA	0.56 (0.65)	.40	NA	NA
Cathode F4	NA	NA	0.78 (0.74)	.31	NA	NA
Cathode FC1	NA	NA	NA	NA	0.78 (1.96)	.70

Abbreviations: C, central; F, frontal; Fp, prefrontal; NA, data not available for this configuration or group; T, temporal; z, zero.

^a^ Placement of scalp electrodes according to the international 10-20 system for electroencephalograpy. Z sites refer to an electrode placed on the midline sagittal plane of the skull.

### Sensitivity and Subgroup Analyses

Sensitivity analyses confirmed the robustness of the combined effect size by excluding outlier (exceptionally large effect size) studies (eTables 7-8 in Supplement 1). Most of these studies involved patients with comorbidities, and only 1 RCT^67^ compared tDCS with no treatment in patients with MDD.

Subgroup analyses were summarized in eTables 9 to 13 in Supplement 1. Comparing electrode sizes, larger improvements in depression among patients with MDD were associated with the 25 cm^2^ electrode compared with the 35 cm^2^ (eTable 11 in Supplement 1). Anodal tDCS targeting the left dorsolateral prefrontal cortex (DLPFC; F3) showed a significant effect size for MDD compared with sham and other tDCS stimulation sites (eTable 12 in Supplement 1). In comparisons between tES and sham, postintervention results were significantly higher than those observed at less than 3 months follow-up (eTable 9 in Supplement 1). tDCS combined with antidepressant and tACS treatment still had significant effect sizes at follow-up less than 3 months (eTable 9 in Supplement 1) and more than 3 months (eTable 10 in Supplement 1). No significant differences in outcomes were found based on follow-ups longer than 3 months across all diagnostic groups. Notably, when comparing tDCS treatment with sham, home-based tDCS demonstrated no significant effect size whereas clinic-based tDCS showed a significant effect size (eTable 13 in Supplement 1).

## Discussion

We conducted a systematic review and meta-analysis evaluating different tES modalities in treating depressive disorders. Our findings indicate that to date tDCS and tACS, but not tRNS, are associated with improvement in MDD and DPC or DMC. AEs with tES were generally mild to moderate, but more frequent compared with sham groups. These results support the clinical utility of tES as a treatment option for depressive disorders. tES portability, simplicity, affordability, and minimal AEs make it appealing for clinical practice consideration.

Specifically, tDCS showed greater improvements in the comorbid depression group than MDD alone. Combining tDCS with medications, such as antidepressants or other psychotropic drugs, resulted in improved outcomes for MDD and DPC compared with tDCS monotherapy. Electrode size and placement were significantly associated with outcomes. Anodal left DLPFC tDCS yielded significant effect sizes for MDD, compared with other brain regions. Home-based tDCS was not associated with reduced depression compared with sham. tACS was associated with improvement in MDD but was limited by the smaller number of studies and sample sizes. In contrast, tRNS was not associated with improvement in depression outcomes, suggesting it may not address the underlying pathophysiology of depression, but it was also limited by the smaller number of studies and sample sizes.

This analysis adds to prior meta-analyses^4,5,101,102,103,104,105,106,107^ on noninvasive brain stimulation by focusing on tES treatments for depressive disorders with and without comorbidities. Our results align with previous meta-analyses on tDCS and tACS for MDD,^5,7,107^ confirming their findings through sensitivity analyses.

Notably, this study addresses a critical gap by demonstrating that individuals with comorbid conditions benefit more from tDCS treatment than those with MDD alone. This finding aligns with meta-analyses on tDCS for poststroke depression and bipolar depression.^104,108,109,110^ Similar to prior findings, our analysis noted that tDCS combined with medication was associated with improvement in MDD compared with tDCS monotherapy.^5^ This finding contrasts with other meta-analyses that grouped all tDCS studies and reported a moderate effect size for MDD.^101,102,103,106,107^ Our subgroup analysis highlighted that anodal tDCS targeting the left F3 region was associated with improvement in MDD.

Our analysis of tDCS combined with other therapies is consistent with prior findings.^5,111^ Specifically, combining tDCS with pharmacotherapy was associated with reduced depressive symptoms across diagnostic groups and improved treatment response for MDD compared with tDCS monotherapy. This finding aligns with previous meta-analyses on tDCS for MDD^5,111^ but contrasts with another study.^107^ Furthermore, combining tDCS with psychotherapy or other brain stimulation techniques did not note improvement in depression compared with sham tDCS with psychotherapy, sham alone, or standalone psychotherapy. These results challenge the assumption that combining tDCS with psychotherapy might not correlate with further improvement.

Significant heterogeneity in tES treatment parameters, particularly tDCS, is commonly observed in meta-analyses on depression. Our analysis of tDCS montage parameters revealed that electrode size and electrode placement were critical contributors to variability. Specifically, smaller electrodes were associated with more favorable outcomes, and 1 RCT^97^ demonstrated a large effect size using high-definition tDCS. Additionally, our analysis highlighted that targeting the left DLPFC (F3) is a preferred option for depressive disorders, consistent with a dose-response meta-analysis in treatment-resistant depression.^112^

This review also provided a comprehensive evaluation of AEs associated with tES, indicating that AEs are generally mild to moderate and rarely serious. Serious AEs were rare and occurred primarily with tDCS studies, often when combined with antidepressants. These findings align with prior evidence suggesting that active tES, particularly tDCS, is safe (although mild to moderate AEs are more common) and well-tolerated compared with sham.^113,114^ This potential for AEs underscores the importance of monitoring for hypomanic or manic episodes, especially when tDCS is combined with pharmacotherapy.

### Limitations

This study has several limitations. First, while substantial evidence exists for tDCS in depression, published data on tACS and tRNS remain limited, meaning our findings primarily reflect tDCS studies. The small number of tACS and tRNS studies limited the potential to conduct a robust subgroup analysis for these tES modalities. Additionally, our categorization of tDCS studies into monotherapy and combined therapy could be confounded if medication use was unspecified. Moreover, stratification by depression severity was not possible, restricting our insights into how severity impacts treatment outcomes. A comprehensive safety profile was limited because more than 40% of included studies did not report AEs. Further, we observed potential publication bias in tDCS and sham comparisons, particularly among patients with DMC. Due to small study number, publication bias could not be statistically assessed for other comparisons.

## Conclusions

In this systematic review and meta-analysis, we found that tDCS combined with medication was associated with reduced depressive symptoms among patients with MDD and comorbid depression. When tDCS monotherapy was compared with sham, patients with comorbid depression had higher improvement in depressive symptoms than those with MDD alone. tACS was associated with improvements in some depressive symptoms among patients with MDD, but further studies are needed to confirm these results. Overall, these findings suggest that tES is well-tolerated, associated with mild to moderate AEs, and poses a minimal risk of serious AEs. Future research should study ideal stimulation parameters and individualize tES interventions, validate specialized modalities such as multifocal tES or high-definition tDCS, and further investigate tACS for depression.

## Notes

Supplement 1. **eAppendix 1.** Search Strategies **eAppendix 2.** Algorithm for Selecting One Depression Measure for Each Study **eTable 1.** Characteristics of the Included Studies by Diagnostic Groups **eTable 2.** Characteristics of Comorbid Depression **eTable 3.** Summary of tES Studies Across Diagnostic Groups **eFigure.** Risk of Bias Assessment for Randomized Control Trials **eTable 4.** Pooled Effect Size and Quality of Evidence for Primary Depression Symptoms **eTable 5.** Pooled Effect Size and Quality of Evidence for Treatment Response and Remission **eTable 6.** Pooled Effect Size and Quality of Evidence for Adverse Events (Including Dropouts, Dropouts Due To Any Adverse Events, and Adverse Events) **eTable 7.** Sensitivity Analysis for Primary Depression Symptoms: Exclusion of Outlier Studies (Hedges' g ≥ 1.5) **eTable 8.** Sensitivity Analysis for Response and Remission: Exclusion of Outlier Studies (OR > 5) **eTable 9.** Subgroup analysis: follow-up less than 3 months **eTable 10.** Subgroup Analysis: Follow-Up More Than 3 Months **eTable 11.** Subgroup Analysis: Electrode Size **eTable 12.** Subgroup Analysis: Electrode Locations **eTable 13.** Subgroup Analysis of tDCS Studies: Treatment Settings
eReferences.

Supplement 2.
Data Sharing Statement
