# Supplementary material for: Transcranial Electrical Stimulation in Treatment of Depression: A Systematic Review and Meta-Analysis
Source: JAMA Netw Open. 2025 Jun 18;8(6):e2516459. doi: 10.1001/jamanetworkopen.2025.16459 (PMC12177679; doi:10.1001/jamanetworkopen.2025.16459)
Supplement: Supplement 1. — eAppendix 1. Search Strategies eAppendix 2. Algorithm for Selecting One Depression Measure for Each Study eTable 1. Characteristics of the Included Studies by Diagnostic Groups eTable 2. Characteristics of Comorbid Depression eTable 3. Summary of tES Studies Across Diagnostic Groups eFigure. Risk of Bias Assessment for Randomized Control Trials eTable 4. Pooled Effect Size and Quality of Evidence for Primary Depression Symptoms eTable 5. Pooled Effect Size and Quality of Evidence for Treatment Response and Remission eTable 6. Pooled Effect Size and Quality of Evidence for Adverse Events (Including Dropouts, Dropouts Due To Any Adverse Events, and Adverse Events) eTable 7. Sensitivity Analysis for Primary Depression Symptoms: Exclusion of Outlier Studies (Hedges' g ≥ 1.5) eTable 8. Sensitivity Analysis for Response and Remission: Exclusion of Outlier Studies (OR > 5) eTable 9. Subgroup analysis: follow-up less than 3 months eTable 10. Subgroup Analysis: Follow-Up More Than 3 Months eTable 11. Subgroup Analysis: Electrode Size eTable 12. Subgroup Analysis: Electrode Locations eTable 13. Subgroup Analysis of tDCS Studies: Treatment Settings eReferences. [file jamanetwopen-e2516459-s001.pdf]

Supplemental Online Content

Ren C, Pagali SR, Wang Z, et al. Transcranial electrical stimulation in the treatment of depression: a systematic review and meta-analysis. *JAMA Netw. Open*. 2025;8(6):e2516459. doi:10.1001/jamanetworkopen.2025.16459

eAppendix 1. Search Strategies

eAppendix 2. Algorithm for Selecting One Depression Measure for Each Study

eTable 1. Characteristics of the Included Studies by Diagnostic Groups

eTable 2. Characteristics of Comorbid Depression

eTable 3. Summary of tES Studies Across Diagnostic Groups

eFigure. Risk of Bias Assessment for Randomized Control Trials

eTable 4. Pooled Effect Size and Quality of Evidence for Primary Depression Symptoms

eTable 5. Pooled Effect Size and Quality of Evidence for Treatment Response and Remission

eTable 6. Pooled Effect Size and Quality of Evidence for Adverse Events (Including Dropouts, Dropouts Due To Any Adverse Events, and Adverse Events)

eTable 7. Sensitivity Analysis for Primary Depression Symptoms: Exclusion of Outlier Studies (Hedges' g ≥ 1.5)

eTable 8. Sensitivity Analysis for Response and Remission: Exclusion of Outlier Studies (OR > 5)

eTable 9. Subgroup analysis: follow-up less than 3 months

eTable 10. Subgroup Analysis: Follow-Up More Than 3 Months

eTable 11. Subgroup Analysis: Electrode Size

eTable 12. Subgroup Analysis: Electrode Locations

eTable 13. Subgroup Analysis of tDCS Studies: Treatment Settings

eReferences.

This supplemental material has been provided by the authors to give readers additional information about their work.

© 2025 Ren C et al. *JAMA Network Open*.

eAppendix 1. Search strategies

OVID

Database(s): **Ovid MEDLINE(R) 1946 to Present and Epub Ahead of Print, In-Process & Other Non-Indexed Citations and Ovid MEDLINE(R) Daily, APA PsycInfo** 1987+**, EBM Reviews - Cochrane Central Register of Controlled Trials, EBM Reviews - Cochrane Database of Systematic Reviews** 2005+, **Embase** 1974+

#	Searches
1	exp Mood Disorders/
2	(depression or depressive or bipolar or anxiety or "panic disorder*" or "obsessive-compulsive" or dysthymi* or cyclothymi* or hyperthymi* or mania or manic or hypomani* or "post-traumatic-stress-disorder" or PTSD).ti,ab,hw,kf,tw.
3	(mood* adj2 (dysregulat* or dysfunction or disruptive or disorder*)).ti,ab,hw,kf,tw. or mood.ti.
4	1 or 2 or 3
5	(tES or "transcranial electric stimulation" or "transcranial direct stimulation" or tDCS or "transcranial alternating current stimulation" or tACS or "transcranial random noise stimulation" or tRNS).ti,ab,hw,kf.tw.
6	Transcranial Direct Current Stimulation/
7	exp transcranial electrical stimulation/
8	5 or 6 or 7
9	4 and 8
10	(exp animals/ or exp nonhuman/) not exp humans/
11	((alpaca or alpacas or algae* or amphibian or amphibians or animal or animals or antelope or armadillo or armadillos or avian or baboon or baboons or bats or beagle or

© 2025 Ren C et al. *JAMA Network Open*.

	beagles or bee or bees or bird or birds or bison or bovine or buffalo or buffaloes or buffalos or "c elegans" or "Caenorhabditis elegans" or camel or camels or canine or canines or canis or carp or cats or catfish or cattle or chamaeleo* or chameleon* or chick or chicken or chickens or chicks or chimp or chimpanze or chimpanzees or chimps or cow or cows or "D melanogaster" or "dairy calf" or "dairy calves" or deer or dog or dogs or donkey or donkeys or drosophila or "Drosophila melanogaster" or duck or duckling or ducklings or ducks or equid or equids or equine or equines or feline or felines or ferret or ferrets or finch or finches or fish or flatworm or flatworms or fox or foxes or frog or frogs or "fruit flies" or "fruit fly" or "G mellonella" or "Galleria mellonella" or geese or gerbil or gerbils or goat or goats or goose or gorilla or gorillas or groundhog or groundhogs or hamster or hamsters or hare or hares or heifer or heifers or horse or horses or iguana or iguanas or insect or insects or jellyfish or kangaroo or kangaroos or kitten or kittens or "laboratory animal*" or lagomorph or lagomorphs or lamb or lambs or lemur or lemurs or lemuridae or llama or llamas or macaque or macaques or macaw or macaws or marmoset or marmosets or mice or minipig or minipigs or mink or minks or monkey or monkeys or mouse or mule or mules or muskrat or muskrats or nematode or nematodes or newt or newts or octopus or octopuses or orangutan or "orang-utan" or orangutans or "orang-utans" or oxen or parrot or parrots or pig or pigeon or pigeons or piglet or piglets or pigs or porcine or primate or primates or poultry or quail or rabbit or rabbits or rat or rats or reptile or reptiles or rodent or rodents or ruminant or ruminants or salmon or sheep or shrimp or slug or slugs or swine or tamarin or tamarins or tilapia or tilapias or toad or toads or trout or urchin or urchins or vole or voles or waxworm or waxworms or weasel or weasels or wolf or wolves or worm or worms or wrass* or xenopus or "zebra fish" or zebrafish) not (human or humans or patient or patients)).ti,ab,hw,kw.

© 2025 Ren C et al. *JAMA Network Open*.

12	(rat or rats or mice or mouse or murine or pig or pigs or porcine or swine or dog or dogs).ti.
13	or/10-12
14	9 not 13
15	(conference abstract or conference review or editorial or erratum or note or addresses or autobiography or bibliography or biography or blogs or comment or dictionary or directory or interactive tutorial or lectures or legal cases or legislation or news or newspaper article or patient education handout or periodical index or portraits or published erratum or video-audio media or webcasts).mp. or conference abstract.st.
16	14 not 15
17	limit 16 to yr="2003 -Current"
18	limit 17 to english language [Limit not valid in CDSR; records were retained]
19	remove duplicates from 18

SCOPUS

1	TITLE ((depression OR depressive OR bipolar OR anxiety OR "panic disorder*" OR "obsessive-compulsive" OR dysthymi* OR cyclothymi* OR hyperthymi* OR mania OR manic OR hypomani* OR "post-traumatic-stress-disorder" OR ptsd))
2	TITLE ((mood* W/2 (dysregulat* OR dysfunction OR disruptive OR disorder*)))
3	1 or 2
4	TITLE-ABS-KEY ((tes OR "transcranial electric stimulation" OR "transcranial direct stimulation" OR tdcs OR "transcranial alternating current stimulation" OR tacs OR "transcranial random noise stimulation" OR trns))

© 2025 Ren C et al. *JAMA Network Open*.

5	3 and 4
6	INDEX(embase) OR INDEX(medline) OR PMID(0* OR 1* OR 2* OR 3* OR 4* OR 5* OR 6* OR 7* OR 8* OR 9*)
7	5 not 6
8	(TITLE-ABS-KEY ((alpaca OR alpacas OR amphibian OR amphibians OR animal OR animals OR antelope OR armadillo OR armadillos OR avian OR baboon OR baboons OR beagle OR beagles OR bee OR bees OR bird OR birds OR bison OR bovine OR buffalo OR buffaloes OR buffalos OR "c elegans" OR "Caenorhabditis elegans" OR camel OR camels OR canine OR canines OR carp OR cats OR cattle OR chick OR chicken OR chickens OR chicks OR chimp OR chimpanze OR chimpanzees OR chimps OR cow OR cows OR "D melanogaster" OR "dairy calf" OR "dairy calves" OR deer OR dog OR dogs OR donkey OR donkeys OR drosophila OR "Drosophila melanogaster" OR duck OR duckling OR ducklings OR ducks OR equid OR equids OR equine OR equines OR feline OR felines OR ferret OR ferrets OR finch OR finches OR fish OR flatworm OR flatworms OR fox OR foxes OR frog OR frogs OR "fruit flies" OR "fruit fly" OR "G mellonella" OR "Galleria mellonella" OR geese OR gerbil OR gerbils OR goat OR goats OR goose OR gorilla OR gorillas OR hamster OR hamsters OR hare OR hares OR heifer OR heifers OR horse OR horses OR insect OR insects OR jellyfish OR kangaroo OR kangaroos OR kitten OR kittens OR lagomorph OR lagomorphs OR lamb OR lambs OR llama OR llamas OR macaque OR macaques OR macaw OR

© 2025 Ren C et al. *JAMA Network Open*.

	macaws OR marmoset OR marmosets OR mice OR minipig OR minipigs OR mink OR minks OR monkey OR monkeys OR mouse OR mule OR mules OR nematode OR nematodes OR octopus OR octopuses OR orangutan OR "orang-utan" OR orangutans OR "orang-utans" OR oxen OR parrot OR parrots OR pig OR pigeon OR pigeons OR piglet OR piglets OR pigs OR porcine OR primate OR primates OR quail OR rabbit OR rabbits OR rat OR rats OR reptile OR reptiles OR rodent OR rodents OR ruminant OR ruminants OR salmon OR sheep OR shrimp OR slug OR slugs OR swine OR tamarin OR tamarins OR toad OR toads OR trout OR urchin OR urchins OR vole OR voles OR waxworm OR waxworms OR worm OR worms OR xenopus OR "zebra fish" OR zebrafish) AND NOT (human OR humans OR patient OR patients)))
9	7 not 8
10	LIMIT-TO (LANGUAGE , "English") AND LIMIT-TO (DOCTYPE , "ar") AND LIMIT-TO (SRCTYPE , "j")

© 2025 Ren C et al. *JAMA Network Open*.

eAppendix 2. Algorithm for selecting one depression measure for each study

The algorithm used the following priorities:

1. Hamilton Depression Rating Score (Ham-D) (N = 44).

2. Beck Depression Inventor- II (BDI-II) (N = 42).

3. Montgomery–Åsberg Depression Rating Scale (MADRS) (N = 29).

4. Patient Health Questionnaire (PHQ-9) (N = 3).

5. Depression Anxiety Stress Scales-21 (DASS-21) (N =4).

6. Calgary Depression Scale for Schizophrenia (CDSS) (N=3).

7. Quick Inventory of Depressive Symptomatology (QIDS-SR) (N=6).

8. Positive and Negative Syndrome Scale (PANSS) depression subscale (N=1).

N indicates numbers of studies.

© 2025 Ren C et al. *JAMA Network Open*.

eTable 1. Characteristics of the included studies by diagnostic groups

Characteristic	All patients	MDD	Depression in Psychiatric Comorbidities	Depression in Medical Comorbidities
**No. of tES studies (Sample size)**	114 (5522)	61 (3649)	29 (958)	24 (935)
No. of tDCS (Sample size)	104 (4906)	54 (3092)	27 (919)	23 (915)
No. of tACS (Sample size)	7 (494)	5 (455)	2 (39)	N/A
No. of tRNS (Sample size)	3 (122)	2 (102)	N/A	1(20)
**Sex, Female, No. (%) [a]**	3198 (60.2) [f]	2283 (64.1) [e]	406 (43.3)	508 (62.4) [d]
**Mean Age [a], y**	43.13 [i]	41.54	36.19 [h]	56.18 [g]
**Baseline score [b], mean (SD)**	22.19 (8.64)	23.95 (7.90)	22.35 (9.56)	17.51 (8.06)
**Total charge [c], mean (SD)**	2.02 (4.70)	2.01 (3.12)	3.11 (8.54)	0.94 (0.64)
**Electrode polarity and placements, No. (%)**				
F3				
Anodal F3	85 (74.56)	53 (86.89)	18 (62.07)	14 (58.33)
Cathodal F3	6 (5.26)	0	6 (20.69)	0
Other regions				
Anodal other regions	19 (16.67)	8 (13.11)	2 (6.90)	9 (37.5)
Cathodal other regions	4 (3.50)	0	3 (10.34)	1 (4.17)
**Electrode size, cm^2, No. (%)**				
Rectangular				
19~23 cm^2	6 (5.26)	6 (9.83)	0	0
25 cm^2	36 (31.58)	21 (34.42)	6 (20.69)	9 (37.5)
35 cm^2	52 (45.61)	25 (40.98)	14 (48.28)	13 (54.17)
42 cm^2	3 (2.63)	2 (3.28)	1 (3.45)	0
Elliptical				
0.8 cm^2	1 (0.88)	0	1 (3.45)	0
28.26 cm^2	2 (1.75)	0	1 (3.45)	1 (4.17)
No reported	14 (12.28)	7 (11.48)	6 (20.69)	1 (4.17)
**Current Intensity, No. (%)**				
0.5 mA	2 (1.75)	2 (3.28)	0	0
1 mA	3 (2.63)	2 (3.28)	1 (3.45)	0
1.5 mA	4 (3.51)	2 (3.28)	1 (3.45)	1 (4.67)
2 mA	99 (86.84)	50 (81.97)	26 (89.66)	23 (95.83)
2.5 mA	2 (1.75)	1 (1.64)	1 (3.45)	0
15 mA	2 (1.75)	2 (3.28)	0	0
No reported	2 (1.75)	2 (3.28)	0	0
**Duration of each session, No. (%)**				

© 2025 Ren C et al. *JAMA Network Open*.

10 min	1 (0.88)	1 (1.64)	0	0
15 min	2 (1.75)	2 (3.28)	0	0
20 min	54 (47.37)	20 (32.79)	19 (65.52)	15 (62.5)
25 min	5 (4.39)	4 (6.55)	0	1 (4.67)
30 min	45 (39.47)	29 (16.39)	9 (31.03)	7 (29.17)
40 min	5 (4.39)	4 (6.56)	1 (3.45)	0
60 min	1 (0.88)	1 (1.64)	0	0
No reported	1 (0.88)	1 (1.64)	0	0
**Total number of sessions, No. (%)**				
<10 sessions	19 (16.67)	8 (13.11)	4 (13.8)	7 (29.17)
10 sessions	44 (38.60)	21 (34.43)	12 (41.38)	11 (45.83)
12~15 sessions	29 (25.43)	18 (29.51)	8 (27.59)	2 (8.33)
20~25 sessions	17 (14.91)	11 (18.03)	2 (6.90)	4 (16.67)
>30 sessions	5 (4.39)	3 (4.91)	2 (6.90)	0

[a]Studies not reporting sex or mean age were exclude from the analysis.
[b]Baseline score was calculated by the weighted arithmetic mean of depression scores of active and sham groups.
[c]Total charge = (current intensity (A) × stimulation duration (s) × number of sessions)/electrode's size (cm^2).
[d]Two studies did not report sex.
[e]Three studies did not report sex.
[f]Five studies did not report sex.
[g]One study did not report mean age.
[h]Five studies did not report mean age.
[i]Six studies did not report mean age.

© 2025 Ren C et al. *JAMA Network Open*.

eTable 2. Conditions of the comorbid depression group

Condition	No. of studies
Depression in Psychiatric Comorbidities	29
Bipolar depression	5
Obsessive-compulsive disorder (OCD)	3
Generalized anxiety disorder (GAD)	5
Schizophrenia	4
Addiction	7
Burnout	2
Borderline personality disorder (BPD) Type I	1
Post-traumatic stress disorder (PTSD)	1
Antenatal depression	1
Depression in Medical Comorbidities	24
Post-stroke depression	4
AD	1
Cognitive disorder	1
Fibromyalgia	6
Multiple sclerosis (MS)	1
Pain	5
Epilepsy	3
Premenstrual syndrome	1
Parkinson's disease	1
Older adults with depressive symptoms	1

© 2025 Ren C et al. *JAMA Network Open*.

eTable 3. Summary of tES studies across diagnostic groups

Author, year, country	Diagnosis, sample size (% female), mean age (years)	Interventions (n)	Electrode's location (s) (Anode/ Cathode)	Electrode size (cm2)	Stimulation intensity (mA)	Duration of each treatment or session	Total number of treatments or sessions	Outcome measures	Main results	Adverse events (Yes or no or NR)	Follow-up after the end of intervention (weeks)
**Major Depressive Disorder (MDD) (n=44)**											
tDCS (n=38)											
Brunoni, 2017, United States[1]	MDD,245 (68%),42.8	tDCS + Oral placebo (n=94)<br>Vs<br>sham tDCS + oral placebo (n=60)<br>Vs<br>sham tDCS + escitalopram (n=91)	Anode: the left DLPFC (F3),<br>Cathode: the right DLPFC (F4).	35	2	30	22	Primary outcome:<br>the MADRS<br>Secondary outcomes:<br>HDRS-17, BDI	Improved the MADRS, HDRS-17, and BDI with Escitalopram and tDCS compared to placebo (*p* = 0.01).<br>No significant difference in the MADRS, HDRS-17 and BDI between the active tDCS and Escitalopram groups.	Yes	0
Monnart, 2019, UK[2]	Treatment-resistant MDD, 31 (NR),50	tDCS + mindfulness-based cognitive therapy (MBCT) (n=15)<br>Vs<br>tDCS + non-MBCT (n=16)	Anode: the left DLPFC (F3),<br>Cathode: the right DLPFC (F4)	NR	2	20	10	Primary outcome:<br>the MADRS	Improved the MADRS at T2 with the tDCS + MBCT and tDCS + non-MBCT groups (*p* = 0.008).	NR	2
Vanderhasselt et al., 2015, Brazil[3]	MDD, 33 (73%), 43.6	Active tDCS and PASAT (n=19)<br>Vs<br>sham tDCS and PASAT (n=14)	Anode: the left DLPFC (F3),<br>Cathode: the right DLPFC (F4)	25	2	30	10	Secondary outcomes:<br>HAMD, BDI-II	No significant difference in the HAMD and BDI-II between the active tDCS and sham groups.	NR	0
Bares et al., 2019, Czech Republic[4]	MDD,57 (63%),45.6	Active tDCS +placebo (n=29) Vs<br>sham tDCS + venlafaxine (VNF) (n=28)	Anode: The left DLPFC (F3),<br>Cathode: the right DLPFC (F4)	25	2	30	20	Primary outcome: the MADRS<br>Secondary outcomes: CGI, BDI-SF, OIDS-SR	No significant difference in the MADRS (*p* = 0.17), CGI (*p* = 0.23), BDI-SF (*p* = 0.35), and QIDS-SR (*p* = 0.41) between the active tDCS and sham tDCS +VNF groups.	Yes	0
Pavlova et al., 2018, Sweden[5]	MDD, 68 (30%), 37.7	Active 30-min tDCS + sertraline (n=27)<br>Vs<br>sham tDCS +sertraline (n=20)<br>Vs<br>active 20-min tDCS +sertraline (n=21)	Anode: the left DLPFC (F3),<br>Cathode: the contralateral orbit (F8)	Anode:25<br>Cathode:35	0.5	30 or 20	10	Primary outcomes:<br>HAMD<br>Secondary outcomes: BDI, MADRS	Improved depression (HAMD) in the 30-min ($p < 0.001$) and 20-min ($p = 0.045$) tDCS groups compared to the sham group.	Yes	0

© 2025 Ren C et al. *JAMA Network Open*.

Author, year, country	Diagnosis, sample size (% female), mean age (years)	Interventions (n)	Electrode's location (s) (Anode/ Cathode)	Electrode size (cm2)	Stimulation intensity (mA)	Duration of each treatment or session	Total number of treatments or sessions	Outcome measures	Main results	Adverse events (Yes or no or NR)	Follow-up after the end of intervention (weeks)
Sharafi et al., 2019, Iran[6]	MDD, 30 (53%), 47.2	Active tDCS +SSRI (n=15)<br>Vs<br>sham tDCS + SSRI (n=15)	Anode: the left DLPFC (F3),<br>Cathode: the right DLPFC (F4)	20	2	20	10	Primary outcomes: HDRS-17	Improved HDRS-17 after the intervention ($p < 0.001$) in the active tDCS group compared to the sham group.	NR	4
Loo et al., 2012, Australia[7]	MDD, 64(44%), 48.2	Active tDCS (n=33)<br>Vs<br>sham tDCS (n=31)	Anode: the left DLPFC (F3),<br>Cathode: the lateral aspect of contralateral orbit (F8)	35	2	20	15	Primary outcome: the MADRS<br>Secondary outcomes: IDS, OIDS-C, CGI	Improved the MADRS, QIDS-SR, and QIDS-C with the tDCS group compared to the sham group ($p < 0.05$).	Yes	0
Burkhardt et al., 2023, Germany[8]	MDD, 150(59%), 40.1	Active tDCS + SSRI (n=77)<br>Vs<br>sham tDCS + SSRI (n=73)	Anode: the left DLPFC (F3),<br>Cathode: the right DLPFC (F4)	35	2	30	24	Primary outcome: the MADRS<br>Secondary outcomes: HDRS-21, CGI-S, BDI-II	No intergroup difference was found in improvement on the MADRS at week 6 between the active tDCS group and the sham group.	Yes	24
Palm et al., 2011, Germany[9]	MDD, 22(64%), 57	Active tDCS + antidepressant (n=11)<br>Vs<br>sham tDCS + antidepressant (n=11)	Anode: the left DLPFC (F3),<br>Cathode: the right supraorbital region (Fp2)	35	1 or 2	20	10	Primary outcome: HAMD-24<br>Secondary outcomes: BDI, CGI	Improved the HAMD-24 slightly in active tDCS compared to sham treatment (P = 0.0492).	Yes	0
Moirand et al., 2022, France[10]	MDD, 39(62%), 49.8	Active tDCS + antidepressant (n=21)<br>Vs<br>sham tDCS + antidepressant (n=18)	Anode: the left DLPFC (F3),<br>Cathode: the right DLPFC (F4)	35	2	30	10	Primary outcome: the MADRS	No significant difference in the MADRS between the active and sham tDCS groups.	NR	4

© 2025 Ren C et al. *JAMA Network Open*.

Author, year, country	Diagnosis, sample size (% female), mean age (years)	Interventions (n)	Electrode's location (s) (Anode/ Cathode)	Electrode size (cm2)	Stimulation intensity (mA)	Duration of each treatment or session	Total number of treatments or sessions	Outcome measures	Main results	Adverse events (Yes or no or NR)	Follow-up after the end of intervention (weeks)
Huang et al., 2023, China[11]	MDE, 70(74%), 26.5	rOFC-tDCS (n=23)<br>Vs<br>sham tDCS (24)<br>Vs<br>left DLPFC-tDCS (n=23)	rOFC-tDCS<br>Anode: Fp1<br>Cathod: Fp2<br>Left DLPFC-tDCS<br>Anode: F3<br>Cathode: F4	25	2	25	12	Primary outcome: the MADRS<br>Secondary outcomes: HAMD-17, OIDS-SR	No significant difference in the MADRS between the active two groups and the sham group.	Yes	6
Brunoni et al., 2013, Brazil[12]	MDD,120 (68%), 42.35	Active tDCS and placebo (n=30)<br>Vs<br>Sham tDCS and placebo (n=30)<br>Vs<br>Active tDCS and sertraline (n=30)<br>Vs<br>Sham tDCS and sertraline (n=30)	Anode: the left DLPFC (F3)<br>Cathode: the right DLPFC (F4)	25	2	30	12	Primary outcome: the MADRS<br>Secondary outcomes: HAMD-17, CGI-S, BDI	Improved in the MADRS scores in the combined treatment group (sertraline/active tDCS) compared to sertraline only (p =.002), tDCS only (p=.03), and placebo/sham tDCS (p =.001); in tDCS only compared to placebo/sham tDCS. No significant difference in the MADRS between the tDCS only and sertraline (p =.35).	Yes	6
Boggio et al., 2008, Brazil[13]	MDD, 40(70%), 49.4	Active DLPFC tDCS (n=21)<br>Vs<br>Sham tDCS (n=10)<br>Vs<br>Active occipital tDCS (n=9)	Anode: the left DLPFC (F3) or the occipital cortex,<br>Cathode: the right supraorbital area (Fp2)	35	2	20	10	Primary outcome: HAMD-17	Improved HAMD-17 with DLPFC compared to sham (p=0.0018) and occipital groups (p=0.009) but did not improve between occipital and sham groups (p=0.6).	NR	4
Blumberger et al., 2012, Canada[14]	TRD, 24(83%), 47.5	Active tDCS (n=13)<br>Vs<br>sham tDCS (n=11)	Anode: the left DLPFC (F3)<br>Cathode: the right DLPFC (F4)	35	2	20	15	Primary outcome: HRSD-17<br>Secondary outcomes: MADRS, BDI-II	No difference in HRSD change (p = 0.80), MADRS change (p = 0.55), and BDI-II (p = 0.38) between the two groups.	Yes	0
Ribeiro et al., 2023, Brazil[15]	MDD, 32 (66%), 42.8	Active tDCS (n=16)<br>Vs<br>sham tDCS (n=16)	Anode: the left DLPFC (F3),<br>Cathode: the right supraorbital (Fp2)	25	2	30	10	Primary outcome: HDRS17<br>Secondary outcomes: included	Improved depression (HDRS17) after a 30-day follow-up (p = 0.008) in the active tDCS.	NR	4

© 2025 Ren C et al. *JAMA Network Open*.

Author, year, country	Diagnosis, sample size (% female), mean age (years)	Interventions (n)	Electrode's location (s) (Anode/ Cathode)	Electrode size (cm2)	Stimulation intensity (mA)	Duration of each treatment or session	Total number of treatments or sessions	Outcome measures	Main results	Adverse events (Yes or no or NR)	Follow-up after the end of intervention (weeks)
								satisfaction (TSQM II) and quality of life (WHOQOL-BREF).			
Bennabi et al., 2015, France[16]	TRD, 24(75%), 61.8	Active tDCS +escitalopram (n=12) Vs sham tDCS +escitalopram (n=12)	Anode: the left DLPFC (F3), Cathode: FP2	35	2	30	10	Primary outcome: the MADRS Secondary outcomes: HDRS, BDI, SRRS	No difference in the MADRS, HDRS, and SRRS between the active and sham groups.	Yes	4
Nord et al., 2019, UK[17]	MDD,39(51%), 33.3	Active tDCS + CBT (n=20) Vs sham tDCS + CBT(n=19)	Anode: the left DLPFC (F3), Cathode: ipsilateral deltoid	35	1	20	8	Primary outcome: HAMD	No difference in the HAMD between the active and sham groups.	NR	0
Dastjerdi et al., 2015, Iran[18]	MDD, 30(57%), 31.5	Combined tDCS-CES (n=10) Vs tDCS (n=10) Vs CES (n=10)	NR	NR	NR	20	6	BDI-II	The combined treatment showed more response followed by CES and tDCS treatments (P<0.01).	Yes	0
Loo et al., 2018, Australia[19]	MDE, 120(54%),48.1	Active tDCS (UD=42, BD=19) Vs sham tDCS (UD=42, BD=17)	Anode: the left DLPFC (F3), Cathode: the lateral right frontal area (F8)	35	2.5	30	20	Primary outcome: the MADRS Secondary outcomes: CGI-S, QIDS-SR	No antidepressant difference between active and sham tDCS groups for unipolar or bipolar depression. Improved mood (MADRS, CGI, and QIDS-SR) over the 4-week treatment period in both unipolar ($p < 0.001$) and bipolar groups ($p < 0.001$).	Yes	0
Bennabi et al., 2020, France[20]	TRD-MDD, 23(65%), 60.2	Active tDCS + escitalopram (n=12) Vs sham tDCS +escitalopram (n=11)	Anode: the left DLPFC (F3), Cathode: the contralateral supraorbital area (FP2)	35	2	30	5	Secondary outcomes: HDRS-21, SRRS 27	No significant differences were found within the depression measures (HDRS-21, SRRS 27) between the active and sham tDCS groups.	NR	0
Aust et al., 2022, Germany[21]	MDD, 148(61%), 41.1	Active tDCS+ CBT (n=48) Vs	Anode: the left DLPFC (F3),	NR	2	30	12	Primary outcome: the MADRS	No significant differences in the MADRS and BDI-II were	Yes	18, 30

© 2025 Ren C et al. *JAMA Network Open*.

Author, year, country	Diagnosis, sample size (% female), mean age (years)	Interventions (n)	Electrode's location (s) (Anode/ Cathode)	Electrode size (cm2)	Stimulation intensity (mA)	Duration of each treatment or session	Total number of treatments or sessions	Outcome measures	Main results	Adverse events (Yes or no or NR)	Follow-up after the end of intervention (weeks)
		sham tDCS + CBT (n=47)<br>Vs<br>CBT alone (n=53)	Cathode: the right DLPFC (F4)					Secondary outcomes: BDI-II, SHAPS-D	found between these three groups.		
Park et al., 2020, Korea[22]	MDD, 92(63%), 44.6	Active tDCS+ placebo (n=45)<br>Vs<br>sham tDCS + Sertraline (n=47)	Anode: the left DLPFC (F3),<br>Cathode: the right DLPFC (F4)	25	2	30	10	Primary outcome: the MADRS<br>Secondary outcomes: BDI-II, CGI-S	No significant differences in the MADRS and BDI-II were found between the two groups.	Yes	0
Zhou et al., 2020, China[23]	MDD, 90(67%), 42.2	Active tDCS (n=47)<br>Vs<br>sham tDCS (n=43)	Anode: the left DLPFC (F3),<br>Cathode: the right DLPFC (F4)	25	2	30	20	Primary outcome: PSQI<br>Secondary outcomes: SAS, SDS	Improved total scores of SAS and SDS in the active tDCS group compared to the sham tDCS group.	NR	0
Oh et al., 2022, Korea[24]	MDD, 45(44%), 29.1	Active tDCS +escitalopram (n=20)<br>Vs<br>sham tDCS + escitalopram (n=25)	Anode: the left DLPFC (F3),<br>Cathode: the right DLPFC (F4)	NR	2	30	30	Primary outcome: HAMD<br>Secondary outcomes: the MADRS, BDI, PDQ-D	Improved depression (BDI-II) in the active tDCS group compared to the sham group. No significant differences in the MADRS and HAMD were found between the two groups.	Yes	0
Loo et al. 2010, Australia[25]	MDD, 40(55%), 47.3	Active tDCS (n=20)<br>Vs<br>sham tDCS (n=20)	Anode: the left DLPFC (F3),<br>Cathode: the lateral aspect of the contralateral orbit (Fp2)	35	1	20	10	Primary outcome: the MADRS<br>Secondary outcomes: HAMD-17, CGI-S, BDI	Improved the HAMD17 (p < 0.001, MADRS (p < 0.001) after 10 sessions in active tDCS compared to sham group.<br>No difference between active Vs sham after 5 sessions	Yes	0

© 2025 Ren C et al. *JAMA Network Open*.

Author, year, country	Diagnosis, sample size (% female), mean age (years)	Interventions (n)	Electrode's location (s) (Anode/ Cathode)	Electrode size (cm2)	Stimulation intensity (mA)	Duration of each treatment or session	Total number of treatments or sessions	Outcome measures	Main results	Adverse events (Yes or no or NR)	Follow-up after the end of intervention (weeks)
Segrave et al., 2014, Australia[26]	MDD, 27(37%),40.44	Active tDCS+ Cognitive control training (CTT) (n=9)<br>Vs<br>tDCS + sham CTT (n=9)<br>Vs<br>sham tDCS +CTT (n=9)	Anode: the left DLPFC (F3),<br>Cathode: lateral aspect of the contralateral orbit (Fp2)	35	2	24	5	Primary outcome: the MADRS<br>Secondary outcomes: BDI-II	Improved depression (MADRS) at three-week follow-up in the tDCS + CCT group compared to the tDCS + sham CCT and sham + CCT group.	NR	3
Mayur et al., 2018, Australia[27]	MDD, 16(38%), 44.94	Active tDCS + ECT (n=8)<br>Vs<br>sham tDCS + ECT (n=8)	Anode: the left DLPFC (F3),<br>Cathode: the right DLPFC (F4)	25	2	30	10	Primary outcome: the MADRS	No significant differences in the MADRS were found between the two groups.	NR	0
Chen et al., 2023, China[28]	MDD. 63(73%), 28.6	Active tDCS 60min/d (n=22)<br>Vs<br>sham tDCS (n=16)<br>Vs<br>active tDCS 30 min/d (n=25)	Anode: the left DLPFC (F3),<br>Cathode: the right OFC.	NR	2	60 or 30	12	Primary outcome: HAMD-17	No significant differences in the HAMD-17 were found between these three groups.	Yes	0
Brunoni et al., 2014, Brazil[29]	MDD, 37(30%), 43.8	Active tDCS + CCT (n=20)<br>Vs<br>sham tDCS +CCT (n=17)	Anode: left DLPFC (F3)<br>Cathode: right DLPFC (F4)	25	2	30	10	Primary outcome: HDRS<br>Secondary outcomes:<br>BDI	Improved HDRS and BDI after the acute treatment (week 2) and at endpoint (week 4) in CCT alone and combined with tDCS compared to sham group.	None	2
Kumari et al., 2023, India[30]	MDD, 50(50%), 30.7	Active tDCS (n=26)<br>Vs<br>sham tDCS (n=24) both along with escitalopram 10 mg/day	Anode: left DLPFC (F3)<br>Cathode: right DLPFC (F4)	25	2	20	10	Outcome: HAMD-17, BDI	Improve depression (HAMD, BDI) at week 2 in the active tDCS group compared to the sham group.	Yes	2

© 2025 Ren C et al. *JAMA Network Open*.

Author, year, country	Diagnosis, sample size (% female), mean age (years)	Interventions (n)	Electrode's location (s) (Anode/ Cathode)	Electrode size (cm2)	Stimulation intensity (mA)	Duration of each treatment or session	Total number of treatments or sessions	Outcome measures	Main results	Adverse events (Yes or no or NR)	Follow-up after the end of intervention (weeks)
Borrione et al., 2024, Brasil[31]	MDE, 210 (86%), 38.9	Home-use tDCS plus a digital psychological intervention (double active) (n=64)<br>Vs<br>sham home-use tDCS plus digital placebo (double sham) (n = 73)<br>Vs<br>home-use tDCS plus digital placebo (tDCS only) (n = 73)	Anode: the left DLPFC (F3)<br>Cathode: the right DLPFC (F4)	22.9	2	30	21	Primary outcomes: HDRS-17	No statistically significant group differences in treatment by time interactions for HDRS-17 scores, and the estimated effect sizes between groups were as follows: double active Vs tDCS only (Cohen d, 0.05; 95% CI, −0.48 to 0.58; P = .86), double active Vs double sham (Cohen d, −0.20; 95% CI, −0.73 to 0.34; P = .47), and tDCS only Vs double sham (Cohen d, −0.25; 95% CI, −0.76 to 0.27; P = .35).	Yes	0
Ha et al., 2024, United States[33]	Late-life MDD and cognitive decline, 24 (NR), 75.4	Active tDCS +CT (n = 12)<br>Vs<br>sham tDCS + CT (n = 12)	Anode: the left DLPFC (F3)<br>Cathode: the right DLPFC (F4)	35	2	30	10	Primary outcomes: Ham-D	No significant differences in the Ham-D were found between the two groups.	NR	0
Kim et al., 2024, Republic of Korea[34]	MDD and MCI, 37 (65%), 74.6	Active Home-based tDCS (n=14)<br>Vs<br>sham tDCS (n=23)	Anode: the left DLPFC (F3)<br>Cathode: the right DLPFC (F4)		2	30	31	Secondary outcomes: CESD, MADRS, Ham-D	No significant differences in the CESD, MADRS, and Ham-D were found between the two groups.	NR	0

© 2025 Ren C et al. *JAMA Network Open*.

Author, year, country	Diagnosis, sample size (% female), mean age (years)	Interventions (n)	Electrode's location (s) (Anode/ Cathode)	Electrode size (cm2)	Stimulation intensity (mA)	Duration of each treatment or session	Total number of treatments or sessions	Outcome measures	Main results	Adverse events (Yes or no or NR)	Follow-up after the end of intervention (weeks)
Kong et al., 2024, China[35]	MDD, 70 (74%), 27.3	rOFC-tDCS (n=23)<br>Vs<br>DLPFC-tDCS (n=23)<br>Vs<br>Sham tDCS (n=24)	Anode: the left DLPFC (F3) or the right OFC (Fp2)<br>Cathode: the right DLPFC (F4)	Round 19.63	2	20	12	Primary outcomes: HAMD-17, QIDS-SR16, SHAPS	The rate of decrease in HAMD-17 scores after consolidation treatment differed significantly between the three groups (p = 0.003). Post-hoc analysis showed a significant difference in the rate of decrease in HAMD-17 scores between the OFC and DLPFC groups (p = 0.027).	Yes	8
Li et al., 2024, China[36]	MDD, 240 (61%), 31.21	Active rTMS + active tDCS (n =60)<br>Vs<br>active rTMS + sham tDCS (n =60)<br>Vs<br>sham rTMS + active tDCS (n =60)<br>Vs<br>sham rTMS + sham tDCS (n =60)	Anode: the left DLPFC (F3)<br>Cathode: the right DLPFC (F4)	25	2	20	10	Secondary outcomes: HDRS-24	Improved the HDRS-24 total scores in the combined treatment group compared to the active tDCS group (p=0.001) and sham group (p<0.001) or between the active rTMS group and tDCS (p=0.013) or active rTMS group and sham group (p<0.001).	Yes	0
Nejati et al., 2024, Iran[37]	MDD, 40 (100%), 36.82	CBT (n = 10)<br>Vs<br>tDCS (n = 10)<br>Vs<br>CBT + tDCS (n = 10)<br>Vs<br>control (n = 10)	Anode: the left DLPFC (F3)<br>Cathode: the right DLPFC (F4)	25	2	20	10	Primary outcomes: BDI-II	Improved the BDI-II scores in all three intervention groups Vs. the control group in the postintervention and follow-up measurements.	Yes	4
Tabasi et al., 2024, Iran[38]	MDD with overweight, 64 (NR), 39.25	Active tDCS + omaga3 (n = 16)<br>Vs<br>sham tDCS + placebo (n = 17)<br>Vs<br>sham tDCS + omega 3 (n = 15)<br>Vs<br>active tDCS + placebo (n = 16)	Anode: the left DLPFC (F3)<br>Cathode: the right DLPFC (F4)	35	1.5	15	12	Primary outcomes: BDI-II	No significant difference in depression score between the experimental groups and the control group.	Yes	4

© 2025 Ren C et al. *JAMA Network Open*.

Author, year, country	Diagnosis, sample size (% female), mean age (years)	Interventions (n)	Electrode's location (s) (Anode/ Cathode)	Electrode size (cm2)	Stimulation intensity (mA)	Duration of each treatment or session	Total number of treatments or sessions	Outcome measures	Main results	Adverse events (Yes or no or NR)	Follow-up after the end of intervention (weeks)
Woodham et al., 2023, UK[39]	MDD, 174 (69%), 37.7	Active Home-based tDCS (n=87)<br>Vs<br>sham tDCS (n=87)	Anode: the left DLPFC (F3)<br>Cathode: the right DLPFC (F4)	23	2	30	36	Primary Outcomes: HDRS-17.<br>Secondary outcomes: MADRS, C-SSRS	Improved HDRS from baseline to week 10 in active tDCS treatment arm compared to sham tDCS treatment group (p = 0.012).	Yes	0
**tACS (n=4)**											
Wang et al., 2022, China[40]	MDD, 100(74%),40.0	Active tACS (n=50)<br>Vs<br>sham tACS (n=50)	Anode: forehead corresponding to Fpz, Fp1 and Fp2<br>Cathode: the mastoid region of each side	Anode:42<br>Cathode: 12	15	40	20	**Primary outcome:** HDRS-17<br>**Secondary outcomes:** CGI-S, CGI-I	Improve depression (HDRS-17) at weeks 4 and 8 in the active tACS group compared to the sham group (p < 0.01).	Yes	8
Alexander et al., 2019, USA[41]	MDD, 32(84%), 36.69	Active 10 Hz-tACS (n=10)<br>Vs<br>Active 40 Hz-tACS (n=11)<br>Vs<br>sham tACS (n=11)	Anode: the left DLPFC (F3) and the right DLPFC (F4)<br>Cathode: the vertex (Cz)	Anode:35<br>Cathode: 25	2	40	5	**Primary outcome:** the MADRS<br>**Secondary outcomes:** The change in raw alpha power-EEG, HDRS-17, BDI	-No significant interaction between the treatment condition (10 Hz-tACS, 40 Hz-tACS, sham) and session (baseline to 4 weeks after completion of treatment)<br>-The 10 Hz-tACS group had more responders (MADRS and HDRS) than 40 Hz-tACS and sham groups (p = 0.026) from baseline to 2 weeks after intervention.	Yes	4

© 2025 Ren C et al. *JAMA Network Open*.

Author, year, country	Diagnosis, sample size (% female), mean age (years)	Interventions (n)	Electrode's location (s) (Anode/ Cathode)	Electrode size (cm2)	Stimulation intensity (mA)	Duration of each treatment or session	Total number of treatments or sessions	Outcome measures	Main results	Adverse events (Yes or no or NR)	Follow-up after the end of intervention (weeks)
Zhou et al., 2024, China[42]	MDD, 66 (78.8%), 28.4	Active tACS (n=33) Vs sham tACS (n=33) Combined with escitalopram	Anode: the forehead (Fpz, Fp1, and Fp2) Cathode: each side of the mastoid	Anode: 42 Cathode: 12	15	40	20	Primary outcomes: HAMD-17	Improved the HAMD-17 scores at week 4 in active group compared to sham group (p =0.001).	Yes	0
Gehrman et al., 2024, United States[32]	MDD, 255 (73%), 39.8	Active tACS (n = 126) Vs sham tACS (n = 129)	Anode: the left squamous temporal bone Cathode: the right squamous temporal bone		2	20	40	Primary outcomes: BDI-II, Secondary outcomes: PHQ-9, QIDS-SR	Improved the BDI-II in the active treatment versus control at 2 weeks (p = .005).	Yes	0
**tRNS (n=2)**											
Schecklmann et al., 2021, Germany[43]	MDD, 40 (40%) 46	Active tRNS (n=19) Vs sham tRNS (n=17)	Anode: the left DLPFC (F3) Cathode: the right DLPFC (F4)	35	2	20	15	**Primary outcome:** HAMD **Secondary outcomes:** MDI, CGI-I	No significant differences in the HAMD between the active tRNS and sham groups.	None	9
Nikolin et al., 2020, Australia[44]	MDD, 66 (45%), 48.8	Active tRNS (n=32) Vs sham tRNS (n=34)	Anode: the left DLPFC (F3) Cathode: F8	35	2	30	20	**Primary outcome:** the MADRS **Secondary outcomes:** CGI-I, BDI-II	No significant differences in the MADRS, BDI-II, and CGI-I between the active tRNS and sham groups.	Yes	0
**Depression in Psychiatric Comorbidities (n=23)**											
**tDCS (n=21)**											

© 2025 Ren C et al. *JAMA Network Open*.

Author, year, country	Diagnosis, sample size (% female), mean age (years)	Interventions (n)	Electrode's location (s) (Anode/ Cathode)	Electrode size (cm2)	Stimulation intensity (mA)	Duration of each treatment or session	Total number of treatments or sessions	Outcome measures	Main results	Adverse events (Yes or no or NR)	Follow-up after the end of intervention (weeks)
**Obsessive-compulsive disorder (OCD) (n=1)**											
Shafiezadeh et al., 2021, Iran[45]	OCD, 24(29%), NR	Active tDCS right DLPFC (n=10)<br>Vs<br>sham tDCS (n=6)<br>Vs<br>active tDCS left DLPFC (n=8)	Anode: F4 or F3<br>Cathode: Fp1 or Fp2	35	2	30	5	Outcomes: DASS-21	No significant differences were observed in the DASS-21 scores between groups.	NR	0
**Bipolar Disorder (BD) (n=4)**											
Sampaio-Junior et al., 2018, Brazil[46]	BD,59(68%), 45.9	Active tDCS + pharmacologic regimen (n=30)<br>Vs<br>sham tDCS + pharmacologic regimen (n=29)	Anode: the left DLPFC (F3)<br>Cathode: the right DLPFC (F4)	25	2	30	12	Primary outcomes: the MADRS, Secondary outcomes: HAM-D, CGI	Improved the MADRS, HAM-D scores in the active tDCS group compared to sham group (p = .01).	Yes	0
Lee et al., 2022, South Korea[47]	BD, 64(73%), 33.4	Active tDCS (n=32)<br>Vs<br>sham tDCS (n=32)	Anode: the left DLPFC (F3)<br>Cathode: the right DLPFC (F4)	28	2	30	42	Primary outcomes: HDRS-17	No significant differences in the HDRS-17 were found between the two groups.	Yes	0
Mardani et al., 2021, Iran[48]	BD, 30(57%), 30.33	Active tDCS + medication (n=15)<br>Vs medication (n=15)	Anode: the left DLPFC (F3),<br>Cathode: the right DLPFC (F4)	NR	2	20	20	Primary outcomes: HDRS-21	Improved HDRS-21 (p <0.001) in both tDCS and medication compared to the group received mediation.	NR	0
Zhang et al, 2023, China[49]	BD, 50(68%), 33.06	Active HD-tDCS (n=25)<br>Vs<br>sham HD-tDCS (n=25)	Anode: the left DLPFC (F3)<br>Cathode: FP1, FZ, C3, F7	1	2	20	14	Primary outcomes: HAMD<br>Secondary outcomes: BDI	Improved HAMD and BDI (p <0.01) in the active tDCS group compared to the sham group.	Yes	0
Generalized anxiety disorder (GAD) (n=3)											
Lima et al., 2019, Brazil[50]	GAD, 30(37%), 30.5	Active tDCS (n=15)<br>Vs<br>sham tDCS (n=15)	Anode: the left DLPFC (F3),<br>Cathode: the contralateral supraorbital (Fp2)	35	2	20	5	Secondary outcomes: BDI-II	No significant differences in BDI-II were found between the two groups.	Yes	1
Movahed et al., 2018, Iran[51]	GAD, 18(46%), 28.7	Active tDCS (n=6)<br>Vs<br>sham tDCS (n=6)	Cathode: the right prefrontal cortex (F4)<br>Anode: the left deltoid	NR	2	20	10	Secondary outcomes: HAM-D	Improved HAM-D (p<0.05) with tDCS compared to sham.	NR	8

© 2025 Ren C et al. *JAMA Network Open*.

Author, year, country	Diagnosis, sample size (% female), mean age (years)	Interventions (n)	Electrode's location (s) (Anode/ Cathode)	Electrode size (cm2)	Stimulation intensity (mA)	Duration of each treatment or session	Total number of treatments or sessions	Outcome measures	Main results	Adverse events (Yes or no or NR)	Follow-up after the end of intervention (weeks)
		Vs pharmacotherapy (n=6)									
Nasiri et al., 2020, Iran[52]	GAD, 43(70%), 20.76	Active tDCS + Unified protocol (UP) (n=15) Vs wait-list control (n=15) Vs UP alone (n=13)	Cathode: the right DLPFC Anode: the contralateral deltoid	25	2	30	10	Primary outcomes: BDI-II	Improved BDI-II with active tDCS +UP group compared to the wait-list group ($p < 0.001$).	NR	12
Schizophrenia (n=3)											
Palm et al., 2016, Germany[53]	Paranoid schizophrenia, 20(25%), 36.10	Active tDCS (n=12) Vs sham tDCS (n=14)	Anode: the left DLPFC (F3), Cathode: the right orbitofrontal region (Fp2)	35	2	20	10	Secondary outcomes: CDSS	Improved CDSS with active tDCS at follow-up compared to sham tDCS ($p = 0.042$).	Yes .	0
Jeon et al., 2018, Republic of Korea[54]	Schizophrenia, 54(52%), 39.93	Active tDCS (n=26) Vs sham tDCS (n=28)	Anode: the left DLPFC (F3), Cathode: the right DLPFC (F4)	25	2	30	10	Secondary outcomes: CDSS	Improved CDSS with active tDCS compared to sham tDCS ($p = 0.047$).	None	0
Lisoni, et al, 2024, Italy[55]	Schizophrenia, 50 (22%), 42.70	Active tDCS (n=25) Vs sham tDCS (n=25)	Anode: the left DLPFC (F3), Cathode: the right orbitofrontal region (Fp2)	35	2	20	15	Outcomes: CDSS	Improved CDSS with active tDCS compared to sham ($p < 0.05$).	NR	0
Addiction (n=5)											
Taremian et al., 2019, Iran[56]	Opium use disorder, 60(0%), 33.26	Active tDCS + methadone maintenance treatment (MMT) (n=20) Vs sham tDCS + MMT (n=20) Vs MMT (n=20)	Anode: the right DLPFC (F4), Cathode: the left DLPFC (F3)	35	2	20	10	Secondary outcomes: BDI-II	Improved BDI-II with active tDCS compared to control group ($p= 0.009$).	NR	0
Naeim et al., 2021, Iran[57]	Methadone users, 60(0%), 35.00	Active tDCS (n=30) Vs control group (n=30)	Anode: the right DLPFC (F4), Cathode: the left DLPFC (F3)	35	2	20	10	Secondary outcomes: BDI-II	Improved BDI-II with active tDCS compared to control group ($p < 0.01$).	NR	0
Batista et al., 2015, Brazil[58]	Crack-Cocaine Dependence, 36(0%), 30.40	Active tDCS (n=17) Vs sham tDCS (n=19)	Anode: the left DLPFC (F3), Cathode: the right DLPFC (F4)	35	2	20	5	Secondary outcomes: HAM-D	No significant differences in the HAM-D were found between the two groups.	Yes	0

© 2025 Ren C et al. *JAMA Network Open*.

Author, year, country	Diagnosis, sample size (% female), mean age (years)	Interventions (n)	Electrode's location (s) (Anode/ Cathode)	Electrode size (cm2)	Stimulation intensity (mA)	Duration of each treatment or session	Total number of treatments or sessions	Outcome measures	Main results	Adverse events (Yes or no or NR)	Follow-up after the end of intervention (weeks)
Eskandari et al., 2019, Iran[59]	Opioid-addiction, 30(0%), 27.20	Anodal tDCS (n=10) Vs sham tDCS (n=10) Vs cathodal tDCS (n=10)	Anode/ Cathode: the left DLPFC (F3), Cathode/ Anode: the right DLPFC (F4)	NR	0.5-2	20	10	Secondary outcomes: DASS-21	Improved DASS-21 with the active tDCS compared to sham tDCS (p = 0.018).	NR	0
Sadeghi Bimorgh et al, 2020, Iran[60]	Opioid-dependent, 27(0%), 36.68	Active tDCS (n=14) Vs sham group (n=13)	Anode: the left DLPFC (F3), Cathode: the right DLPFC (F4)	35	2	20	7	Secondary outcomes: DASS-21	Improved DASS-21 with the active tDCS compared to sham tDCS (p < 0.001).	None	0
Burnout (n=2)											
Van Noppen et al., 2020, Belgium[61]	Burnout, 15(67%), 44.9	Active tDCS (n=8) Vs sham tDCS (n=7)	Anode: the left DLPFC (F3), Cathode: the right orbitofrontal region (Fp2)	25	2	20	15	Secondary outcomes: BDI-II	No significant differences in the BDI-II were found between the two groups.	None	0
Wilczek-Ruzyczka et al., 2021, Poland[62]	Burnout, 40(100%), 50.45	Active tDCS (n=20) Vs sham tDCS (n=20)	Anode: Left DLPFC (F3) Cathode: Contralateral mastoid.	NR	2	30	8	Secondary outcomes: BDI-II	Improved the MADRS and BDI-II with active tDCS compared to sham tDCS (p = 0.001).	NR	0
Post-traumatic stress disorder (PTSD) (n=1)											
Ahmadizadeh et al., 2019, Iran[63]	PTSD, 40(65%), 43.75	Active tDCS (n=20) Vs sham tDCS (n=20)	Anode: the left DLPFC (F3), Cathode: the right DLPFC (F4)	35	2	20	10	Secondary outcomes: BDI-II	Improved BDI-II with active tDCS compared to sham (p < 0.05).	Yes	4
Antenatal depression (n=1)											
Vigod et al., 2019, Canada[64]	Antenatal depression, 20(100%),32.3	Active tDCS (n=10) Vs sham tDCS (n=10)	Anode: the left DLPFC (F3), Cathode: the right DLPFC (F4)	35	2	30	15	Primary outcomes: the MADRS	No significant differences in the MADRS between the active tDCS and sham groups	Yes	0
Borderline personality disorder (BPD) (n=1)											
Lisoni et al., 2020, Italy[65]	BPD, 30(60%), 40.3	Active tDCS (n=15) Vs sham tDCS (n=15)	Anode: the right DLPFC (F4), Cathode: the left DLPFC (F3)	35	2	20	15	Secondary outcomes: HAMD-17, BDI-II	Improve HAM-D (p < 0.001) and BDI-II (p = 0.03) with the active tDCS compared to the sham group.	NR	0
**tACS (n = 2)**											

© 2025 Ren C et al. *JAMA Network Open*.

Author, year, country	Diagnosis, sample size (% female), mean age (years)	Interventions (n)	Electrode's location (s) (Anode/ Cathode)	Electrode size (cm2)	Stimulation intensity (mA)	Duration of each treatment or session	Total number of treatments or sessions	Outcome measures	Main results	Adverse events (Yes or no or NR)	Follow-up after the end of intervention (weeks)
Perera et al., 2023, Australia[66]	OCD, 25(52%),36.12	Active home-based tACS (n=13)<br>Vs<br>sham tACS (n=12)	Anode: bilateral medial prefrontal cortex (mPFC) (AFz),<br>Cathode: Iz	42	1.5	30	39	outcomes: QIDS-SR	Improve depression (QIDS-SR) at 3-month follow-up in the active tACS group compared to the sham group (p =0.036).	None	12
Zhang et al., 2022, United States[67]	Schizophrenia, 25(36%), NR	Active tACS + tACS (n=8)<br>Vs<br>tACS + sham (n=6)<br>Vs<br>sham + tACS (n=5)<br>Vs sham + sham (n=6)<br>Cross over	Anode: the left DLPFC (F3),<br>Cathode: between T3 and P3 in the left TPJ	25 or 35	1	40	13	Secondary outcomes: PANSS depression subscale	Improved depressive symptoms with active tACS compared to sham tACS (p < 0.05).	None	0
**Depression in Medical Comorbidities (n=21)**											
tDCS (n=20)											
Cognitive disorder (n=2)											
Khedr et al., 2019, Egypt[68]	Alzheimer Disease, 44(41%), 64.73	Active tDCS (n=23)<br>Vs<br>sham tDCS (n=21)	Anode: T3-P3 or T4-P4<br>Cathode: the deltoid muscle of the left arm	35	2	20	10	Secondary outcomes: the Cornell Scale for depression	Improved depressive symptoms with active tDCS compared to sham tDCS (p = 0.001).	None	0
Brooks et al., 2021, Canada[69]	Depressed or anxious older adults with subjective cognitive decline, 26(69%), 68.7	Active tDCS + mindfulness-based stress reduction (MBSR) (n=12)<br>Vs<br>sham tDCS + MBSR (n=14)	Anode: bilateral the DLPFC (Fz)<br>Cathode: Iz	25	2	30	10	Secondary outcomes: PROMIS	No significant differences in PROMIS depression scores with active tDCS compared to sham tDCS.	Yes	0
Pain (n=5)											
Mariano et al., 2019, United States[70]	Chronic low back pain, 21(14.2%), 63.1	Active tDCS (n=10) Vs sham tDCS (n=11)	Cathode: FC1<br>Anode: contralateral mastoid.	35	2	20	10	Secondary outcomes: PHQ-9	Improved PHQ-9 at six-week follow-up in the active tDCS group compared to the sham group (p = 0.003).	NR	6
Quintiliano et al., 2022, Brazil[71]	Chronic pain in ESRD, 30(27%), 54.1	Active tDCS (n=15)<br>Vs<br>sham tDCS (n=15)	Anode: Left primary Motor cortex M1 (C3)<br>Cathode: Right supraorbital region (Fp2)	35	2	20	10	Secondary outcomes: BDI-II	Improved BDI-II with active tDCS group compared to the sham group (p = 0.01).	Yes	0
Ibrahim et al., 2018, Egypt[72]	Visceral pain, 40(33%),57.88	Active tDCS (n=20)<br>Vs<br>sham tDCS (n=20)	Anode: M1<br>Cathode: Opposite supraorbital region	35	2	30	10	Secondary outcomes: HAM-D	Improved BDI-II at one month after active tDCS group	Yes	4

© 2025 Ren C et al. *JAMA Network Open*.

Author, year, country	Diagnosis, sample size (% female), mean age (years)	Interventions (n)	Electrode's location (s) (Anode/ Cathode)	Electrode size (cm2)	Stimulation intensity (mA)	Duration of each treatment or session	Total number of treatments or sessions	Outcome measures	Main results	Adverse events (Yes or no or NR)	Follow-up after the end of intervention (weeks)
									compared to the sham group (p = 0.002).		
Gueserse et al., 2022, Iran[73]	Chronic pain, 30(NR), NR	Active tDCS (n=15)<br>Vs<br>ACT (n=15)	Anode: Left DLPFC (F3)<br>Cathode: Right LPFC (F4)	25	2	20	12	Primary outcomes<br>: BDI-II	No significant improvement in depression between the two groups.	NR	4
Kim et al., 2022, Republic of Korea[74]	Chronic musculoskeletal pain, 25(64%), 76.9	Active tDCS +PT(n=13)<br>Vs<br>sham tDCS + PT (n=12)	Anode: Left DLPFC<br>Cathode: Right LPFC	28	2	30	24	Primary outcomes<br>: BDI-II	Improved BDI-II with active tDCS group compared to the sham group (p = 0.014).	NR	4
Epilepsy (n=3)											
Mota et al., 2021, Brazil[75]	Temporal lobe epilepsy, 33(85%), 43.31	Active home-based tDCS (n=23)<br>Vs<br>sham tDCS (n=23)	Anode: the left DLPFC (F3),<br>Cathode: the right DLPFC (F4)	35	2	20	20	Primary outcomes: BDI-II	No significant differences in the BDI-II were found between the two groups.	Yes	48
Liu et al., 2016, United States[76]	Temporal lobe epilepsy, 26(54%), 54.57	Active tDCS (n=21)<br>Vs<br>sham tDCS (n=12)	Anode: the left DLPFC (F3),<br>Cathode: the right supraorbital region (Fp2)	35	2	20	5	Primary outcomes: BDI-II	Improved BDI-II immediately after active tDCS group compared to the sham group (p=0.05).	Yes	2
Azmoodeh et al., 2021, Iran[77]	Epilepsy, 30(47%), 36.43	Active tDCS (n=15)<br>Vs<br>sham tDCS (n=15)	Anode: the left DLPFC (F3),<br>Cathode: the right DLPFC (F4)	35	2	20	20	Primary outcomes: DASS-21	Improved DASS-21 with active tDCS group compared to the sham group (p < 0.001).	NR	0
Fibromyalgia (n=3)											
Khedr et al., 2017, Egypt[78]	Fibromyalgia (FM),<br>36 (94%)<br>32.6	Active tDCS (n=18)<br>Vs<br>sham tDCS (n=18)	Anode: the M1 (C3)<br>Cathode: the contralateral arm (extracephalic)	24	2	20	10	Secondary outcomes: HAM-D	Significant improvement in depression (HAM-D, p<0.05) with tDCS compared to sham.	Yes	4
Arroyo-Fernandez et al., 2022, Spain[79]	Fibromyalgia (FM),<br>120 (94%),<br>50.29	Active tDCS + exercising (n=40)<br>Vs<br>sham tDCS + exercising (n=40)<br>Vs<br>no-intervention control (n=40)	Anode: Primary motor cortex (M1) of the left hemisphere<br>Cathode: Right supraorbital area (Fp2)	25	2	20	5	Secondary outcomes: BDI-II	Significant improvement in depression (BDI-II, p<0.05) with tDCS compared to sham.	None	4
Paula et al., 2023, Brazil[80]	Fibromyalgia (FM),	Active tDCS + Low-dose Naltrexone (LDN) (n=21)	Anode: Primary motor cortex (M1) contralateral to the dominant cortex	35	2	20	5	Secondary outcomes: BDI-II	Improved depression (BDI-II, p<0.001) with tDCS + LDN compared to sham.	Yes	3

© 2025 Ren C et al. *JAMA Network Open*.

Author, year, country	Diagnosis, sample size (% female), mean age (years)	Interventions (n)	Electrode's location (s) (Anode/ Cathode)	Electrode size (cm2)	Stimulation intensity (mA)	Duration of each treatment or session	Total number of treatments or sessions	Outcome measures	Main results	Adverse events (Yes or no or NR)	Follow-up after the end of intervention (weeks)
	86 (100%), 49.33	Vs sham tDCS + LDN (n=22) Vs active tDCS + placebo (n=22) Vs sham tDCS + placebo (n=22)	Cathode: Contralateral supraorbital area								
Older adults with depressive symptoms (n=1)											
Szymkowicz et al., 2022, United States[81]	Older adults, 15(67%), 70.93	Active tDCS (n=7) Vs sham tDCS (n=8)	Cathode: the left DLPFC (F3), Anode: the right DLPFC (F4)	35	2	20	10	Primary outcomes: BDI-II	Improved BDI-II (p = 0.055) with tDCS compared to sham.	NR	0
Multiple Sclerosis (MS) (n=1)											
Chalah et al., 2020, France[82]	MS, 11(73%), 43.91	Active tDCS (n=11) Vs sham tDCS (n=11) Crossover	Cathode: the left DLPFC (F3), Anode: the right DLPFC (F4)	25	2	20	5	Secondary outcomes: HADS	No significant differences in the HADS between the active tDCS and sham groups	None	1
Parkinson's disease (n=1)											
Manenti et al., 2018, Italy[83]	PD, 22(45%), 64.65	Active tDCS +Computerized cognitive training (CCT) (n=11) Vs sham tDCS + CCT (n=11)	Anode: left DLPFC (F3) Cathode: right supraorbital area (Fp2)	35	2	25	10	Secondary outcomes: BDI-II	Improved BDI-II (p < 0.05) with tDCS compared to sham.	None	12
Post-stroke depression (n=3)											
Zanardi et al., 2020, Italy[84]	Vascular Depression, 93(NR), 71.64	Active tDCS daily (n=31) Vs sham tDCS (n=31) Vs active tDCS twice per day (n=31)	Anode: the left DLPFC (F3), Cathode: the contralateral supraorbital area	25	2	30	10	Primary outcomes: HAM-D	Improved HAM-D with active tDCS twice per day treatment compared to sham (p < 0.001).	Yes	0
Valiengo et al., 2017, Brazil[85]	Post-stroke depression, 48(50%), 61.75	Active tDCS (n=24) Vs sham tDCS (n=24)	Anode: the left DLPFC (F3), Cathode: the right DLPFC (F4)	25	2	30	12	Primary outcomes: the MADRS, HDRS-17	Improved the MADRS and HDRS-17 with active tDCS compared to sham (p < 0.001).	Yes	0

© 2025 Ren C et al. *JAMA Network Open*.

Author, year, country	Diagnosis, sample size (% female), mean age (years)	Interventions (n)	Electrode's location (s) (Anode/ Cathode)	Electrode size (cm2)	Stimulation intensity (mA)	Duration of each treatment or session	Total number of treatments or sessions	Outcome measures	Main results	Adverse events (Yes or no or NR)	Follow-up after the end of intervention (weeks)
An et al., 2017, Republic of Korea[86]	Post-stroke depression, 40(25%), 56.7	Active tDCS + conventional occupational therapy (n=20)<br>Vs<br>sham tDCS + conventional occupational therapy (n=20)	Anode: the left DLPFC (F3),<br>Cathode: the right DLPFC (F4)	NR	2	30	20	Primary outcomes: BDI, SS-QOL	Improved depression (p < 0.05) and QOL (p < 0.05) in the experimental group.	NR	0
Primary Sjogren Syndrome (PSS) (n=1)											
Pinto et al., 2021, Brazil[87]	Primary Sjogren Syndrome, 36(0%), 54.45	Active tDCS (n=18)<br>Vs<br>sham tDCS (n=18)	Anode: the left DLPFC (F3),<br>Cathode: the right DLPFC (F4)	35	2	20	5	Secondary outcomes: BDI-II	Improved BDI-II with active tDCS compared to sham (p < 0.05).	Yes	2
**tRNS (n = 1)**											
Curatolo et al., 2017, Italy[88]	Fibromyalgia (FM),<br>20 (100%)<br>42.8	Active tRNS (n=10)<br>Vs<br>sham tRNS (n=10)	Anode: the M1 (C3)<br>Cathode: the right supra-orbital region (Fp2)	35	1.5	10	10	Primary outcomes: HAM-D	Significant improvement in pain (VAS, p<0.005), depression (HADS-Depression, p<0.05), anxiety (HADS-Anxiety, p<0.001), and disability (FIQ, p=0.014) with tRNS compared to sham.	NR	0

Abbreviation: BDI, Beck Depression Inventor; CGI, Clinical Global Impression; CGI-S, Clinical Global Impression scale – Severity of Illness; CDSS, Calgary Depression Scale for Schizophrenia; DLPFC, dorsolateral prefrontal cortex; DASS-21, Depression Anxiety Stress Scales-21; FIQ, Fibromyalgia Impact Questionnaire; GCS, Geriatric Depression Scale; HAM-D, Hamilton Depression Rating Score; HDRS, Hamilton Depression Rating Scale; IDS, Inventory Depressive Symptomatology; IDSSR, the Inventory of Depressive Symptomatology; MDD, Major depressive disorder; MDE, Major depressive episode; MADRS, the Montgomery–Åsberg Depression Rating Scale; MDI, Major Depression Inventory; NR: Not Reported; PHQ-9, the Patient Health Questionnaire; PDQ-D, Perceived Deficits Questionnaire-Depression; PFC, prefrontal cortex; PROMIS, the Patient Reported Outcomes Measurement Information System; QIDS-SR, the participant-rated Quick Inventory of Depressive Symptomatology; QIDS-C, the clinician-rated Quick Inventory of Depressive Symptomatology; RCT, randomized clinical trial; RRS-1, the 10-item Ruminative Response Scale; SRRS, the Salpêtrière Retardation Rating Scale; SHAPS, Snaith–Hamilton Pleasure Scale-Depression; SDS, Self-rating Depression Scale; SAS, Self-rating Anxiety Scale; SS-QOL, the stroke-specific quality of life. TRD, treatment-resistant depression; tDCS, transcranial direct current stimulation, tRNS, transcranial random noise stimulation; tES, transcranial electric stimulation; tACS, transcranial alternating current stimulation; VAS, Visual analog scale (VAS); ZUNG, Self-Rating Depression Scale.

© 2025 Ren C et al. *JAMA Network Open*.

eFigure. Risk of bias assessment for randomized control trials

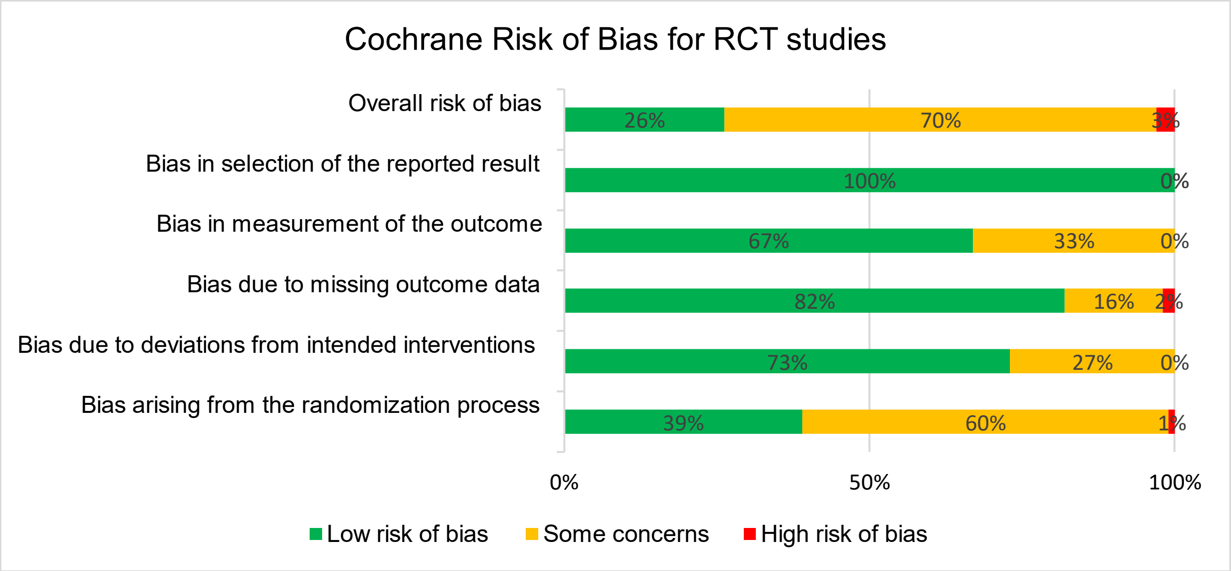

© 2025 Ren C et al. *JAMA Network Open*.

eTable 4. Pooled Effect Size and Quality of Evidence for Primary Depression symptoms

Comparison	Diagnostic group	SMD (95%CI)	No. of studies (Sample Size)	I^2	Factors that affect the quality of evidence	Overall quality of evidence	Publication bias (Egger's test)
**tES Vs sham/no treatment**	All patients	-0.59 (-0.83 to -0.35)	73 (3537 patients)[1,7,11-15,19,23,25,28,31,34-39,45,47,49-51,53-55,57-65,68,70-72,74-82,84-87,32,40-42,66,67,43,44,88]	84%	Inconsistency, potential publication bias	Low	*P* = 0.002
	MDD	-0.22 (-0.44 to 0.01)	31 (2024 patients) [1,7,11-15,19,23,25,28,31,34-39,43,44]	72%	Inconsistency, Imprecision (wide CIs)	Low	*P* = 0.946
	DPC	-0.78 (-1.27 to -0.29)	22 (699 patients) [19,45,47,49-51,53-55,57-65,66,67]	84%	Inconsistency, Risk of Bias (methodological issues)	Low	*P* = 0.238
	DMC	-1.05 (-1.67 to -0.43)	20 (814 patients) [68,70-72,74-82,84,87,85,88]	89%	Inconsistency, potential publication bias	Low	*P* = 0.006
tDCS Vs sham/no treatment	All patients	-0.65 (-0.92 to -0.37)	63 (2921 patients) [1,7,11-15,19,23,25,28,31,34-39,45,47,49-51,53-55,57-65,68,70-72,74-82,84-87,32,40-42]	85%	Inconsistency, potential publication bias	Low	*P* = 0.003
	MDD	-0.18 (-0.46 to 0.10)	24 (1467 patients)[1,7,11-15,19,23,25,28,31,34-39]	72%	Inconsistency	Moderate	*P* = 0.608
	DPC	-0.88 (-1.40 to -0.36)	20 (660 patients)[19,45,47,49-51,53-55,57-65]	84%	Inconsistency, Risk of Bias (methodological issues)	Low	*P* = 0.138
	DMC	-1.05 (-1.70 to -0.40)	19 (794 patients)[68,70-72,74-82,84,87,85]	90%	Inconsistency, potential publication bias, Risk of Bias (methodological issues)	Very low	*P* = 0.007
tACS Vs sham	All patients	-0.42 (-0.85 to 0.01)	7 (494 patients)[32,40-42,66,67]	58%	Imprecision (wide CIs)	Moderate	N/A
	MDD	-0.58 (-0.96 to -0.20)	5 (455 patients)[32,40-42]	49%	None	High	N/A

© 2025 Ren C et al. *JAMA Network Open*.

	DPC	0.32 (-1.38 to 2.03)	2 (39 patients)[66,67]	0%	Severe imprecision (small sample size and wide CIs)	Low	N/A
tRNS Vs sham	All patients	-0.08 (-1.73 to 1.57)	3 (122 patients)[43,44,88]	65%	Imprecision (small sample size)	Moderate	N/A
	MDD	0.23 (0.03 to 0.43)	2 (102 patients)[43,44]	0%	Imprecision (small sample size)	Moderate	N/A
	DMC	-1.02 (-1.96 to -0.07)	1 (20 patients)[88]	N/A	Severe imprecision (small sample size and wide CIs)	Low	N/A
**tDCS Vs antidepressant, pharmacotherapy, or psychotherapy**							
tDCS Vs antidepressant	MDD	0.37 (-0.00 to 0.74)	3 (332 patients)[1,4,22]	0%	Imprecision (wide CIs)	Moderate	N/A
tDCS Vs pharmacotherapy	DPC	-1.80 (-3.23 to -0.38)	1 (12 patients)[51]	N/A	Severe imprecision (small sample size and wide CIs)	Low	N/A
tDCS Vs psychotherapy	All patients	0.24 (-4.30 to 4.78)	2 (48 patients) [26,73]	30%	Severe imprecision (small sample size and wide CIs)	Low	N/A
	MDD	0.68 (-0.28 to 1.64)	1 (18 patients)[26]	N/A	Severe imprecision (small sample size and wide CIs)	Low	N/A
	DMC	-0.05 (-0.77 to 0.67)	1 (30 patients)[73]	N/A	Severe imprecision (small sample size and wide CIs)	Low	N/A
**tDCS Combined with Medications**							
tDCS + antidepressant Vs sham + antidepressant	MDD	-0.51 (-0.90 to -0.13)	12 (591 patients)[5,6,8-10,12,16,20,24,30]	76%	Inconsistency	Moderate	P = 0.056
tDCS + pharmacotherapy Vs sham + pharmacotherapy	All patients	-0.87 (-1.32 to -0.41)	5 (212 patients) [46,48,56,80]	19%	Imprecision (small sample size)	Moderate	N/A
	DPC	-0.73 (-0.93 to -0.53)	4 (169 patients)[46,48,56]	0%	imprecision (small sample size)	Moderate	N/A
	DMC	-1.54 (-2.23 to -0.85)	1 (43 patients)[80]	N/A	Imprecision (small sample size)	Moderate	N/A
**tDCS Combined with Psychotherapy**							

© 2025 Ren C et al. *JAMA Network Open*.

tDCS + psychotherapy Vs sham + psychotherapy	All patients	-0.15 (-0.33 to 0.02)	9 (292 patients) [3,17,21,26,29,33,69,83]	0%	Severe imprecision (small sample size and wide CIs)	Low	N/A
	MDD	-0.11 (-0.30 to 0.07)	7 (244 patients)[3,17,21,26,29,33]	0%	Severe imprecision (small sample size and wide CIs)	Low	N/A
	DMC	-0.35 (-3.36 to 2.67)	2 (48 patients)[69,83]	0%	Severe imprecision (small sample size and wide CIs)	Low	N/A
tDCS + psychotherapy Vs sham	MDD	-0.04 (-0.37 to 0.30)	1 (137 patients)[31]	N/A	Severe imprecision (small sample size and wide CIs)	Low	N/A
tDCS + psychotherapy Vs psychotherapy	All patients	-0.38 (-1.07 to 0.32)	3 (132 patients) [21,37,52]	N/A	Severe imprecision (small sample size and wide CIs)	Low	N/A
	MDD	-0.48 (-4.29 to 3.32)	2 (104 patients)[21,37]	36%	Severe imprecision (small sample size and wide CIs)	Low	N/A
	DPC	-0.27 (-1.02 to 0.48)	1 (28 patients)[52]	N/A	Severe imprecision (small sample size and wide CIs)	Low	N/A
tDCS + psychotherapy Vs tDCS	MDD	-0.40 (-1.11 to 0.31)	1 (31 patients)[2]	N/A	Severe imprecision (small sample size and wide CIs)	Low	N/A
tDCS + psychotherapy Vs wait-listing	DPC	-2.55 (-3.54 to -1.55)	1 (30 patients)[52]	N/A	Imprecision (small sample size and wide CIs)	Low	N/A
**tDCS Combined with OBS**							
tDCS + OBS Vs sham + OBS	MDD	-0.33 (-0.91 to 0.24)	3 (156 patients)[18,27,36]	0%	Imprecision (small sample size and wide CIs)	Low	N/A
tDCS Vs sham + OBS	MDD	-0.12 (-0.99 to 0.76)	1 (20 patients)[18]	N/A	Imprecision (small sample size and wide CIs), Risk of Bias (methodological issues)	Very low	N/A

CI = Confidence Interval; DMC indicates depression with medical comorbidities; DPC, depression with psychiatric comorbidities; I^2 = Heterogeneity index; MDD, major depressive disorder; OBS: other brain stimulation techniques (e.g., electroconvulsive therapy [ECT], cranial electrotherapy stimulation [CET], repetitive transcranial magnetic stimulation [rTMS]); N/A, Not Applicable; Pharmacotherapy indicates treatment involving additional medications (e.g.,

© 2025 Ren C et al. *JAMA Network Open*.

methadone, naltrexone); SMD, standard mean difference; tES, transcranial electrical stimulation; tDCS, transcranial direct current stimulation; tACS, transcranial alternating current stimulation; tRNS, transcranial random noise stimulation.

© 2025 Ren C et al. *JAMA Network Open*.

eTable 5. Pooled Effect Size and Quality of Evidence for Treatment Response and Remission

Intervention	Outcome, Diagnostic group	No. of Studies (Sample Size)	OR (95%CI)	*I²*	Factors that affect the quality of evidence	Overall quality of evidence	Publication bias (Egger's test)
**tES Vs sham**	Response, all patients	25 (1729 patients)[1,7,11-15,19,25,28,31,36,39,46,85,32,40-42,44,19,46,85]	1.38 (0.95 to 2.04)	55%	Imprecision (wide CIs)	Moderate	P = 0.09
	Remission, all patients	22 (1285 patients)[1,7,11-15,19,25,31,39,19,46,55,64,85]	1.54 (0.95 to 2.50)	53%	Imprecision (wide CIs)	Moderate	P = 0.45
	Response, MDD	22 (1589 patients)[1,7,11-15,19,25,28,31,36,39,32,40-42,44]	1.26 (0.85 to 1.87)	53%	Imprecision (wide CIs)	Moderate	P = 0.03
	Remission, MDD	17 (1078 patients)[1,7,11-15,19,25,31,39]	1.20 (0.69 to 2.07)	54%	Imprecision (wide CIs)	Moderate	P = 0.06
tDCS Vs sham	Response, all patients	19 (1216 patients)[1,7,11-15,19,25,28,31,36,39,46,85]	1.33 (0.83 to 2.10)	57%	Imprecision (wide CIs)	Moderate	P = 0.29
	Remission, all patients	18 (1084 patients)[1,7,11-15,19,25,31,39,19,46,55,64,85]	1.51 (0.88 to 2.60)	54%	Imprecision (wide CIs)	Moderate	P = 1.00
	Response, MDD	16 (1076 patients)[1,7,11-]	1.15 (0.72 to 1.84)	55%	Imprecision (wide CIs)	Moderate	P = 0.12

© 2025 Ren C et al. *JAMA Network Open*.

Intervention	Outcome, Diagnostic group	No. of Studies (Sample Size)	OR (95%CI)	I^2	Factors that affect the quality of evidence	Overall quality of evidence	Publication bias (Egger's test)
		[15,19,25,28,31,36,39]					
	Remission, MDD	13 (877 patients)[1,7,11-15,19,25,31,39]	1.10 (0.61 to 2.01)	55%	Imprecision (wide CIs)	Moderate	P = 0.20
	Response, DPC	2 (92 patients)[19,46]	2.20 (0.41 to 11.76)	62%	Severe imprecision (small sample size and wide CIs)	Low	N/A
	Remission, DPC	4 (159 patients)[19,46,55,64]	3.30 (1.07 to 10.23)	45%	Severe imprecision (small sample size and wide CIs)	Low	N/A
	Response, DMC	1 (48 patients)[85]	13.80 (1.58 to 120.38)	N/A	Severe imprecision (small sample size and wide CIs)	Low	N/A
	Remission, DMC	1 (48 patients) [85]	13.82 (0.72 to 265.52)	N/A	Severe imprecision (small sample size and wide CIs)	Low	N/A
tACS Vs sham	Response, MDD	5 (447 patients)[32,40-42]	2.07 (1.34 to 3.19)	28%	None	High	N/A
	Remission, MDD	3 (135 patients)[40,41]	2.11 (0.64 to 6.98)	45%	Severe imprecision (small sample size and wide CIs)	Low	N/A
tRNS Vs sham	Response, MDD	1 (66 patients)[44]	0.19 (0.02 to 1.70)	N/A	Severe imprecision (small sample size and wide CIs)	Low	N/A
	Remission, MDD	1 (66 patients)[44]	0.20 (0.01 to 4.33)	N/A	Severe imprecision (small sample size and wide CIs)	Low	N/A
**tDCS Vs antidepressant, psychotherapy**							
tDCS Vs antidepressant	Response, MDD	3 (334 patients)[1,4,22]	0.63 (0.40 to 0.98)	0%	None	High	N/A

© 2025 Ren C et al. *JAMA Network Open*.

Intervention	Outcome, Diagnostic group	No. of Studies (Sample Size)	OR (95%CI)	I^2	Factors that affect the quality of evidence	Overall quality of evidence	Publication bias (Egger's test)
	Remission, MDD	3 (334 patients)[1,4,22]	0.63 (0.39 to 1.01)	0%	Imprecision (wide CIs)	Moderate	N/A
tDCS Vs psychotherapy	Response, MDD	1 (18 patients)[26]	0.06 (0.00 to 1.43)	N/A	Severe imprecision (small sample size and wide CIs)	Low	N/A
	Remission, MDD	1 (18 patients)[26]	0.16 (0.01 to 3.81)	N/A	Severe imprecision (small sample size and wide CIs)	Low	N/A
**tDCS Combined with Medications**							
tDCS + antidepressant Vs sham + antidepressant	Response, MDD	9 (473 patients)[5,6,8,10,12,16,24,30]	2.25 (1.08 to 4.65)	23%	Imprecision (wide CIs)	Moderate	N/A
	Remission, MDD	10 (496 patients)[5,6,8,10,12,16,20,24,30]	1.29 (0.71 to 2.35)	22%	Imprecision (wide CIs)	Moderate	N/A
tDCS + psychotherapy Vs sham + psychotherapy	Response, MDD	4 (179 patients)[17,21,26,29]	1.26 (0.67 to 2.36)	0%	Severe imprecision (small sample size and wide CIs)	Low	N/A
	Remission, MDD	4 (179 patients)[17,21,26,29]	1.50 (0.72 to 3.13)	0%	Severe imprecision (small sample size and wide CIs)	Low	N/A
tDCS + psychotherapy Vs psychotherapy	Response, MDD	1 (84 patients)[21]	0.76 (0.31 to 1.83)	N/A	Severe imprecision (small sample size and wide CIs)	Low	N/A
	Remission, MDD	1 (84 patients) [21]	1.03 (0.42 to 2.54)	N/A	Severe imprecision (small sample size and wide CIs)	Low	N/A
tDCS + psychotherapy Vs sham	Response, MDD	1 (137 patients)[31]	0.76 (0.36 to 1.48)	N/A	Severe imprecision (small sample size and wide CIs)	Low	N/A
	Remission, MDD	1 (137 patients) [31]	0.63 (0.26 to 1.56)	N/A	Severe imprecision (small sample size and wide CIs)	Low	N/A
**tDCS Combined with OBS**							

© 2025 Ren C et al. *JAMA Network Open*.

Intervention	Outcome, Diagnostic group	No. of Studies (Sample Size)	OR (95%CI)	I^2	Factors that affect the quality of evidence	Overall quality of evidence	Publication bias (Egger's test)
tDCS + OBS Vs sham + OBS	Response, MDD	2 (136 patients)[27,36]	1.95 (0.86 to 4.41)	N/A	Severe imprecision (small sample size and wide CIs)	Low	N/A

CI = Confidence Interval; DMC, depression with medical comorbidities; DPC, depression with psychiatric comorbidities; I² = Heterogeneity index; MDD, major depressive disorder; N/A, Not Applicable; OBS: other brain stimulation techniques (e.g., electroconvulsive therapy [ECT], cranial electrotherapy stimulation [CET], repetitive transcranial magnetic stimulation [rTMS]); OR, Odds Ratio; tES, transcranial electrical stimulation; tDCS, transcranial direct current stimulation; tACS, transcranial alternating current stimulation; tRNS, transcranial random noise stimulation.

© 2025 Ren C et al. *JAMA Network Open*.

eTable 6. Pooled effect size and quality of evidence for adverse events (including dropouts, dropouts due to any adverse events, and adverse events)

Comparison	Outcome	Diagnostic group	Conclusion	No. of studies and sample size	Factors that affect the quality of evidence	Overall quality of evidence
**tES Vs sham/no treatment**	Dropouts	All patients	OR:1.01; 95% CI: 0.76 to 1.33; I^2=0%	73(3524 patients) [1,7,11-15,19,23,25,28,31,34-39,45,47,49-51,53-55,57-65,68,70-72,74-82,84-87,32,40-42,66,67,43,44,88]	Imprecision (wide CIs)	Moderate
	Dropouts due to AEs	All patients	OR:1.84; 95% CI: 0.65 to 5.22; I^2=0%	32 (2001 patients) [1,7,11-15,19,23,25,28,31,34-39,32,40-42,65]	Imprecision (wide CIs)	Moderate
	Any AEs (Severe)	All patients	OR:1.91; 95% CI: 1.25 to 2.93; I^2=0%	8 (692 patients) [1,7,11,12,19,39,47,49]	None	High
	Any AEs (Mild to moderate)	All patients	**OR:1.44; 95% CI: 1.26 to 1.65; I^2=42%**	32 (1988 patients)[1,7,11,12,14,19,25,32,35,36,38-42,44,47,49,50,58,64,72,75,78,84,85,87]	None	High
tDCS Vs. Sham/no treatment	Dropouts	All patients	OR: 1.08; 95% CI: 0.81 to 1.43; I^2=0%	63 (2921 Patients) [1,7,11-15,19,23,25,28,31,34-39,45,47,49-51,53-55,57-65,68,70-72,74-82,84-87,32,40-42]	Imprecision (wide CIs)	Moderate
	Dropouts due to AEs	All patients	OR: 1.86; 95% CI: 0.74 to 4.71; I^2=0%	26 (1471 Patients) [1,7,11-15,19,23,25,28,31,34-39]	Imprecision (wide CIs)	Moderate
	Any AEs (Severe)	All patients	OR: 1.91; 95% CI: 1.24 to 2.93; I^2=0%	8 (692 Patients)[1,7,11,12,19,39,47,49]	None	High
	Any AEs (Mild to moderate)	All patients	OR: 1.37; 95% CI: 1.19 to 1.58; I^2=43%	27 (1478 Patients) [1,7,11,12,14,19,25,35,36,38,39,47,49,50,58,64,72,75,78,84,85,87]	None	High
tACS Vs sham	Dropouts	All patients	OR: 1.01; 95% CI: 0.22 to 4.58; I^2= 57%	7 (494 patients) [32,40-42,66,67]	Imprecision (wide CIs)	Moderate
	Dropouts due to AEs	All patients	OR: 3.09; 95% CI: 0.12 to 78.7; I^2= N/A	5 (596 patients) [32,40-42]	Severe imprecision (small sample size and wide CI)	Low
	Any AEs (Mild to moderate)	All patients	OR: 2.01; 95% CI:	5 (464 patients)[32,40-42]	None	High

© 2025 Ren C et al. *JAMA Network Open*.

Comparison	Outcome	Diagnostic group	Conclusion	No. of studies and sample size	Factors that affect the quality of evidence	Overall quality of evidence
			1.49 to 2.72; I^2= 0%			
tRNS Vs sham	Dropouts	All patients	OR: 0.32; 95% CI: 0.08 to 1.28; I^2= N/A	3 (122 patients) [43,44,88]	Severe imprecision (small sample size and wide CI)	Low
	Any AEs (Mild to moderate)	All patients	OR: 2.71; 95% CI: 1.70 to 4.31; I^2= N/A	1 (66 patients)[44]	Imprecision (small sample size)	Moderate
**tES Vs antidepressant, pharmacotherapy, or psychotherapy**						
tDCS Vs antidepressant	Dropouts	All patients	OR: 1.40; 95% CI: 0.80 to 2.43; I^2= 57%	3 (434 patients) [1,4,22]	Severe imprecision (small sample size and wide CI)	Low
	Any AEs (Severe)	All patients	OR: 1.00; 95% CI: 0.06 to 16.67; I^2= 0%	2 (204 patients)[1,4]	Imprecision (wide CIs)	Moderate
	Any AEs (Mild to moderate)	All patients	OR: 1.16; 95% CI: 0.78 to 1.70; I^2=0%	3 (300 Patients)[1,4,22]	Imprecision (wide CIs)	Moderate
tDCS Vs pharmacotherapy	Dropouts	All patients	No dropout	1 (12 patients) [51]	N/A	N/A
tDCS Vs psychotherapy	Dropouts	All patients	No dropout	2 (48 patients) [26,73]	N/A	N/A
**tDCS Combined with Medication**						
tDCS + antidepressant Vs sham + antidepressant	Dropouts	All patients	OR: 1.04; 95% CI: 0.48 to 2.26; I^2= 24%	12 (591 patients) [5,6,8-10,12,16,20,24,30]	Imprecision (wide CIs)	Moderate
	Dropouts due to AEs	All patients	OR: 0.99; 95% CI: 0.00 to 11254619.10; I^2= 59%	2 (220 patients)[8,12]	Severe imprecision (small sample size and wide CI)	Low
	Any AEs (Mild to moderate)	All patients	OR: 1.17; 95% CI: 0.78 to 1.74; I^2= 37%	6 (403 Patients)[5,8,12,24,30]	Imprecision (wide CIs)	Moderate
	Any AEs (Severe)	All patients	OR: 1.81; 95% CI: 0.04 to 73.62; I^2= 0%	3 (261 patients)[5,8,12]	Imprecision (wide CIs)	Moderate

© 2025 Ren C et al. *JAMA Network Open*.

Comparison	Outcome	Diagnostic group	Conclusion	No. of studies and sample size	Factors that affect the quality of evidence	Overall quality of evidence
tDCS + pharmacotherapy Vs sham + pharmacotherapy	Dropouts	All patients	OR: 1.55; 95% CI: 0.40 to 5.90; I^2= 0%	5 (112 patients) [46,48,56,80]	Imprecision (wide CIs)	Moderate
	Any AEs (Mild to moderate)	All patients	OR: 1.09; 95% CI: 0.79 to 1.50; I^2= N/A	1 (53 Patients)[46]	Severe imprecision (small sample size and wide CI)	Low
**tDCS Combined with Psychotherapy**						
tDCS + psychotherapy Vs sham + psychotherapy	Dropouts	All patients	OR: 0.63; 95% CI: 0.29 to 1.38; I^2= 0%	9 (299 patients) [3,17,21,26,29,33,69,83]	None	High
	Any AEs (Mild to moderate)	All patients	OR: 2.58; 95% CI: 0.28 to 23.46; I^2= 74%	2 (121 Patients)[21,69]	Severe imprecision (small sample size and wide CI)	Low
tDCS + psychotherapy Vs sham	Dropouts	All patients	OR: 0.85; 95% CI: 0.18 to 3.94; I^2= N/A	1 (137 patients) [31]	Severe imprecision (small sample size and wide CI)	Low
tDCS + psychotherapy Vs psychotherapy	Dropouts	All patients	OR: 0.85; 95% CI: 0.06 to 11.20; I^2= 62%	3 (137 patients) [21,37,52]	Severe imprecision (small sample size and wide CI)	Low
	Any AEs (Mild to moderate)	All patients	OR: 0.93; 95% CI: 0.69 to 1.24; I^2= 0%	2 (121 Patients)[21,37]	Severe imprecision (small sample size and wide CI)	Low
tDCS + psychotherapy Vs tDCS	Dropouts	All patients	No dropout	1 (31 patients) [2]	N/A	N/A
tDCS + psychotherapy Vs wait-listing	Dropouts	All patients	OR: 5.74; 95% CI: 0.25 to 130.37; I^2= N/A	1 (30 patients) [52]	Severe imprecision (small sample size and wide CI)	Low
**tDCS Combined with OBS**						
tDCS + OBS Vs sham + OBS	Dropouts	All patients	OR: 1.55; 95% CI: 0.61 to 3.92; I^2= 0%	3 (156 patients) [18,27,36]	Severe imprecision (small sample size and wide CI)	Low
	Any AEs (Mild to moderate)	All patients	OR: 1.17; 95% CI: 0.39 to 3.47; I^2= N/A	1 (120 Patients)[36]	Severe imprecision (small sample size and wide CI)	Low

© 2025 Ren C et al. *JAMA Network Open*.

Comparison	Outcome	Diagnostic group	Conclusion	No. of studies and sample size	Factors that affect the quality of evidence	Overall quality of evidence
tDCS Vs sham + OBS	Dropouts	All patients	OR: 5.74; 95% CI: 0.25 to 130.37; I^2= N/A	1 (30 patients) [18]	Severe imprecision (small sample size and wide CI)	Low

AEs, Adverse Events; CI = Confidence Interval; DMC, depression with medical comorbidities; DPC, depression with psychiatric comorbidities; I^2 = Heterogeneity index; MDD, major depressive disorder; N/A, Not Applicable; OBS: other brain stimulation techniques (e.g., electroconvulsive therapy [ECT], cranial electrotherapy stimulation [CET], repetitive transcranial magnetic stimulation [rTMS]); OR, Odds Ratio; Pharmacotherapy indicates treatment involving additional medications (e.g., methadone, naltrexone); tES, transcranial electrical stimulation; tDCS, transcranial direct current stimulation; tACS, transcranial alternating current stimulation; tRNS, transcranial random noise stimulation.

© 2025 Ren C et al. *JAMA Network Open*.

eTable 7. Sensitivity Analysis for Primary Depression Symptoms: Exclusion of Outlier Studies (Hedges' g ≥ 1.5)

**Comparison**	**Diagnostic group**	**Conclusion**	**No. of studies (Sample Size)**
**tES Vs sham/no treatment**	All patients	SMD: -0.28; 95% CI: -0.42 to -0.15; I^2 = 61%	63 (3136 patients) [1,7,11-13,23,31,34-36,38,39,19,45,47,50,53-55,58-61,63-65,43,44,68,70-72,75,76,78-82,85,87,32,40-42,66,67,43,44,88]
	MDD	SMD: -0.18; 95% CI: -0.37 to -0.07; I^2 = 68%	30 (2004 patients) [1,7,11-13,23,31,34-36,38,39,32,40-42,43,44]
	DPC	SMD: -0.40; 95% CI: -0.68 to -0.11; I^2 = 52%	18 (537 patients) [19,45,47,50,53-55,58-61,63-65,66,67]
	DMC	SMD: -0.40; 95% CI: -0.67 to -0.13; I^2 = 50%	15 (595 patients) [68,70-72,75,76,78-82,85,87,88]
tDCS Vs sham/no treatment	All patients	SMD: -0.28; 95% CI: -0.43 to -0.13; I^2 = 60%	53 (2520 patients) [1,7,11-13,23,31,34-36,38,39,19,45,47,50,53-55,58-61,63-65,43,44,68,70-72,75,76,78-82,85,87]
	MDD	SMD: -0.13; 95% CI: -0.35 to 0.09; I^2 = 66%	23 (1447 patients)[1,7,11-13,23,31,34-36,38,39]
	DPC	SMD: -0.47; 95% CI: -0.77 to -0.18; I^2 = 51%	16 (498 patients)[19,45,47,50,53-55,58-61,63-65]
	DMC	SMD: -0.37; 95% CI: -0.64 to -0.09; I^2 = 50%	14 (575 patients)[68,70-72,75,76,78-82,85,87]
tACS Vs sham	All patients	SMD: -0.42; 95% CI: -0.85 to 0.01; I^2 = 58%	7 (494 patients) [32,40-42,66,67]
	MDD	SMD: -0.58; 95% CI: -0.96 to -0.20; I^2 = 49%	5 (455 patients)[32,40-42]
	DPC	SMD: 0.32; 95% CI: -1.38 to 2.03; I^2 = 0%	2 (39 patients)[66,67]
tRNS Vs sham	All patients	SMD: -0.08; 95% CI: -1.73 to 1.57; I^2 = 65%	3 (122 patients) [43,44,88]
	MDD	SMD: 0.23; 95% CI: 0.03 to 0.43; I^2 = 0%	2 (102 patients)[43,44]
	DMC	SMD: -1.02; 95%CI: -1.96 to -0.07; I^2 = N/A	1 (20 patients)[88]
**tES Vs medication or psychotherapy**			
tDCS Vs antidepressant	MDD	SMD: 0.37; 95% CI: -0.00 to 0.74; I^2 = 0%	3 (332 patients) [1,4,22]
tDCS Vs psychotherapy	All patients	SMD: 0.24; 95% CI: -4.30 to 4.78; I^2 = 0%	2 (48 patients) [26,73]
	MDD	SMD: 0.68; 95% CI: -0.28 to 1.64; I^2 = N/A	1 (18 patients) [26]
	DMC	SMD: -0.05; 95% CI: -0.77 to 0.67; I^2 = N/A	1 (30 patients) [73]
**tDCS Combined with Medications**			
tDCS + antidepressant Vs sham + antidepressant	MDD	SMD: -0.42; 95% CI: -0.76 to -0.07; I^2 = 71%	11 (561 patients)[5,8-10,12,16,20,24,30]
tDCS + pharmacotherapy Vs sham + pharmacotherapy	All patients	SMD: -0.73; 95% CI: -0.92 to -0.53; I^2 = 0%	4 (169 patients) [46,48,56]
**tDCS Combined with Psychotherapy**			
tDCS + psychotherapy Vs sham + psychotherapy	All patients	SMD: -0.15; 95% CI: -0.33 to 0.02; I^2 = 0%	8 (284 patients) [3,17,21,26,29,33,69,83]
	MDD	SMD: -0.11; 95% CI: -0.30 to 0.07; I^2 = 0%	6 (236 patients)[3,17,21,26,29,33]
	DMC	SMD: -0.35; 95% CI: -3.36 to 2.67; I^2 = 0%	2 (48 patients)[69,83]
tDCS + psychotherapy Vs sham	MDD	SMD: 0.04; 95% CI: -0.37 to 0.30; I^2 = N/A	1 (137 patients)[31]

© 2025 Ren C et al. *JAMA Network Open*.

tDCS + psychotherapy Vs psychotherapy	All patients	SMD: -0.38; 95% CI: -1.07 to 0.32; I^2 = 0%	3 (132 patients) [21,37,52]
	MDD	SMD: -0.48; 95% CI: -4.29 to 3.32; I^2 = 0%	2 (104 patients)[21,37]
	DPC	SMD: -0.27; 95% CI: -1.02 to 0.48; I^2 = N/A	1 (28 patients)[52]
tDCS + psychotherapy Vs tDCS	MDD	SMD: -0.40; 95% CI: -1.11 to 0.31; I^2 = N/A	1 (31 patients)[2]
**tDCS Combined with OBS**			
tDCS + OBS Vs sham + OBS	MDD	SMD: -0.33; 95% CI: -0.91 to 0.24; I^2 = 0%	3 (156 patients)[18,27,36]
tDCS Vs sham + OBS	MDD	SMD: -0.12; 95% CI: -0.99 to 0.76; I^2 = N/A	1 (20 patients)[18]

CI = Confidence Interval; DPC = Depression in Psychiatric Comorbidities; DMC = Depression in Medical Comorbidities. F3 refers to the left dorsolateral prefrontal cortex (DLPFC) based on the 10/20 EEG electrode placement system; I^2 = Heterogeneity index; MDD = Major Depressive Disorder; "Other regions" include Fpz (frontopolar), T3 (left temporal), C3 (left central), FC1 (frontal-central), Fp2 (right frontopolar), OFC (orbitofrontal cortex), and occipital cortex. OBS: other brain stimulation techniques (e.g., electroconvulsive therapy [ECT], cranial electrotherapy stimulation [CET], repetitive transcranial magnetic stimulation [rTMS]); SMD = Standardized Mean Difference; tDCS, transcranial direct current stimulation, tRNS, transcranial random noise stimulation; tES, transcranial electric stimulation; tACS, transcranial alternating current stimulation.

© 2025 Ren C et al. *JAMA Network Open*.

eTable 8. Sensitivity Analysis for Response and Remission: Exclusion of Outlier Studies (OR > 5)

**Intervention**	**Outcome, Diagnostic group**	**No. of Studies (Sample Size)**	**OR (95%CI)**	I^2
**tES Vs sham**	Response, all patients	23 (1652 patients) [1,7,11-15,19,25,28,31,36,39,46,85,32,40-42,44,19,46]	1.26 (0.86 to 1.84)	52%
	Remission, all patients	18 (1139 patients) [1,7,11-15,19,25,31,39,19,46,55,64]	1.21 (0.74 to 1.99)	51%
	Response, MDD	21 (1560 patients) [1,7,11-15,19,25,28,31,36,39,32,40-42,44]	1.20 (0.80 to 1.77)	52%
	Remission, MDD	16 (1047 patients) [1,7,11-15,19,25,31,39]	1.14 (0.65 to 2.00)	56%
tDCS Vs sham	Response, all patients	17 (1139 patients) [1,7,11-15,19,25,28,31,36,39,46,85]	1.15 (0.74 to 1.81)	53%
	Remission, all patients	14 (938 patients) [1,7,11-15,19,25,31,39,19,46,55,64]	1.13 (0.67 to 1.90)	52%
	Response, MDD	15 (1047 patients) [1,7,11-15,19,25,28,31,36,39]	1.06 (0.67 to 1.68)	52%
	Remission, MDD	12 (846 patients) [1,7,11-15,19,25,31,39]	1.03 (0.56 to 1.91)	57%
	Response, DPC	2 (92 patients) [19,46]	2.20 (0.41 to 11.76)	62%
	Remission, DPC	4 (159 patients) [19,46,55,64]	3.30 (1.07 to 10.23)	45%
tACS Vs sham	Response, MDD	5 (447 patients) [32,40-42]	2.07 (1.34 to 3.19)	28%
	Remission, MDD	3 (135 patients) [40,41]	2.11 (0.64 to 6.98)	45%
tRNS Vs sham	Response, MDD	1 (66 patients) [44]	0.19 (0.02 to 1.70)	N/A
	Remission, MDD	1 (66 patients) [44]	0.20 (0.01 to 4.33)	N/A
**tDCS Vs medication or psychotherapy**				
tDCS Vs antidepressant	Response, MDD	3 (334 patients) [1,4,22]	0.63 (0.40 to 0.98)	0%
	Remission, MDD	3 (334 patients) [1,4,22]	0.63 (0.39 to 1.01)	0%
tDCS Vs psychotherapy	Response, MDD	1 (18 patients) [26]	0.06 (0.00 to 1.43)	N/A
	Remission, MDD	1 (18 patients) [26]	0.16 (0.01 to 3.81)	N/A
**tDCS Combined with Medications**				
tDCS + antidepressant Vs sham + antidepressant	Response, MDD	7 (396 patients) [5,6,8,10,12,16,24,30]	1.41 (0.77 to 2.61)	23%
	Remission, MDD	10 (496 patients) [5,6,8,10,12,16,20,24,30]	1.29 (0.71 to 2.35)	22%
tDCS + psychotherapy Vs sham + psychotherapy	Response, MDD	4 (179 patients) [17,21,26,29]	1.26 (0.67 to 2.36)	0%
	Remission, MDD	4 (179 patients) [17,21,26,29]	1.50 (0.72 to 3.13)	0%
	Response, MDD	1 (84 patients) [21]	0.76 (0.31 to 1.83)	N/A

© 2025 Ren C et al. *JAMA Network Open*.

tDCS + psychotherapy Vs psychotherapy	Remission, MDD	1 (84 patients) [21]	1.03 (0.42 to 2.54)	N/A
tDCS + psychotherapy Vs sham	Response, MDD	1 (137 patients) [31]	0.76 (0.36 to 1.48)	N/A
	Remission, MDD	1 (137 patients) [31]	0.63 (0.26 to 1.56)	N/A
**tDCS Combined with OBS**				
tDCS + OBS Vs sham + OBS	Response, MDD	2 (136 patients) [27,36]	1.95 (0.86 to 4.41)	N/A

CI = Confidence Interval; DPC = Depression in Psychiatric Comorbidities; DMC = Depression in Medical Comorbidities; I^2 = Heterogeneity index; MDD = Major Depressive Disorder; OBS: other brain stimulation techniques (e.g., electroconvulsive therapy [ECT], cranial electrotherapy stimulation [CET], repetitive transcranial magnetic stimulation [rTMS]); tDCS, transcranial direct current stimulation, tRNS, transcranial random noise stimulation; tES, transcranial electric stimulation; tACS, transcranial alternating current stimulation;

© 2025 Ren C et al. *JAMA Network Open*.

eTable 9. Subgroup analyses of tES studies: follow-up less than 3 months

Subgroup	Comparison	Diagnostic group	Subgroup variable	Conclusion	No. of studies (Sample Size)
Follow-up (Less than 3 months)	**tES Vs sham/no treatment**	Primary depression, all patients	Less than 3 months	SMD: -0.37; 95% CI: -0.71 to -0.03; I^2= 79%	29 (1061 patients)[11-13,15,35,37,38,50,51,63,70,72,74,76,78-80,82,87,40,41]
		Primary depression, MDD	Less than 3 months	SMD: -0.28; 95% CI: -0.78 to 0.23; I^2= 77%	15 (541 patients) [11-13,15,35,37,38,40,41,43]
		Primary depression, DPC	Less than 3 months	SMD: -0.79; 95% CI: -3.24 to 1.65; I^2= 84%	3 (82 patients)[50,51,63]
		Primary depression, DMC	Less than 3 months	SMD: -0.39; 95% CI: -0.99 to 0.22; I^2= 82%	11 (438 patients)[74,76,78-80,82,87]
	tDCS Vs sham/no treatment	Primary depression, all patients	Less than 3 months	SMD: -0.40; 95% CI: -0.79 to -0.01; I^2= 79%	25 (882 patients) [11-13,15,35,37,38,50,51,63,70,72,74,76,78-80,82,87,40,41,43]
		Primary depression, MDD	Less than 3 months	SMD: -0.32; 95% CI: -1.03 to 0.38; I^2= 78%	11 (362 patients) [11-13,15,35,37,38]
		Primary depression, DPC	Less than 3 months	SMD: -0.79; 95% CI: -3.24 to 1.65; I^2= 83%	3 (82 patients) [50,51,63]
		Primary depression, DMC	Less than 3 months	SMD: -0.39; 95% CI: -0.99 to 0.22; I^2= 82%	11 (438 patients) [74,76,78-80,82,87]
	tACS Vs sham	Primary depression, MDD	Less than 3 months	SMD: -0.40; 95% CI: -1.78 to 0.97; I^2= 67%	3 (143 patients)[40,41]
	tRNS Vs sham	Primary depression, MDD	Less than 3 months	SMD: 0.43; 95% CI: -0.23 to 1.09; I^2= N/A	1 (36 patients)[43]
	**tES Vs medication or psychotherapy**				
	tDCS Vs pharmacotherapy	Primary depression, DPC	Less than 3 months	SMD: -1.70; 95% CI: -3.10 to -0.31; I^2= N/A	1 (12 patients)[51]
	tDCS Vs psychotherapy	Primary depression, all patients	Less than 3 months	SMD: 0.20; 95% CI: -3.46 to 3.85; I^2= 0%	2 (48 patients)[26,73]
		Primary depression, MDD	Less than 3 months	SMD: 0.58; 95% CI: -0.37 to 1.53; I^2= N/A	1 (18 patients) [26]
		Primary depression, DMC	Less than 3 months	SMD: -0.02; 95% CI: -0.74 to 0.70; I^2= N/A	1 (30 patients) [73]
	**tDCS Combined with Medications**				
	tDCS + antidepressant Vs sham + antidepressant	Primary depression, MDD	Less than 3 months	SMD: -0.45; 95% CI: -1.10 to -0.21; I^2= 58%	5 (203 patients)[6,10,12,16,30]

© 2025 Ren C et al. *JAMA Network Open*.

	tDCS + pharmacotherapy Vs sham + pharmacotherapy	Primary depression, all patients	Less than 3 months	SMD: -0.61; 95% CI: -1.22 to 0.01; I^2= N/A	1 (43 patients)[80]
	**tDCS Combined with Psychotherapy**				
	tDCS + psychotherapy Vs sham + psychotherapy	Primary depression, MDD	Less than 3 months	SMD: -0.29; 95% CI: -3.69 to 3.11; I^2= 0%.	2 (55 patients)[26,29]
	tDCS + psychotherapy Vs psychotherapy	Primary depression, MDD	Less than 3 months	SMD: -0.86; 95% CI: -1.79 to 0.06; I^2= N/A.	1 (20 patients)[37]
	tDCS + psychotherapy Vs tDCS	Primary depression, MDD	Less than 3 months	SMD: -1.00; 95% CI: -1.75 to -0.25; I^2= N/A.	1 (31 patients)[2]

CI = Confidence Interval; DPC = Depression in Psychiatric Comorbidities; DMC = Depression in Medical Comorbidities. F3 refers to the left dorsolateral prefrontal cortex (DLPFC) based on the 10/20 EEG electrode placement system; I^2 = Heterogeneity index; MDD = Major Depressive Disorder; N/A, Not Applicable; "Other regions" include Fpz (frontopolar), T3 (left temporal), C3 (left central), FC1 (frontal-central), Fp2 (right frontopolar), OFC (orbitofrontal cortex), and occipital cortex. OBS: other brain stimulation techniques (e.g., electroconvulsive therapy [ECT], cranial electrotherapy stimulation [CET], repetitive transcranial magnetic stimulation [rTMS]); SMD = Standardized Mean Difference; tDCS, transcranial direct current stimulation, tRNS, transcranial random noise stimulation; tES, transcranial electric stimulation; tACS, transcranial alternating current stimulation.

© 2025 Ren C et al. *JAMA Network Open*.

eTable 10. Subgroup analyses of tES studies: follow-up more than 3 months

Subgroup	Comparison	Diagnostic group	Subgroup variable	Conclusion	No. of studies (Sample Size)
Follow-up (more than 3 months)	**tES Vs sham**	Primary depression, all patients	More than 3 months	SMD: -0.61; 95% CI: -5.74 to 4.51; I^2= 59%.	2 (71 patients)[66,75]
	tDCS Vs sham	Primary depression, DMC	More than 3 months	SMD: -0.27; 95% CI: -0.85 to 0.31; I^2= N/A.	1 (46 patients)[75]
	tACS Vs sham	Primary depression, DPC	More than 3 months	SMD: -1.08; 95% CI: -1.93 to -0.23; I^2= N/A.	1 (25 patients)[66]
	**tDCS Combined with Medications**				
	tDCS + antidepressant Vs sham + antidepressant	Primary depression, MDD	More than 3 months	SMD: 0.19; 95% CI: -0.13 to 1.50; I^2= 0%	2 (300 patients)[8]
	**tDCS Combined with Psychotherapy**				
	tDCS + psychotherapy Vs sham + psychotherapy	Primary depression, all patients	More than 3 months	SMD: -0.14; 95% CI: -0.80 to 0.52; I^2= 6%.	3 (192 patients)[21,83]
		Primary depression, MDD	More than 3 months	SMD: -0.07; 95% CI: -1.05 to 0.91; I^2= 0%.	2 (170 patients)[21]
		Primary depression, DMC	More than 3 months	SMD: -0.71; 95% CI: -1.57 to 0.16; I^2= N/A.	1 (22 patients)[83]
	tDCS + psychotherapy Vs psychotherapy	Primary depression, all patients	More than 3 months	SMD: -0.16; 95% CI: -0.78 to 0.45; I^2= 0%.	3 (196 patients)[21,52]
		Primary depression, MDD	More than 3 months	SMD: -0.63; 95% CI: -1.39 to 0.13; I^2= N/A.	1 (28 patients)[52]
	tDCS + psychotherapy Vs wait-listing	Primary depression, DPC	More than 3 months	SMD: -4.14; 95% CI: -5.47 to -2.81; I^2= N/A.	1 (30 patients)[52]

CI = Confidence Interval; DPC = Depression in Psychiatric Comorbidities; DMC = Depression in Medical Comorbidities. F3 refers to the left dorsolateral prefrontal cortex (DLPFC) based on the 10/20 EEG electrode placement system; I^2 = Heterogeneity index; MDD = Major Depressive Disorder; "Other regions" include Fpz (frontopolar), T3 (left temporal), C3 (left central), FC1 (frontal-central), Fp2 (right frontopolar), OFC (orbitofrontal cortex), and occipital cortex. OBS: other brain stimulation techniques (e.g., electroconvulsive therapy [ECT], cranial electrotherapy stimulation [CET], repetitive transcranial magnetic stimulation [rTMS]); SMD = Standardized Mean Difference; tDCS, transcranial direct current stimulation; tES, transcranial electric stimulation; tACS, transcranial alternating current stimulation.

© 2025 Ren C et al. *JAMA Network Open*.

eTable 11. Subgroup analysis of tDCS studies: electrode size

Subgroup	Comparison	Diagnostic group	Subgroup variable	Conclusion	No. of studies (Sample Size)
Electrode size: 25 cm^2 or 35 cm^2	**tDCS overall**	Primary depression, all patients	25 cm^2	SMD: -0.64; 95% CI: -0.95 to 0.32; I^2= 80%	32 (1613 patients) [4,11,12,15,23,36,37,5,12,30,3,29,27,36,52,54,61,46,79,82,84,85,73,69]
			35 cm^2	SMD: -0.42; 95% CI: -0.63 to -0.21; I^2= 78%	62 (2556 patients) [1,7,13,14,19,25,28,34,38,19,45,50,51,53,55,57-60,62-65,68,70-72,75-77,80,81,86-88,1,22,26,8-10,16,20,9,10,16,17,20,24,21,2,18,19,45,50,51,53,55,57-60,62-65,51,48,56,68,70-72,75-77,80,81,86-88,80,83]
		Primary depression, MDD	25 cm^2	SMD: -0.48; 95% CI: -0.78 to -0.18; I^2= 67%	19 (1017 patients) [4,11,12,15,23,36,37,5,12,30,3,29,27,36]
			35 cm^2	SMD: 0.01; 95% CI: -0.13 to 0.15; I^2= 36%	29 (1513 patients) [1,7,13,14,19,25,28,34,38,19,45,50,51,53,55,57-60,62-65,68,70-72,75-77,80,81,86-88,1,22,26,8-10,16,20,9,10,16,17,20,24,21,2,18]
		Primary depression, DPC	25 cm^2	SMD: -0.75; 95% CI: -1.96 to -0.46; I^2= 79%	5 (186 patients) [52,54,61,46]
			35 cm^2	SMD: -0.80; 95% CI: -1.14 to -0.46; I^2= 70%	20 (599 patients) [19,45,50,51,53,55,57-60,62-65,51,48,56]
		Primary depression, DMC	25 cm^2	SMD: -0.94; 95% CI: -2.04 to 0.16; I^2= 91%	8 (410 patients) [79,82,84,85,73,69]
			35 cm^2	SMD: -0.86; 95% CI: -1.63 to -0.09; I^2= 87%	13 (444 patients) [68,70-72,75-77,80,81,86-88,80,83]
	**tDCS Vs sham, control, antidepressant, pharmacotherapy, or psychotherapy**				
	tDCS Vs sham/no treatment	Primary depression, all patients	25 cm^2	SMD: -0.74; 95% CI: -1.34 to -0.14; I^2= 87%	16 (899 patients) [11,12,15,23,36,37,54,61,79,82,84,85]
			35 cm^2	SMD: -0.53; 95% CI: -0.84 to -0.22; I^2= 81%	39 (1452 patients) [1,7,13,14,19,25,28,34,38,19,45,50,51,53,55,57-60,62-65,68,70-72,75-77,80,81,86-88]
		Primary depression, MDD	25 cm^2	SMD: -0.50; 95% CI: -1.24 to 0.25; I^2= 64%	8 (476 patients)[11,12,15,23,36,37]
			35 cm^2	SMD: 0.01; 95% CI: -0.23 to 0.26; I^2= 33%	12 (596 patients)[1,7,13,14,19,25,28,34,38]
		Primary depression, DPC	25 cm^2	SMD: -0.20; 95% CI: -1.65 to 1.25; I^2= 0%	2 (69 patients)[54,61]
			35 cm^2	SMD: -0.80; 95% CI: -1.21 to -0.38; I^2= 75%	16 (477 patients)[19,45,50,51,53,55,57-60,62-65]

© 2025 Ren C et al. *JAMA Network Open*.

Subgroup	Comparison	Diagnostic group	Subgroup variable	Conclusion	No. of studies (Sample Size)
		Primary depression, DMC	25 cm^2	SMD: -1.42; 95% CI: -3.32 to 0.48; I^2= 94%	6 (354 patients)[79,82,84,85]
			35 cm^2	SMD: -0.83; 95% CI: -1.76 to 0.09; I^2= 88%	11 (379 patients)[68,70-72,75-77,80,81,86-88]
	tDCS Vs antidepressant	Primary depression, MDD	25 cm^2	SMD: 0.19; 95% CI: -0.33 to 0.71; I^2= N/A	1 (57 studies)[4]
			35 cm^2	SMD: 0.40; 95% CI: -0.86 to 1.67; I^2= 0%	2 (175 studies)[1,22]
	tDCS Vs pharmacotherapy	Primary depression, DPC	35 cm^2	SMD: -1.80; 95% CI: -3.23 to -0.38; I^2= N/A	1 (12 patients)[51]
	tDCS Vs psychotherapy	Primary depression, DMC	25 cm2	SMD: -0.05; 95% CI: -0.77 to 0.67; I^2= N/A	1 (30 patients)[73]
		Primary depression, MDD	35 cm^2	SMD: 0.68; 95% CI: -0.28 to 1.64; I^2= N/A	1 (18 patients)[26]
	**tDCS Combined with Medications**				
	tDCS + antidepressant Vs sham + antidepressant	Primary depression, MDD	25 cm^2	SMD: -0.78; 95% CI: -1.07 to -0.49; I^2= 0%	5 (258 patients)[5,12,30]
			35 cm^2	SMD: -0.03; 95% CI: -0.50 to 0.44; I^2= 44%	6 (303 patients)[8-10,16,20]
	tDCS + pharmacotherapy Vs sham + pharmacotherapy	Primary depression, all patients	35 cm^2	SMD: -0.87; 95% CI: -1.57 to -0.17; I^2= 39%	4 (153 patients) [48,56,80]
		Primary depression, DPC	25 cm^2	SMD: -0.87; 95% CI: -1.40 to -0.33; I^2= N/A	1 (59 patients)[46]
			35 cm^2	SMD: -0.65; 95% CI: -0.80 to -0.51; I^2= 0%	3 (110 patients)[48,56]
		Primary depression, DMC	35 cm^2	SMD: -1.54; 95% CI: -2.23 to 0.85; I^2= N/A	1 (43 patients)[80]
	**tDCS Combined with Psychotherapy**				
	tDCS + psychotherapy Vs sham + psychotherapy	Primary depression, all patients	25 cm^2	SMD: -0.02; 95% CI: -0.23 to 0.19; I^2= 0%	3 (96 patients) [3,29,69]
			35 cm^2	SMD: -0.22; 95% CI: -0.48 to 0.05; I^2= 0%	6 (196 patients) [9,10,16,17,20,24,83]
		Primary depression, MDD	25 cm^2	SMD: 0.02; 95% CI: -0.23 to 0.26; I^2= 0%	2 (70 patients)[3,29]
			35 cm^2	SMD: -0.17; 95% CI: -0.44 to 0.10; I^2= 0%.	5 (194 patients)[9,10,16,17,20,24]

© 2025 Ren C et al. *JAMA Network Open*.

Subgroup	Comparison	Diagnostic group	Subgroup variable	Conclusion	No. of studies (Sample Size)
		Primary depression, DMC	25 cm^2	SMD: -0.13; 95% CI: -0.91 to 0.64; I^2= N/A	1 (26 patients)[69]
			35 cm^2	SMD: -0.61; 95% CI: -1.47 to 0.25; I^2= N/A	1 (22 patients)[83]
	tDCS + psychotherapy Vs psychotherapy	Primary depression, all patients	25 cm^2	SMD: -0.55; 95% CI: -4.79 to 3.69; I^2= 19%.	2 (48 patients) [37,52]
		Primary depression, MDD	25 cm^2	SMD: -0.95; 95% CI: -1.89 to -0.01; I^2= N/A.	1 (20 patients)[37]
			35 cm^2	SMD: -0.29; 95% CI: -0.72 to 0.14; I^2= N/A.	1 (84 patients)[21]
		Primary depression, DPC	25 cm^2	SMD: -0.27; 95% CI: -1.02 to 0.48; I^2= N/A	1 (30 patients)[52]
	tDCS + psychotherapy Vs tDCS	Primary depression, MDD	35 cm^2	SMD: -0.40; 95% CI: -1.11 to 0.31; I^2= N/A.	1 (31 patients)[2]
	tDCS + psychotherapy Vs wait-listing	Primary depression, DPC	25 cm^2	SMD: -2.55; 95% CI: -3.54 to -1.55; I^2= N/A.	1 (30 patients)[52]
	**tDCS Combined with OBS**				
	tDCS + OBS Vs sham + OBS	Primary depression, MDD	25 cm^2	SMD: -0.34; 95% CI: -3.24 to 2.55; I^2= 16%.	2 (136 patients)[27,36]
			35 cm^2	SMD: -0.52; 95% CI: -1.41 to 0.38; I^2= N/A.	1 (20 patients)[18]
	tDCS Vs sham + OBS	Primary depression, MDD	35 cm^2	SMD: -0.12; 95% CI: -0.99 to 0.76; I^2= N/A.	1 (20 patients)[18]

CI = Confidence Interval; DPC = Depression in Psychiatric Comorbidities; DMC = Depression in Medical Comorbidities. F3 refers to the left dorsolateral prefrontal cortex (DLPFC) based on the 10/20 EEG electrode placement system; I^2 = Heterogeneity index; MDD = Major Depressive Disorder; "Other regions" include Fpz (frontopolar), T3 (left temporal), C3 (left central), FC1 (frontal-central), Fp2 (right frontopolar), OFC (orbitofrontal cortex), and occipital cortex. OBS: other brain stimulation techniques (e.g., electroconvulsive therapy [ECT], cranial electrotherapy stimulation [CET], repetitive transcranial magnetic stimulation [rTMS]); SMD = Standardized Mean Difference; tDCS, transcranial direct current stimulation.

© 2025 Ren C et al. *JAMA Network Open*.

eTable 12. Subgroup analysis of tDCS studies: electrode locations

Subgroup	Comparison	Diagnostic group	Subgroup variable	Conclusion	No. of studies (Sample Size)
Electrode locations (F3, "Other regions" include Fpz (frontopolar), T3 (left temporal), C3 (left central), FC1 (frontal-central), Fp2 (right frontopolar), OFC (orbitofrontal cortex), and occipital cortex)	**tDCS overall**	Primary depression, all patients	F3	SMD: -0.58; 95% CI: -0.78 to -0.37; I^2= 83%	87 (4291 patients) [1,7,11-15,19,23,25,28,31,34-39,19,45,47,49,50,53-55,57-65, 46,48,56,52,74-77,81,82,84-87,73,83]
			Other regions	SMD: -0.45; 95% CI: -0.93 to 0.02; I^2= 80%	17 (615 patients) [11,13,35,45,51,52,68,70-72,78-80,73]
		Primary depression, MDD	F3	SMD: -0.25; 95% CI: -0.40 to -0.10; I^2= 67%	51 (2987 patients) [1,7,11-15,19,23,25,28,31,34-39,1,4,22, 26,5,6,8-10,12,16,24,30,3,17,21,26,29,33,31,21,37,2,18,27,36]
			Other regions	SMD: 0.51; 95% CI: -1.15 to 2.17; I^2= 65%	3 (105 patients) [11,13,35]
		Primary depression, DPC	F3	SMD: -0.91; 95% CI: -1.36 to -0.47; I^2= 82%	23 (833 patients) [19,45,47,49,50,53-55,57-65,46,48,56,52]
			Other regions	SMD: -0.83; 95% CI: -2.42 to 0.77; I^2= 63%	4 (66 patients) [45,51,52]
		Primary depression, DMC	F3	SMD: -1.43; 95% CI: -2.34 to -0.52; I^2= 91%	13 (471 patients) [74-77,81,82,84-87,73,83]
			Other regions	SMD: -0.44; 95% CI: -0.89 to 0.01; I^2= 71%	10 (444 patients) [68,70-72,78-80,73,69]
	**tDCS Vs sham/no treatment, antidepressant, pharmacotherapy, or psychotherapy**				
	tDCS Vs sham/no treatment	Primary depression, all patients	F3	SMD: -0.74; 95% CI: -1.05 to -0.42; I^2= 86%	50 (2451 patients) [1,7,11-15,19,23,25,28,31,34-39,19,45,47,49,50,53-55,57-65,74-77,81,82,84-87]
			Other regions	SMD: -0.18; 95% CI: -0.68 to 0.32; I^2= 77%	13 (506 patients) [11,13,35,45,51,68,70-72,78-80]
		Primary depression, MDD	F3	SMD: -0.21; 95% CI: -0.42 to -0.00; I^2= 57%	21 (1362 patients)[1,7,11-15,19,23,25,28,31,34-39]
			Other regions	SMD: 0.51; 95% CI: -1.15 to 2.17; I^2= 65%	3 (105 patients)[11,13,35]
		Primary depression, DPC	F3	SMD: -0.89; 95% CI: -1.44 to -0.33; I^2= 85%	18 (634 patients)[19,45,47,49,50,53-55,57-65]
			Other regions	SMD: -0.84; 95% CI: -13.44 to	2 (26 patients)[45,51]

© 2025 Ren C et al. *JAMA Network Open*.

Subgroup	Comparison	Diagnostic group	Subgroup variable	Conclusion	No. of studies (Sample Size)
				11.76; I^2= 79%	
		Primary depression, DMC	F3	SMD: -1.64; 95% CI: -2.69 to -0.59; I^2= 92%	11 (419 patients)[74-77,81,82,84-87]
			Other regions	SMD: -0.35; 95% CI: -0.81 to 0.11; I^2= 64%	8 (375 patients)[68,70-72,78-80]
	tDCS Vs antidepressant	Primary depression, MDD	F3	SMD: 0.37; 95% CI: -0.00 to 0.74; I^2= 0%	3 (332 patients)[1,4,22]
	tDCS Vs pharmacotherapy	Primary depression, DPC	Other regions	SMD: -1.80; 95% CI: -3.23 to -0.38; I^2= N/A	1 (12 patients)[51]
	tDCS Vs psychotherapy	Primary depression, all patients	F3	SMD: 0.24; 95% CI: -4.30 to 4.79; I^2= 30%	2 (48 patients) [26,73]
		Primary depression, MDD	F3	SMD: 0.68; 95% CI: -0.28 to 1.64; I^2= N/A	1 (18 patients)[26]
		Primary depression, DMC	F3	SMD: -0.05; 95% CI: -0.77 to 0.67; I^2= N/A	1 (30 patients)[73]
	**tDCS Combined with Medications**				
	tDCS + antidepressant Vs sham + antidepressant	Primary depression, MDD	F3	SMD: -0.51; 95% CI: -0.90 to -0.13; I^2= 76%	12 (591 patients)[5,6,8-10,12,16,24,30]
	tDCS + pharmacotherapy Vs sham + pharmacotherapy	Primary depression, DPC	F3	SMD: -0.73; 95% CI: -0.92 to -0.53; I^2= 0%	4 (169 patients)[46,48,56]
		Primary depression, DMC	Other regions	SMD: -1.54; 95% CI: -2.23 to -0.85; I^2= N/A	1 (43 patients)[80]
	**tDCS Combined with Psychotherapy**				
	tDCS + psychotherapy Vs sham + psychotherapy	Primary depression, all patients	F3	SMD: -0.13; 95% CI: -0.32 to 0.05; I^2= 0%	7 (258 patients) [3,17,21,26,29,33,69]
			Other regions	SMD: -0.13; 95% CI: -0.30 to 0.07; I^2= N/A	1 (26 patients)[69]

© 2025 Ren C et al. *JAMA Network Open*.

Subgroup	Comparison	Diagnostic group	Subgroup variable	Conclusion	No. of studies (Sample Size)
		Primary depression, MDD	F3	SMD: -0.11; 95% CI: -0.35 to 0.04; I^2= 0%	6 (236 patients)[3,17,21,26,29,33]
		Primary depression, DMC	F3	SMD: -0.61; 95% CI: -1.47 to 0.25; I^2= N/A	1 (22 patients)[83]
			Other regions	SMD: -0.13; 95% CI: -0.91 to 0.64; I^2= N/A	1 (26 patients)[69]
	tDCS + psychotherapy Vs sham	Primary depression, MDD	F3	SMD: -0.04; 95% CI: -0.37 to 0.30; I^2= N/A.	1 (137 patients)[31]
	tDCS + psychotherapy Vs psychotherapy	Primary depression, MDD	F3	SMD: -0.48; 95% CI: -4.29 to 3.32; I^2= 36%.	2 (104 patients)[21,37]
		Primary depression, DPC	Other regions	SMD: -0.27; 95% CI: -1.02 to 0.48; I^2= N/A.	1 (28 patients)[52]
	tDCS + psychotherapy Vs tDCS	Primary depression, MDD	F3	SMD: -0.40; 95% CI: -1.11 to 0.31; I^2= N/A.	1 (31 patients)[2]
	tDCS + psychotherapy Vs wait-listing	Primary depression, DPC	F3	SMD: -2.55; 95% CI: -3.54 to -1.55; I^2= N/A.	1 (30 patients)[52]
	**tDCS Combined with OBS**				
	tDCS + OBS Vs sham + OBS	Primary depression, MDD	F3	SMD: -0.33; 95% CI: -0.91 to 0.24; I^2= 0%.	3 (156 patients)[18,27,36]
	tDCS Vs sham + OBS	Primary depression, MDD	F3	SMD: -0.12; 95% CI: -0.99 to 0.76; I^2= N/A.	1 (20 patients)[18]

CI = Confidence Interval; DPC = Depression in Psychiatric Comorbidities; DMC = Depression in Medical Comorbidities. F3 refers to the left dorsolateral prefrontal cortex (DLPFC) based on the 10/20 EEG electrode placement system; I² = Heterogeneity index; MDD = Major Depressive Disorder; "Other regions" include Fpz (frontopolar), T3 (left temporal), C3 (left central), FC1 (frontal-central), Fp2 (right frontopolar), OFC (orbitofrontal cortex), and occipital cortex. OBS: other brain stimulation techniques (e.g., electroconvulsive therapy [ECT], cranial electrotherapy stimulation [CET], repetitive transcranial magnetic stimulation [rTMS]); SMD = Standardized Mean Difference; tDCS, transcranial direct current stimulation.

© 2025 Ren C et al. *JAMA Network Open*.

eTable 13. Subgroup analysis of tDCS studies: treatment settings

Subgroup	Comparison	Diagnostic group	Subgroup variable	Conclusion	No. of studies (Sample Size)
Treatment settings: Home-based or clinic-based	**tDCS Vs sham**	Primary depression, all patients	Home-based	SMD: -0.10; 95% CI: -0.46 to 0.26; I^2= 0%	3(229 patients) [31,34,75]
			Clinic-based	SMD: -0.66; 95% CI: -0.95 to -0.37; I^2= 86%	58 (2592 patients) [1,7,11-15,19,23,25,28,31,34-39,45,47,49-51,53-55,57-65,68,70-72,74-82,84-87]

CI = Confidence Interval; I² = Heterogeneity index; MDD = Major Depressive Disorder; SMD = Standardized Mean Difference; tDCS, transcranial direct current stimulation.

© 2025 Ren C et al. *JAMA Network Open*.

eReferences.

1. Brunoni AR, Moffa AH, Sampaio-Junior B, et al. Trial of Electrical Direct-Current Therapy versus Escitalopram for Depression. *New England Journal of Medicine*. 2017;376(26):2523-2533. doi:https://dx.doi.org/10.1056/NEJMoa1612999
2. Monnart A, Vanderhasselt M-A, Schroder E, Campanella S, Fontaine P, Kornreich C. Treatment of Resistant Depression: A Pilot Study Assessing the Efficacy of a tDCS-Mindfulness Program Compared With a tDCS-Relaxation Program. *Frontiers in psychiatry Frontiers Research Foundation*. 2019;10:730. doi:https://dx.doi.org/10.3389/fpsyt.2019.00730
3. Vanderhasselt M-A, De Raedt R, Namur V, et al. Transcranial electric stimulation and neurocognitive training in clinically depressed patients: a pilot study of the effects on rumination. *Progress in Neuro-Psychopharmacology & Biological Psychiatry*. 2015;57:93-9. doi:https://dx.doi.org/10.1016/j.pnpbp.2014.09.015
4. Bares M, Brunovsky M, Stopkova P, Hejzlar M, Novak T. Transcranial Direct-Current Stimulation (tDCS) Versus Venlafaxine ER In The Treatment Of Depression: A Randomized, Double-Blind, Single-Center Study With Open-Label, Follow-Up. *Neuropsychiatric Disease & Treatment*. 2019;15:3003-3014. doi:https://dx.doi.org/10.2147/NDT.S226577
5. Pavlova EL, Menshikova AA, Semenov RV, et al. Transcranial direct current stimulation of 20- and 30-minutes combined with sertraline for the treatment of depression. *Progress in Neuro-Psychopharmacology & Biological Psychiatry*. 2018;82:31-38. doi:https://dx.doi.org/10.1016/j.pnpbp.2017.12.004
6. Sharafi E, Taghva A, Arbabi M, Dadarkhah A, Ghaderi J. Transcranial Direct Current Stimulation for Treatment-Resistant Major Depression: A Double-Blind Randomized Sham-Controlled Trial. *Clinical EEG & Neuroscience: Official Journal of the EEG & Clinical Neuroscience Society (ENCS)*. 2019;50(6):375-382. doi:https://dx.doi.org/10.1177/1550059419863209
7. Loo CK, Alonzo A, Martin D, Mitchell PB, Galvez V, Sachdev P. Transcranial direct current stimulation for depression: 3-week, randomised, sham-controlled trial. *British Journal of Psychiatry*. 2012;200(1):52-9. doi:https://dx.doi.org/10.1192/bjp.bp.111.097634
8. Burkhardt G, Kumpf U, Crispin A, et al. Transcranial direct current stimulation as an additional treatment to selective serotonin reuptake inhibitors in adults with major depressive disorder in Germany (DepressionDC): a triple-blind, randomised, sham-controlled, multicentre trial. *Lancet*. 2023;402(10401):545-554. doi:https://dx.doi.org/10.1016/S0140-6736(23)00640-2
9. Palm U, Schiller C, Fintescu Z, et al. Transcranial direct current stimulation in treatment resistant depression: a randomized double-blind, placebo-controlled study. *Brain Stimulation*. 2012, 5(3): 242–251. doi:10.1016/j.brs.2011.08.005
10. Moirand R, Imbert L, Haesebaert F, et al. Ten Sessions of 30 Min tDCS over 5 Days to Achieve Remission in Depression: A Randomized Pilot Study. *Journal of Clinical Medicine*. 2022;11(3):31. doi:https://dx.doi.org/10.3390/jcm11030782
11. Huang H, Chen Y, Kong S, et al. Targeting right orbitofrontal cortex (OFC) with transcranial direct current stimulation (tDCS) can improve cognitive executive function in a major depressive episode, but not depressive mood: A Double-blind Randomized Controlled Pilot Trial. *Journal of Psychiatric Research*. 2023;168:108-117. doi:https://dx.doi.org/10.1016/j.jpsychires.2023.10.016
12. Brunoni AR, Valiengo L, Baccaro A, et al. The sertraline vs. electrical current therapy for treating depression clinical study: results from a factorial, randomized, controlled trial. *JAMA psychiatry*. 2013;70(4):383-91. doi:https://dx.doi.org/10.1001/2013.jamapsychiatry.32
13. Boggio PS, Rigonatti SP, Ribeiro RB, et al. A randomized, double-blind clinical trial on the efficacy of cortical direct current stimulation for the treatment of major depression. *International Journal of Neuropsychopharmacology*. 2008;11(2):249-54.

© 2025 Ren C et al. *JAMA Network Open*.

14. Blumberger DM, Tran LC, Fitzgerald PB, Hoy KE, Daskalakis ZJ. A randomized double-blind sham-controlled study of transcranial direct current stimulation for treatment-resistant major depression. *Frontiers in psychiatry Frontiers Research Foundation*. 2012;3:74. doi:https://dx.doi.org/10.3389/fpsyt.2012.00074
15. Ribeiro RB, Generoso MB, Taiar IT, et al. Randomized clinical trial on the efficacy of a new transcranial direct current stimulation (tDCS) device in the treatment of depression: a low-cost option for developing countries? *Jornal Brasileiro de Psiquiatria*. 2023;72(4):205-212. doi:https://dx.doi.org/10.1590/0047-2085000000429
16. Bennabi D, Nicolier M, Monnin J, et al. Pilot study of feasibility of the effect of treatment with tDCS in patients suffering from treatment-resistant depression treated with escitalopram. *Clinical Neurophysiology*. 2015;126(6):1185-1189. doi:https://dx.doi.org/10.1016/j.clinph.2014.09.026
17. Nord CL, Halahakoon DC, Limbachya T, et al. Neural predictors of treatment response to brain stimulation and psychological therapy in depression: a double-blind randomized controlled trial. *Neuropsychopharmacology*. 2019;44(9):1613-1622. doi:https://dx.doi.org/10.1038/s41386-019-0401-0
18. Dastjerdi G, Mirhoseini H, Mohammadi E. Investigating the synergistic effects of transcranial direct current stimulation and cranial electrical stimulation in treatment of major depression in a double blinded controlled trial. *Biomedical and Pharmacology Journal*. 2015;8(2):1267-1274. doi:https://dx.doi.org/10.13005/bpj/885
19. Loo CK, Husain MM, McDonald WM, et al. International randomized-controlled trial of transcranial Direct Current Stimulation in depression. *Brain Stimulation*. 2018;11(1):125-133. doi:https://dx.doi.org/10.1016/j.brs.2017.10.011
20. Bennabi D, Carvalho N, Bisio A, Teti Mayer J, Pozzo T, Haffen E. Influence of Transcranial Direct Current Stimulation on Psychomotor Symptoms in Major Depression. *Brain Sciences*. 2020;10(11):29. doi:https://dx.doi.org/10.3390/brainsci10110792
21. Aust S, Brakemeier E-L, Spies J, et al. Efficacy of Augmentation of Cognitive Behavioral Therapy With Transcranial Direct Current Stimulation for Depression: A Randomized Clinical Trial. *JAMA psychiatry*. 2022;79(6):528-537. doi:https://dx.doi.org/10.1001/jamapsychiatry.2022.0696
22. Park S, Choi W-J, Kim S, et al. Effects of transcranial direct current stimulation using miniaturized devices vs sertraline for depression in Korea: A 6 week, multicenter, randomized, double blind, active-controlled study. *Journal of Psychiatric Research*. 2020;127:42-47. doi:https://dx.doi.org/10.1016/j.jpsychires.2020.04.012
23. Zhou Q, Yu C, Yu H, et al. The effects of repeated transcranial direct current stimulation on sleep quality and depression symptoms in patients with major depression and insomnia. *Sleep Medicine*. 2020;70:17-26. doi:https://dx.doi.org/10.1016/j.sleep.2020.02.003
24. Oh J, Jang K-I, Jeon S, Chae J-H. Effect of Self-administered Transcranial Direct Stimulation in Patients with Major Depressive Disorder: A Randomized, Single-blinded Clinical Trial. *Clinical Psychopharmacology & Neuroscience*. 2022;20(1):87-96. doi:https://dx.doi.org/10.9758/cpn.2022.20.1.87
25. Loo CK, Sachdev P, Martin D, et al. A double-blind, sham-controlled trial of transcranial direct current stimulation for the treatment of depression. *International Journal of Neuropsychopharmacology*. 2010;13(1):61-9. doi:https://dx.doi.org/10.1017/S1461145709990411
26. Segrave RA, Arnold S, Hoy K, Fitzgerald PB. Concurrent cognitive control training augments the antidepressant efficacy of tDCS: a pilot study. *Brain Stimulation*. 2014;7(2):325-31. doi:https://dx.doi.org/10.1016/j.brs.2013.12.008
27. Mayur P, Howari R, Byth K, Vannitamby R. Concomitant Transcranial Direct Current Stimulation With Ultrabrief Electroconvulsive Therapy: A 2-Week Double-Blind Randomized Sham-

Controlled Trial. *Journal of ECT*. 2018;34(4):291-295. doi:https://dx.doi.org/10.1097/YCT.0000000000000479
28. Chen Y, Wu C, Lyu D, et al. Comparison of 60-minute vs 30-minute transcranial direct current stimulation (tDCS) in major depressive disorder: Effects on depression suicidal ideation and anxiety. *Psychiatry Research*. 2023;330:115556. doi:https://dx.doi.org/10.1016/j.psychres.2023.115556
29. Brunoni AR, Boggio PS, De Raedt R, et al. Cognitive control therapy and transcranial direct current stimulation for depression: a randomized, double-blinded, controlled trial. *Journal of Affective Disorders*. 2014;162:43-9. doi:https://dx.doi.org/10.1016/j.jad.2014.03.026
30. Kumari B, Singh A, Kar SK, Tripathi A, Agarwal V. Bifrontal-transcranial direct current stimulation as an early augmentation strategy in major depressive disorder: A single-blind randomised controlled trial. *Asian Journal of Psychiatry*. 2023;86:103637. doi:https://dx.doi.org/10.1016/j.ajp.2023.103637
31. Borrione L, Cavendish BA, Aparicio LVM, et al. Home-Use Transcranial Direct Current Stimulation for the Treatment of a Major Depressive Episode: A Randomized Clinical Trial. Randomized Controlled Trial. *AMA Psychiatry*. 2024;81(4):329-337. doi:https://dx.doi.org/10.1001/jamapsychiatry.2023.4948
32. Gehrman PR, Bartky EJ, Travers C, Lapidus K. A Fully Remote Randomized Trial of Transcranial Alternating Current Stimulation for the Acute Treatment of Major Depressive Disorder. Journal Article Randomized Controlled Trial Research Support, Non-U.S. Gov't Research Support, N.I.H., Extramural. *J Clin Psychiatry*. Apr 22 2024;85(2):22. doi:https://dx.doi.org/10.4088/JCP.23m15078

33. Ha J, Fang Y, Cron GO, et al. In patients with late-life depression and cognitive decline, adding tDCS to cognitive training does not significantly affect depressive symptoms but shows potential benefits on cognition as measured by fMRI. Empirical Study; Quantitative Study. *Brain Stimul*. Mar-Apr 2024;17(2):202-204. doi:https://dx.doi.org/10.1016/j.brs.2024.02.009
34. Kim J, Park S, Kim H, Roh D, Kim DH. Home-based, Remotely Supervised, 6-Week tDCS in Patients With Both MCI and Depression: A Randomized Double-Blind Placebo-Controlled Trial. Journal Article. Randomized Controlled Trial. *Clin EEG Neurosci*. Sep 2024;55(5):531-542. doi:https://dx.doi.org/10.1177/15500594231215847
35. Kong S, Chen Y, Huang H, et al. Efficacy of transcranial direct current stimulation for treating anhedonia in patients with depression: A randomized, double-blind, sham-controlled clinical trial. Randomized Controlled Trial. Journal Article. *J Affect Disord*. Apr 01 2024;350:264-273. doi:https://dx.doi.org/10.1016/j.jad.2024.01.041
36. Li X, Liu J, Wei S, et al. Cognitive enhancing effect of rTMS combined with tDCS in patients with major depressive disorder: a double-blind, randomized, sham-controlled study. Journal Article. Randomized Controlled Trial. *BMC Med*. Jun 20 2024;22(1):253. doi:https://dx.doi.org/10.1186/s12916-024-03443-7
37. Nejati V, Nozari M, Mirzaian B, Pourshahriar H, Salehinejad MA. Comparable Efficacy of Repeated Transcranial Direct Current Stimulation, Cognitive Behavioral Therapy, and Their Combination in Improvement of Cold and Hot Cognitive Functions and Amelioration of Depressive Symptoms. Randomized Controlled Trial. Journal Article. *J Nerv Ment Dis*. Mar 01 2024;212(3):141-151. doi:https://dx.doi.org/10.1097/NMD.0000000000001745
38. Tabasi M, Mostafavi S-A, Oreyzi H, Mohammadi MR, Khaleghi A. The Effectiveness of Transcranial Direct Current Stimulation (tDCS) and Omega-3 on Food Craving, Executive Functions, Weight, and Depressive Symptoms in Patients with Depression and Overweight: A Randomized

© 2025 Ren C et al. *JAMA Network Open*.

Controlled Trial. Journal Article. *Iran*. Feb 2024;19(2):158-173. doi:https://dx.doi.org/10.18502/ijps.v19i2.15102
39. Woodham RD, Selvaraj S, Lajmi N, et al. Home-based transcranial direct current stimulation RCT in major depression. Preprint.*medRxiv*.2023;27. doi:https://dx.doi.org/10.1101/2023.11.27.23299059
40. Wang H, Wang K, Xue Q, et al. Transcranial alternating current stimulation for treating depression: a randomized controlled trial. *Brain*. 2022;145(1):83-91. doi:https://dx.doi.org/10.1093/brain/awab252
41. Alexander ML, Alagapan S, Lugo CE, et al. Double-blind, randomized pilot clinical trial targeting alpha oscillations with transcranial alternating current stimulation (tACS) for the treatment of major depressive disorder (MDD). *Transl Psychiatry Psychiatry*. 2019;9(1):106. doi:https://dx.doi.org/10.1038/s41398-019-0439-0
42. Zhou J, Li D, Ye F, et al. Effect of add-on transcranial alternating current stimulation (tACS) in major depressive disorder: A randomized controlled trial. Journal Article Randomized Controlled Trial. *Brain Stimul*. Jul-Aug 2024;17(4):760-768. doi:https://dx.doi.org/10.1016/j.brs.2024.06.004
43. Schecklmann M, Nejati V, Poeppl TB, et al. Bifrontal high-frequency transcranial random noise stimulation is not effective as an add-on treatment in depression. *Journal of Psychiatric Research*. 2021;132:116-122. doi:https://dx.doi.org/10.1016/j.jpsychires.2020.10.011
44. Nikolin S, Alonzo A, Martin D, et al. Transcranial Random Noise Stimulation for the Acute Treatment of Depression: A Randomized Controlled Trial. *International Journal of Neuropsychopharmacology*. 2020;23(3):146-156. doi:https://dx.doi.org/10.1093/ijnp/pyz072
45. Shafiezadeh S, Eshghi M, Dokhaei Z, et al. Effect of Transcranial Direct Current Stimulation on Dorsolateral Prefrontal Cortex to Reduce the Symptoms of the Obsessive-Compulsive Disorder. *Basic and Clinical Neuroscience*. 2021;12(5):675-680. doi:https://dx.doi.org/10.32598/bcn.2021.1920.2
46. Sampaio-Junior B, Tortella G, Borrione L, et al. Efficacy and Safety of Transcranial Direct Current Stimulation as an Add-on Treatment for Bipolar Depression: A Randomized Clinical Trial. *JAMA psychiatry*. 2018;75(2):158-166. doi:https://dx.doi.org/10.1001/jamapsychiatry.2017.4040
47. Lee J, Lee CW, Jang Y, et al. Efficacy and safety of daily home-based transcranial direct current stimulation as adjunct treatment for bipolar depressive episodes: Double-blind sham-controlled randomized clinical trial. *Frontiers in psychiatry Frontiers Research Foundation*. 2022;13:969199. doi:https://dx.doi.org/10.3389/fpsyt.2022.969199
48. Mardani P, Zolghadriha A, Dadashi M, Javdani H, Mousavi SE. Effect of medication therapy combined with transcranial direct current stimulation on depression and response inhibition of patients with bipolar disorder type I: a clinical trial. *BMC Psychiatry*. 2021;21(1):579. doi:https://dx.doi.org/10.1186/s12888-021-03592-6
49. Zhang L, Li Q, Du Y, et al. Effect of high-definition transcranial direct current stimulation on improving depression and modulating functional activity in emotion-related cortical-subcortical regions in bipolar depression. *Journal of Affective Disorders*. 2023;323:570-580. doi:https://dx.doi.org/10.1016/j.jad.2022.12.007
50. de Lima AL, Braga FMA, da Costa RMM, Gomes EP, Brunoni AR, Pegado R. Transcranial direct current stimulation for the treatment of generalized anxiety disorder: A randomized clinical trial. *Journal of Affective Disorders*. 2019;259:31-37. doi:https://dx.doi.org/10.1016/j.jad.2019.08.020
51. Movahed FS, Goradel JA, Pouresmali A, Mowlaie M. Effectiveness of transcranial direct current stimulation onworry, anxiety, and depression in generalized anxiety disorder: A randomized, single-blind pharmacotherapy and sham-controlled clinical trial. *Iranian Journal of Psychiatry and Behavioral Sciences*. 2018;12(2) (no pagination)doi:https://dx.doi.org/10.5812/ijpbs.11071

52. Nasiri F, Mashhadi A, Bigdeli I, Chamanabad AG, Ellard KK. Augmenting the unified protocol for transdiagnostic treatment of emotional disorders with transcranial direct current stimulation in individuals with generalized anxiety disorder and comorbid depression: A randomized controlled trial. *Journal of Affective Disorders*. 2020;262:405-413. doi:https://dx.doi.org/10.1016/j.jad.2019.11.064
53. Palm U, Keeser D, Hasan A, et al. Prefrontal Transcranial Direct Current Stimulation for Treatment of Schizophrenia With Predominant Negative Symptoms: A Double-Blind, Sham-Controlled Proof-of-Concept Study. *Schizophrenia Bulletin*. 2016;42(5):1253-61. doi:https://dx.doi.org/10.1093/schbul/sbw041
54. Jeon D-W, Jung D-U, Kim S-J, et al. Adjunct transcranial direct current stimulation improves cognitive function in patients with schizophrenia: A double-blind 12-week study. *Schizophrenia Research*. 2018;197:378-385. doi:https://dx.doi.org/10.1016/j.schres.2017.12.009
55. Lisoni J, Nibbio G, Baldacci G, et al. Improving depressive symptoms in patients with schizophrenia using bilateral bipolar-nonbalanced prefrontal tDCS: Results from a double-blind sham-controlled trial. Randomized Controlled Trial. Journal Article. *J Affect Disord*. Mar 15 2024;349:165-175. doi:https://dx.doi.org/10.1016/j.jad.2024.01.050
56. Taremian F, Nazari S, Moradveisi L, Moloodi R. Transcranial Direct Current Stimulation on Opium Craving, Depression, and Anxiety: A Preliminary Study. *Journal of ECT*. 2019;35(3):201-206. doi:https://dx.doi.org/10.1097/YCT.0000000000000568
57. Naeim M, Rezaeisharif A, Moghadam SA. Reduce depression and anxiety in methadone users with transcranial direct current stimulation. *Iranian Journal of Psychiatry and Behavioral Sciences*. 2021;15(1) (no pagination)doi:https://dx.doi.org/10.5812/IJPBS.98062
58. Batista EK, Klauss J, Fregni F, Nitsche MA, Nakamura-Palacios EM. A Randomized Placebo-Controlled Trial of Targeted Prefrontal Cortex Modulation with Bilateral tDCS in Patients with Crack-Cocaine Dependence. *International Journal of Neuropsychopharmacology*. 2015;18(12):10. doi:https://dx.doi.org/10.1093/ijnp/pyv066
59. Eskandari Z, Dadashi M, Mostafavi H, Kia AA, Pirzeh R. Research paper: Comparing the efficacy of anodal, cathodal, and sham transcranial direct current stimulation on brain-derived neurotrophic factor and psychological symptoms in opioid-addicted patients. *Basic and Clinical Neuroscience*. 2019;10(6):641-650. doi:https://dx.doi.org/10.32598/BCN.9.10.435
60. Sadeghi Bimorgh M, Omidi A, Ghoreishi FS, Rezaei Ardani A, Ghaderi A, Banafshe HR. The Effect of Transcranial Direct Current Stimulation on Relapse, Anxiety, and Depression in Patients With Opioid Dependence Under Methadone Maintenance Treatment: A Pilot Study. *Frontiers in Pharmacology*. 2020;11:401. doi:https://dx.doi.org/10.3389/fphar.2020.00401
61. Van Noppen P, van Dun K, Depestele S, Verstraelen S, Meesen R, Manto M. Transcranial direct current stimulation and attention skills in burnout patients: a randomized blinded sham-controlled pilot study. *F1000Research*. 2020;9:116. doi:https://dx.doi.org/10.12688/f1000research.21831.2
62. Wilczek-Ruzyczka E, Gawronska A, Goral-Polrola J. An evaluation of transcranial direct current stimulation (Tdcs) in the reduction of occupational burnout syndrome in nurses. *Acta Neuropsychologica*. 2021;19(2):169-185.
63. Ahmadizadeh MJ, Rezaei M, Fitzgerald PB. Transcranial direct current stimulation (tDCS) for post-traumatic stress disorder (PTSD): A randomized, double-blinded, controlled trial. *Brain Research Bulletin*. 2019;153:273-278. doi:https://dx.doi.org/10.1016/j.brainresbull.2019.09.011
64. Vigod SN, Murphy KE, Dennis C-L, et al. Transcranial direct current stimulation (tDCS) for depression in pregnancy: A pilot randomized controlled trial. *Brain Stimulation*. 2019;12(6):1475-1483. doi:https://dx.doi.org/10.1016/j.brs.2019.06.019

© 2025 Ren C et al. *JAMA Network Open*.

65. Lisoni J, Miotto P, Barlati S, et al. Change in core symptoms of borderline personality disorder by tDCS: A pilot study. *Psychiatry Research*. 2020;291:113261. doi:https://dx.doi.org/10.1016/j.psychres.2020.113261
66. Perera MPN, Bailey NW, Murphy OW, et al. Home-Based Individualized Alpha Transcranial Alternating Current Stimulation Improves Symptoms of Obsessive-Compulsive Disorder: Preliminary Evidence from a Randomized, Sham-Controlled Clinical Trial. *Depression and Anxiety*. 2023;2023 (no pagination)doi:https://dx.doi.org/10.1155/2023/9958884
67. Zhang M, Force RB, Walker C, Ahn S, Jarskog LF, Frohlich F. Alpha transcranial alternating current stimulation reduces depressive symptoms in people with schizophrenia and auditory hallucinations: a double-blind, randomized pilot clinical trial. *Schizophrenia (Heidelberg, Germany)*. 2022;8(1):114. doi:https://dx.doi.org/10.1038/s41537-022-00321-0
68. Khedr EM, Salama RH, Abdel Hameed M, Abo Elfetoh N, Seif P. Therapeutic Role of Transcranial Direct Current Stimulation in Alzheimer Disease Patients: Double-Blind, Placebo-Controlled Clinical Trial. *Neurorehabilitation & Neural Repair*. 2019;33(5):384-394. doi:https://dx.doi.org/10.1177/1545968319840285
69. Brooks H, Oughli HA, Kamel L, et al. Enhancing Cognition in Older Persons with Depression or Anxiety with a Combination of Mindfulness-Based Stress Reduction (MBSR) and Transcranial Direct Current Stimulation (tDCS): Results of a Pilot Randomized Clinical Trial. *Mindfulness*. 2021;12(12):3047-3059. doi:https://dx.doi.org/10.1007/s12671-021-01764-9
70. Mariano TY, Burgess FW, Bowker M, et al. Transcranial Direct Current Stimulation for Affective Symptoms and Functioning in Chronic Low Back Pain: A Pilot Double-Blinded, Randomized, Placebo-Controlled Trial. *Pain Medicine*. 2019;20(6):1166-1177. doi:https://dx.doi.org/10.1093/pm/pny188
71. Quintiliano A, Bikson M, Oehmen T, Pegado R, Kirsztajn GM. Transcranial Direct Current Stimulation (tDCS): Pain Management in End-Stage Renal Disease - Report of an Early Randomized Controlled Trial. *Journal of Pain & Symptom Management*. 2022;64(3):234-243.e1. doi:https://dx.doi.org/10.1016/j.jpainsymman.2022.05.018
72. Ibrahim NM, Abdelhameed KM, Kamal SMM, Khedr EMH, Kotb HIM. Effect of Transcranial Direct Current Stimulation of the Motor Cortex on Visceral Pain in Patients with Hepatocellular Carcinoma. *Pain Medicine*. 2018;19(3):550-560. doi:https://dx.doi.org/10.1093/pm/pnx087
73. Gueserse M, Zali A, Hassanzadeh S, Hatami M, Ahadi M. A Comparative Study of the Effectiveness of Acceptance and Commitment Therapy and Transcranial Direct Current Stimulation on Anxiety, Depression, and Physical Symptoms of Individuals Suffering from Chronic Pain. *Journal of Biomolecular Techniques*. 2022;33(1):11-21. doi:https://dx.doi.org/10.22122/ijbmc.v9i1.336
74. Kim S, Salazar Fajardo JC, Seo E, Gao C, Kim R, Yoon B. Effects of transcranial direct current stimulation on physical and mental health in older adults with chronic musculoskeletal pain: a randomized controlled trial. *European geriatric medicine*. 2022;13(4):959-966. doi:https://dx.doi.org/10.1007/s41999-022-00626-4
75. Mota SM, Amaral de Castro L, Riedel PG, et al. Home-Based Transcranial Direct Current Stimulation for the Treatment of Symptoms of Depression and Anxiety in Temporal Lobe Epilepsy: A Randomized, Double-Blind, Sham-Controlled Clinical Trial. *Frontiers in Integrative Neuroscience*. 2021;15:753995. doi:https://dx.doi.org/10.3389/fnint.2021.753995
76. Liu A, Bryant A, Jefferson A, et al. Exploring the efficacy of a 5-day course of transcranial direct current stimulation (TDCS) on depression and memory function in patients with well-controlled temporal lobe epilepsy. *Epilepsy & Behavior*. 2016;55:11-20. doi:https://dx.doi.org/10.1016/j.yebeh.2015.10.032
77. Azmoodeh S, Soleimani E, Issazadegan A. The Effects of Transcranial Direct Current Stimulation on Depression, Anxiety, and Stress in Patients with Epilepsy: A Randomized Clinical

© 2025 Ren C et al. *JAMA Network Open*.

Trial. *Iranian journal of medical sciences*. 2021;46(4):272-280. doi:https://dx.doi.org/10.30476/ijms.2020.83233.1215
78. Khedr EM, Omran EAH, Ismail NM, et al. Effects of transcranial direct current stimulation on pain, mood and serum endorphin level in the treatment of fibromyalgia: A double blinded, randomized clinical trial. *Brain Stimulation*. 2017;10(5):893-901. doi:https://dx.doi.org/10.1016/j.brs.2017.06.006
79. Arroyo-Fernandez R, Avendano-Coy J, Velasco-Velasco R, Palomo-Carrion R, Bravo-Esteban E, Ferri-Morales A. Effectiveness of Transcranial Direct Current Stimulation Combined With Exercising in People With Fibromyalgia: A Randomized Sham-Controlled Clinical Trial. *Archives of Physical Medicine & Rehabilitation*. 2022;103(8):1524-1532. doi:https://dx.doi.org/10.1016/j.apmr.2022.02.020
80. Paula TMHd, Castro MS, Medeiros LF, et al. Association of low-dose naltrexone and transcranial direct current stimulation in fibromyalgia: a randomized, double-blinded, parallel clinical trial. *Brazilian Journal of Anesthesiology*. 2023;73(4):409-417. doi:https://dx.doi.org/10.1016/j.bjane.2022.08.003
81. Szymkowicz SM, Taylor WD, Woods AJ. Augmenting cognitive training with bifrontal tDCS decreases subclinical depressive symptoms in older adults: Preliminary findings. *Brain Stimulation*. 2022;15(5):1037-1039. doi:https://dx.doi.org/10.1016/j.brs.2022.07.055
82. Chalah MA, Grigorescu C, Padberg F, Kumpfel T, Palm U, Ayache SS. Bifrontal transcranial direct current stimulation modulates fatigue in multiple sclerosis: a randomized sham-controlled study. *Journal of Neural Transmission*. 2020;127(6):953-961. doi:https://dx.doi.org/10.1007/s00702-020-02166-2
83. Manenti R, Cotelli MS, Cobelli C, et al. Transcranial direct current stimulation combined with cognitive training for the treatment of Parkinson Disease: A randomized, placebo-controlled study. *Brain Stimulation*. 2018;11(6):1251-1262. doi:https://dx.doi.org/10.1016/j.brs.2018.07.046
84. Zanardi R, Poletti S, Prestifilippo D, Attanasio F, Barbini B, Colombo C. Transcranial direct current stimulation: A novel approach in the treatment of vascular depression. *Brain Stimulation*. 2020;13(6):1559-1565. doi:https://dx.doi.org/10.1016/j.brs.2020.08.013
85. Valiengo LCL, Goulart AC, de Oliveira JF, Bensenor IM, Lotufo PA, Brunoni AR. Transcranial direct current stimulation for the treatment of post-stroke depression: results from a randomised, sham-controlled, double-blinded trial. *Journal of Neurology, Neurosurgery & Psychiatry*. 2017;88(2):170-175. doi:https://dx.doi.org/10.1136/jnnp-2016-314075
86. An T-G, Kim S-H, Kim K-U. Effect of transcranial direct current stimulation of stroke patients on depression and quality of life. *Journal of physical therapy science*. 2017;29(3):505-507. doi:https://dx.doi.org/10.1589/jpts.29.505
87. Pinto ACPN, Piva SR, Vieira AGdS, et al. Transcranial direct current stimulation for fatigue in patients with Sjogren's syndrome: A randomized, double-blind pilot study. *Brain Stimulation*. 2021;14(1):141-151. doi:https://dx.doi.org/10.1016/j.brs.2020.12.004
88. Curatolo M, La Bianca G, Cosentino G, et al. Motor cortex tRNS improves pain, affective and cognitive impairment in patients with fibromyalgia: preliminary results of a randomised sham-controlled trial. *Clinical & Experimental Rheumatology*. 2017;35 Suppl 105(3):100-105.

© 2025 Ren C et al. *JAMA Network Open*.
