# Supplementary material for: Transcranial Electrical Stimulation in Treatment of Depression: A Systematic Review and Meta-Analysis
Source: JAMA Netw Open. 2025 Jun 18;8(6):e2516459. doi: 10.1001/jamanetworkopen.2025.16459 (PMC12177679; doi:10.1001/jamanetworkopen.2025.16459)
Supplement: Supplement 2. — Data Sharing Statement [file jamanetwopen-e2516459-s002.pdf]

Data Sharing Statement

Ren. Transcranial Electrical Stimulation in Depression Management. *JAMA Netw Open*. Published June 18, 2025. doi:10.1001/jamanetworkopen.2025.16459

Data
Data available: No

Additional Information
Explanation for why data not available: This meta-analysis is based on data extracted from publicly available studies. All data used in the analysis were obtained from published articles, and no individual-level participant data were collected. The datasets generated or analyzed during the study are available upon reasonable request.
